# Population genomics and evolution of a fungal pathogen after releasing exotic strains to control insect pests for 20 years

**DOI:** 10.1038/s41396-020-0620-8

**Published:** 2020-02-28

**Authors:** Lijuan Mei, Mingjun Chen, Yanfang Shang, Guirong Tang, Ye Tao, Liang Zeng, Bo Huang, Zengzhi Li, Shuai Zhan, Chengshu Wang

**Affiliations:** 1 CAS Key Laboratory of Insect Developmental and Evolutionary Biology, CAS Center for Excellence in Molecular Plant Sciences, Institute of Plant Physiology and Ecology, Chinese Academy of Sciences, Shanghai, 200032 China; 2 CAS Center for Excellence in Biotic interactions, University of Chinese Academy of Sciences, Beijing, 100049 China; 3 Anhui Provincial Key Laboratory of Microbial Pest Control, Anhui Agricultural University, Hefei, 230031 China; 4 Biozeron Biotech Ltd., Shanghai, 201800 China; 5 School of Life Science and Technology, ShanghaiTech University, Shanghai, 201210 China

**Keywords:** Fungal ecology, Population dynamics

## Abstract

Entomopathogenic fungi are one of the key regulators of insect populations in nature. Some species such as *Beauveria bassiana* with a wide host range have been developed as promising alternatives to chemical insecticides for the biocontrol of insect pests. However, the long-term persistence of the released strains, the effect on non-target hosts and local fungal populations remains elusive, but they are considerable concerns with respect to environmental safety. Here we report the temporal features of the *Beauveria* population genomics and evolution over 20 years after releasing exotic strains to control pine caterpillar pests. We found that the isolates within the biocontrol site were mostly of clonal origins. The released strains could persist in the environment for a long time but with low recovery rates. Similar to the reoccurrence of host jumping by local isolates, the infection of non-target insects by the released strains was evident to endemically occur in association with host seasonality. No obvious dilution effect on local population structure was evident by the releases. However, the population was largely replaced by genetically divergent isolates once per decade but evolved with a pattern of balancing selection and towards expansion through adaptation, non-random outcrossing and isolate migration. This study not only unveils the real-time features of entomopathogenic fungal population genomics and evolution but also provides added values to alleviate the concerns of environmental safety regarding the biocontrol application of mycoinsecticides.

## Introduction

Fungal insect pathogens are essential regulators of host population density in nature [1, 2]. As effective alternatives to chemical insecticides, different species of *Beauveria* and *Metarhizium* have been developed as promising biocontrol agents (BCAs) against a wide range of agriculture and forestry insect pests [3, 4]. After field applications, investigating the fate of biocontrol releases is of great importance for both applied and basic research interests. Early studies of a few classical releases of different Entomophthoralean fungi revealed that either the long-term persistence of an Australian strain of the grasshopper pathogen *Entomophaga grylli* in North America was questionable [5] or it might have taken decades for the establishment of a Japanese species of *Entomophaga maimaiga* in North America to suppress the population of gypsy moth [6]. In contrast to these classical introductions, ascomycete fungal BCAs are conventionally used as mycoinsecticides. The released isolates of *Beauveria bassiana* could be recovered a few years after the biocontrol applications [7]. It has also been found that a released isolate of *Metarhizium robertsii* became adapted to local environments with mutations within 4 years [8]. The long-term persistence and environmental effects of mycoinsecticides applications have yet to be elucidated.

Two different scenarios, i.e., the trench warfare (the evolution of balancing selection) and arms race (the effect of selective sweeps), have been recognized for parasite-host coevolution [9]. In managed agricultural and forest ecosystems, the model of arms race rather than trench warfare is widely accepted for pathogen–plant interactions [10, 11]. In particular, the introduction of exotic strains could dramatically drive population shift of the rust pathogen *Puccinia striiformis* f. sp. *tritici* [12]. Insect pathogenic *Beauveria* and *Metarhizium* species are closely related to plant pathogens and endophytes [3, 13]. Except for the finding of spatial differentiation in genetic structure between populations [1, 14], the evolutionary pattern of fungus–insect interactions is unclear. Especially, the effect of biocontrol introductions on the evolution of local population structure remains a terra incognita.

In this study, we sequenced and analyzed the genomes of 277 *B. bassiana* isolates including those periodically collected from the same biocontrol-release site 20 years after the applications and the isolates sampled throughout other regions of China. We investigated the fate and consequence of the biocontrol applications. In particular, it remains obscure whether the released strains could persist in the released site for a long time to infect the non-target insects and whether there were dilution, replacement and/or recombination effects of biocontrol introductions on local populations. Regarding the dominant arms-race model of fungus–plant interactions in managed ecosystems, we asked if this was the case for our system as well? In addition, we determined the population demography by examining the possibility of isolate migration and gene flow.

## Results and discussion

### Isolate collection and genome sequencing

**Fig. 1:**
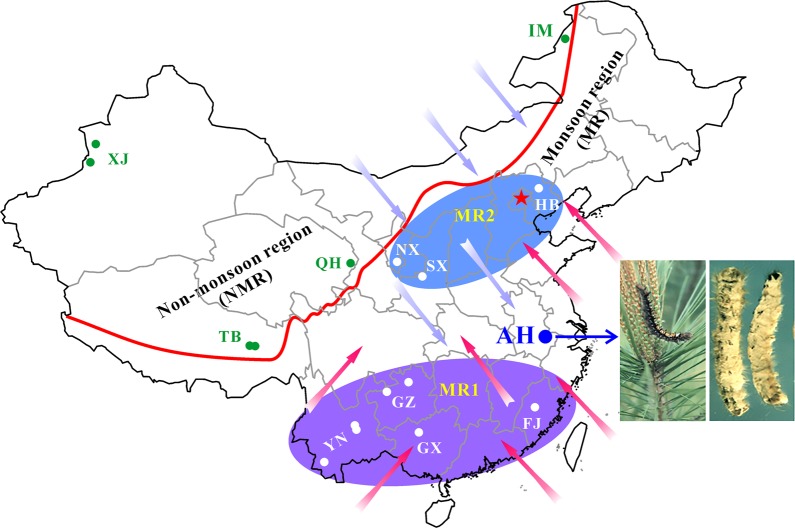
The sketch map of the isolate collection sites. The isolates of *Beauveria bassiana* were collected from the biocontrol site in the Anhui (AH) province and other places across the monsoon (MR) and non-monsoon (NMR) regions in China from 1997 to 2017 (Table S1). A blue arrow points to the infestation of the pine caterpillar and the mycosed cadavers after the application of mycoinsecticide at Magushan Forest Farm, AH province. Pink arrows show the wet/rainy phase of monsoon occurring in the later spring and summer seasons, and purple arrows indicate the dry phase of monsoon occurring in the autumn and winter seasons. The red line separates the NMR and MR regions by the mountain ranges in China. Provinces: XJ Xin-Jiang, TB Tibet, QH Qing-Hai, IM Inner Mongolia, HB He-Bai, NX Ning-Xia, SX Shan-Xi, YN Yuan-Nan, GZ Gui-Zhou, GX Guang-Xi and FJ Fu-Jian. The provinces in the south and north of AH within the monsoon regions are labelled as MR1 and MR2, respectively.

For spatial–temporal population genomic analyses, the collections of *B. bassiana* isolates were carried out from either the same biocontrol site multiple times over 20 years or one-time collections at different sites away from the biocontrol location. First, to investigate the fate and effect of biocontrol releases on local populations of *B. bassiana*, we performed multiple collections of mycosed insect cadavers from the Magushan pine forest farm at three intervals, i.e., 1997–1999, 2007 and 2017 (respectively termed the 1997, 2007 and 2017 populations for simplicity). Two strains of *B. bassiana* Bb17 and Bb13 were once used to control the pine caterpillars (*Dendrolimus punctatus*) at this farmland located in Anhui (AH) province, Southeast China, in 1995–1997 [7]. Over 20 years, the farmland vegetation has substantially increased (Fig. S1A, B). Second, to examine the possibility of gene flow with other populations, field collections were also conducted at other sites across the monsoon region (MR including those from regions south (termed MR1) and north (MR2) of the AH site) and non-monsoon region (NMR) of China (Fig. 1). After culture purification and isolate identification, we selected 270 isolates of *B. bassiana* with clearly identified insect hosts (at least to the order level) for whole-genome sequencing. These include two released strains (Bb13 and Bb17), 152 isolates (62, 47 and 43 for the 1997, 2007 and 2017 populations, respectively) collected from the biocontrol farmland and 116 isolates from additional places throughout China (Table S1). For these isolates, six orders of original insect hosts were identified mostly including the Coleopteran beetles (44%) followed by the Hemipteran (19%) and Lepidopteran (18%) insects (Fig. S1C). These three orders of insect hosts were also highly infected by the isolates collected from the AH population at different times (Fig. S1D). The data confirmed therefore that *B. bassiana* can infect a wide range of insect hosts in the fields [3, 15].

We performed genome re-sequencing for the selected isolates on an Illumina platform with an average of 70× coverage for each isolate (Table S2). Seven strains of *B. bassiana* with available genome information were also included in this study (Table S3). Reads were mapped to the reference strain ARSEF 8028, which has been assembled to the chromosomal level [16]. A total of 1,027,822 biallelic SNPs of high quality across the genome were subjected to further analyses (Fig. S2A).

### Clonal reproduction

The sexual life cycle of *B. bassiana* is still cryptic and rarely occurs in nature [17], and the fungus has a heterothallic nature that its haploid only contains a single mating type (MAT), i.e., either MAT1-1 or MAT1-2 [16, 18]. Based on the assemblies, BlastN survey indicated that there were 133 MAT1-1 and 136 MAT1-2 isolates (i.e. close to a 1:1 ratio), whereas eight isolates were unexpectedly identified to contain both MAT loci (putative heterokaryons) from these random collections (Table S4). An MAT ratio close to 1:1 was also observed for the AH population (52% MAT1-1 vs 48% MAT1-2), which is in contrast to a MAT1-1-biased (65%) population of *B. bassiana* collected from Denmark [15] and the population sampled from Cuba with a highly skewed distribution of MAT1-1 type isolates (MAT1-1:MAT1-2 = 146:4) [19]. The data suggest that the AH population might have a relatively higher potential for recombination compared with the European and South American populations.

**Fig. 2:**
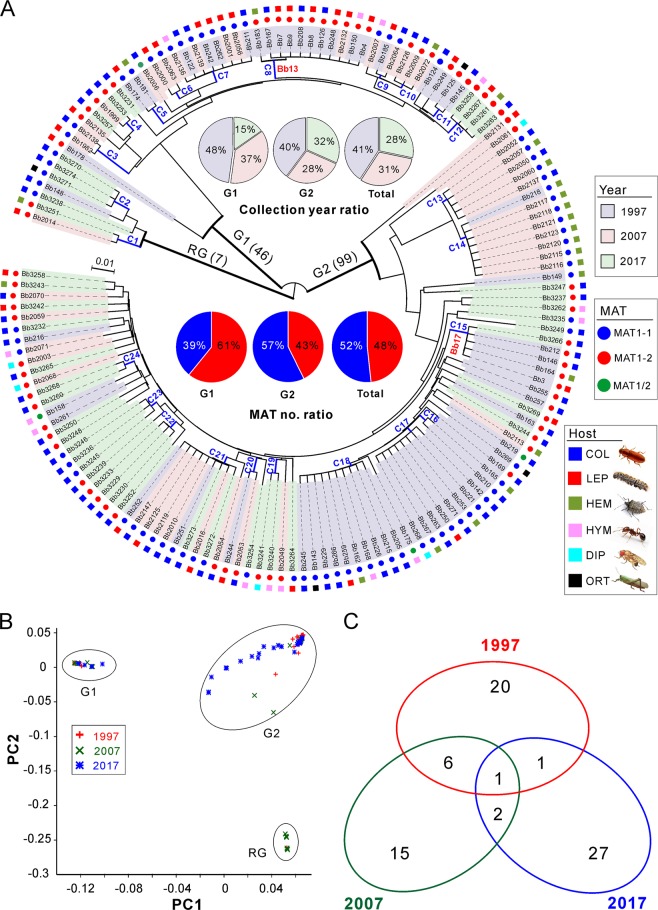
Relationship among the isolates collected at the biocontrol site over 20 years. **a** Phylogenetic analysis. The neighbour-joining tree is generated based on the average pairwise genetic distance of all biallelic SNPs. Isolates were separated into three main clustering groups, i.e., the root group (RG), G1 and G2 groups. The isolates shadowed in the different colours represent the respective collection year. The mating type (MAT) and original insect host of each isolate are indicated in different coloured circles and squares. C1–C24 are the clonal groups that clustered isolates with ANI values > 99.8%. The abbreviations of insect host orders are: COL Coleoptera, LEP Lepidoptera, HEM Hemiptera, HYM Hymenoptera, DIP Diptera and ORT Orthoptera. **b** Principal component analysis (PCA) of isolates based on all biallelic SNPs. The first two components are presented. **c** Venn diagram analysis of the clonal and divergent isolates collected in different years.

We next examined the genetic relationships of the 152 isolates collected from the AH biocontrol site. By using the obtained SNPs, both the phylogenomic tree and principal component analysis (PCA) consistently resulted in three well-separated lineages (Fig. 2a, b). Among these lineages, two main groups containing the majority isolates (termed as G1, including 46 isolates; and G2, including 99 isolates) are rooted by a basal lineage (including 7 isolates). Overall, the grouping was independent of isolate sampling date, MAT and host origin, which is similar to a previous observation [15]. We then calculated the average nucleotide identity (ANI) matrix [20] for these isolates. Taken together with the MAT genotype of each isolate (Fig. 2a), we found that the separated subclades could be divided from each other at a cut-off value of ANI > 99.8%. We termed these subclades as clonal lineages. Thus, 72 divergent lineages/genotypes were obtained including 24 clonal groups containing more than two isolates (Fig. 2a and Table S5), a pattern of multiple lineage coexistence like other fungal parasites [21, 22].

### Recovery of the released strains and evidence of host jumping

It has long been concerned regarding the perturbation of local populations after releasing the exotic strain of mycoinsecticides [23]. Within the AH biocontrol site, two highly virulent strains (Bb13 and Bb17) were selected by companies and applied for the control of Masson’s caterpillars [7]. Based on the estimate of ANI indices and isolate MAT genotypes, the released strain Bb13 was found to be closely related to the C8 clonal group (G1-lineage, 11 isolates; Table S6), while Bb17 was related to the G2-lieage C15 group (5 isolates; Table S7). Even both strains belonging to the MAT1-2 genotype (Table S1), these two strains are genetically different (ANI = 96.1%) from each other. Unsurprisingly, most of the strains were recovered not long after the releases (10/11 for Bb13, and 4/5 for Bb17). However, to complement our previous study [7], we found that the released strains could persist in the environment for a long time (e.g. the Bb13-like Bb2132 was recovered in 2007, while the Bb17-like Bb3266 was collected in 2017) and a few previously misidentified isolates were clarified. The low recovery rates of Bb13 (7.2%) and Bb17 (3.3%) imply that the released strains might play a marginal role in re-structuring local population. This finding thus alleviates the safety concerns of the dilution effect on local population after application of fungal BCAs [23]. Not unexpectedly, varied levels of mutations were detected between the released and recovered isolates and between clonal isolates collected in different years (Fig. S2B).

Host jumping of plant pathogens can lead to devastating threats to agricultures [24, 25]. Likewise, the effect of biocontrol releases on non-target hosts is one of the long-standing concerns of BCA environmental safety [23]. We found that many clonal isolates were isolated from two to five orders of insects and that the host-jumping events were also evident from the recovered strains (Fig. 2a). For example, ten strains of the C18 group were isolated from five orders of insects in association with host seasonality (Tables S5). In addition to infecting the target pine caterpillars, the recovered strains of Bb13 were found to able to kill coleopteran beetles (Bb126, Bb167 and Bb208), hemipteran bugs (Bb183) and hymenopteran wasps (Bb150). Likewise, Bb17-like strains could also infect the beetles (Bb146, Bb212 and Bb3266) and bugs (Bb164) (Fig. 2a and Table S1). In contrast to agricultural pathogenic fungi [10], this kind of frequent host jumping may relax the selection pressure on insect pathogens.

Virulence variation has been frequently observed between different strains of *B. bassiana* [26]. To verify the isolate ability to infect different insects, two released strains and three additional isolates from different hosts (Table S8) were used for insect bioassays against three insect species. The results confirmed that the released strains could kill non-target insects, such as the fruit-fly and mealworm, and that the isolates originally isolated from one insect order could kill the other order insects, the clear support of isolate host jumping. Nevertheless, considerable differences are present between different strains against the same insect or for the same isolate against different insect species (Fig. S3 and Table S8), indicative of a certain degree of isolate host preference. Taken together with the fact that fungal infection is host-density dependent [27], the similar infection patterns between the released strains and field isolates could help alleviate the safety concern regarding the non-target infection by biocontrol introduction of exotic strains of *B. bassiana*.

### Population replacement and evolution over 20 years

**Fig. 3:**
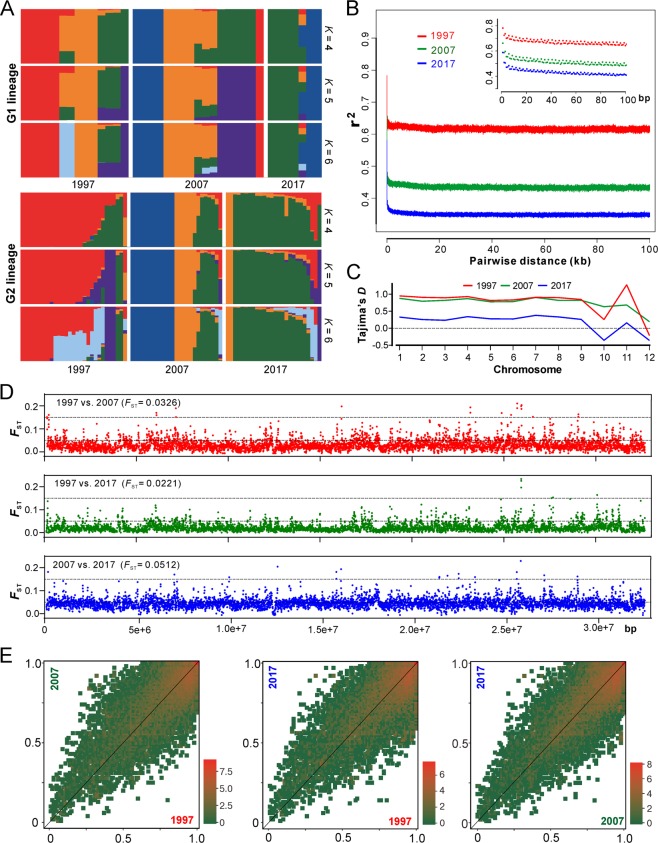
Comparative analysis of population structure. **a** Ancestry components of two major lineages of AH isolates (as shown in Fig. 2a). **b** Decay of linkage disequilibrium (LD, expressed in terms of correlation coefficient, *r*^2^) as a function of distance averaged over 100 kb for the three populations. Inset shows the declines in LD over the first 100 bp. **c** Estimation of Tajima’s *D* of the three populations. Tajima’s *D* was independently calculated within 5-kb sliding windows across the genome. The median value of each chromosome is shown. **d** Genome-wide analysis of genetic divergence between populations. The value of *F*_ST_ was calculated in 5-kb windows, and the median values shown in parentheses were estimated between two populations. The cut-off lines shown in each panel represent the Wright’s threshold (*F*_ST_ = 0.05–0.15) for moderate differentiation. **e** Derived allele frequency spectra between pairwise populations. The colour of each slot indicates the frequency of each derived allele pair. The scale values along each panel are the exponent of order 2.

In managed ecosystems, the complete change of host genotypes (e.g. the introduction of disease-resistant cultivar) will drive the replacement of parasite populations [10]. For AH population, we found that most of the clonal isolates/lineages were specific to a single collection time (Fig. 2c), which reflected a pattern of isolate replacement/recurrence every 10 years. Only one clone (C21, nine isolates) was evidently collected throughout the sampling periods (Table S5). We further analyzed the ancestry of the two major G1 and G2 lineages, and the results support the observations that a substantial proportion of the isolates within the AH population was replaced every 10 years and especially after 20 years (Fig. 3a). Since the population has not been dominated by a single genotypic isolates (including the released strains), this observation ruled out an arms-race model of *Beauveria* evolution, which is in contrast to the observations for plant pathogens [9, 11, 28].

To further investigate the population evolutionary scenario, we calculated the decay of linkage disequilibrium (LD) for three populations to determine the non-random association of SNP alleles at different loci [22]. We found that LD decayed rapidly in each collection period but never decreased below 50%, which is different from the estimation of yeast populations [22, 29]. Such a LD decay pattern suggests that a limited number of historical recombination events might have occurred in the population [30]. Interestingly, LD decay dropped more substantially along the collection dates, i.e., the more recent the sampling date, the faster the LD decay (Fig. 3b). This finding suggests that either the selection was relaxed or the population underwent expansion during the 20-year evolution.

Our estimation of the genome-wide Tajima’s *D* indices revealed that the chromosome-level values were mostly positive, especially for the 1997 and 2007 populations, while dropped towards zero for the more recent 2017 population (Figs. 3d and S4). Genome-wide Tajima’s *D* is generally sensitive to demographic events and positive values indicate a balancing selection [31, 32]. Thus, consistent with the LD decay analysis, the data support such an evolutionary scenario that the *Beauveria* population would have evolved under balancing selection and towards expansion over the past 20 years. Considering the relatively short evolutionary period, it is not surprising to find that the genetic differentiation was basically marginal between decades-long populations that were revealed by both pairwise *F*_ST_ analyses (Fig. 3e) and the covariant spectra of the derived allele frequency (Fig. 3f). Instead, substantial genetic divergence were observed between G1 and G2 lineages (median *F*_ST_ = 0.8805) but not between MAT1-1 and MAT1-2 genotypes (median *F*_ST_ = 0.0419) (Fig. S5). This suggests that there has been little genetic admixture between G1 and G2 lineages.

Due to host resistance, the infection-related genes of plant pathogens are under strong selection pressure [10]. The development of insect resistance to entomopathogenic fungi has also been suggested [33]. We next performed the composite likelihood ratio (CLR) test to identify selective sweeps across the whole genome in each temporal population. By scanning CLR-based signatures in each 1-kb genomic window [34], the overall distribution pattern revealed that the selection signatures, although varied between chromosomes, were highly consistent across the temporal populations (Fig. S6). Similar to the previous findings in fungal plant pathogens [10, 35], we found that the genomic regions with the highest selective signatures (5% quantile) are enriched with transposable elements (TEs), as 1558 out of the 2678 windows (58%) overlapped with a potential TE region. The genes encoded in these regions were also identified. Similar to the finding in plant pathogens [28], we found that the putative effector genes involved in fungus–host interactions were overrepresented in these TE-rich regions, i.e., 8.65% (18 out of 208 genes encoded in the top 5% CLR regions), 6.91% (15/217) and 7.94% (17/214) in the 1997, 2007 and 2017 populations, respectively. Otherwise, the hypothetical proteins without conserved domains are encoded and enriched in these regions for each population (Table S9), confirming the quickly evolved genomic regions under selection. Thus, similar to the findings in plant pathogens including oomycetes [28, 35], the *Beauveria* genome also contains mosaic structures with TE-rich compartments containing putative virulence-related genes that are under strong-adaptive selection that may contribute to the periodical population replacement and expansion observed above.

### Genetic recombination and sexuality induction

**Fig. 4:**
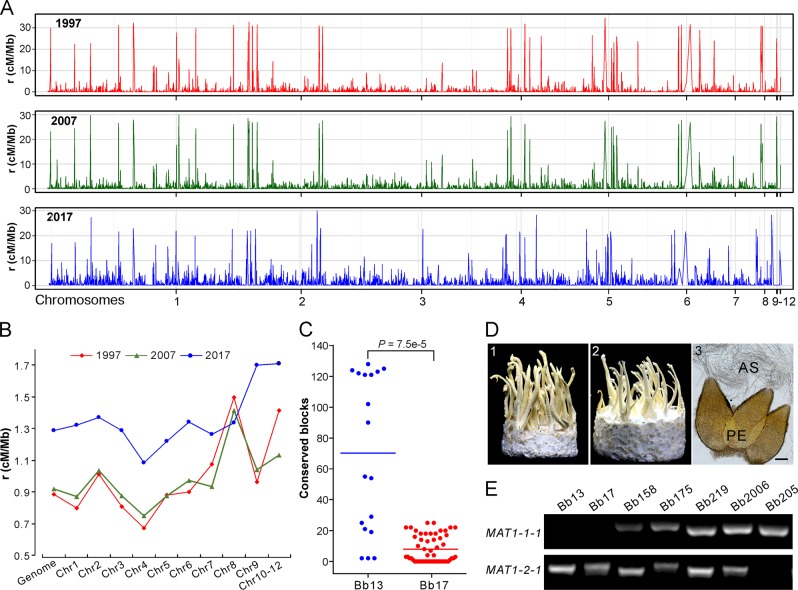
Genetic recombination analysis. **a** Variation in recombination rates (RRs) across chromosomes in three populations. **b** Comparative estimation of RRs along the genome for three populations. The median value of each chromosome was used for plotting each population. **c** Identification of potential recombinant genomic blocks, which were determined by >1000 consecutive and shared SNPs between the released strains and local opposite mating-type isolates. Each dot represents the number of the conserved SNP blocks present in the MAT1-1 isolates of G1 or G2 lineages shared with the released strain Bb13 or Bb17. The lines in the middle show the median values. **d** Sexuality induction between the opposite mating-type isolates. Panels 1 and 2 represent the phenotypes of crossings: Bb175 MAT1-1 × MAT1-2, and Bb166 (MAT1-1) × Bb175 (MAT1-2); Panel 3, microscopic observation of the sexual perithecia (PE) and asci (AS) produced on the inducible fruiting bodies. Bar, 50 μm. **e** PCR verification of the karyotype of the selected isolates. The presence or the absence of the fragment indicates isolates belonging to the MAT1-1 and or MAT1-2 genotype.

Both clonality and sexuality are likely involved in the reproduction of all fungal species [36], and sexual cycle plays a more substantial role than clonal reproduction in altering genetic structure and speeding adaptation of fungal populations [37]. Despite the overall clonal feature of the *Beauveria* population, the fast LD decay of each population manifested above suggests the occurrence of outcrossing between the opposite MAT isolates. We therefore estimated the genome-wide recombination rates (RRs) using an unbiased and linear-correction algorithm [38]. As a result, recombination signatures were evident along the genome for each population (Fig. 4a, b). Overall, consistent with the pattern of LD decay (Fig. 3b), the most recent 2017 population presented a higher RR (average *ρ* = 1.29 cM/Mb) than the 2007 (average *ρ* = 0.92 cM/Mb) and 1997 (average *ρ* = 0.89 cM/Mb) population. The data suggest therefore that the *Beauveria* population evolved towards the increased potential of sexual reproduction along the sampling time, which might have additionally contributed to isolate adaptation and population replacement.

Based on the isolates’ phylogenetic relationships (Fig. 2a), we analyzed potential recombination signatures between the released strains and sampled isolates. Genome-wide scanning (see “Materials and methods”) identified the conserved blocks between Bb13- and G1-lineage isolates (Fig. S7) and between Bb17- and G2-lineage strains (Fig. S8). It is not surprising to find the highly conserved relationships between Bb13/Bb17 and their clonal relatives. However, the number of conserved blocks was significantly different between released strains and their relatives, i.e., there were significantly (*t*-test, *P* = 7.5e–5) more conserved blocks between Bb13 and its opposite-MAT isolates than between Bb17 and its putative partners (Fig. 4c). This finding suggests that Bb17 was more likely to recombine with local isolates than Bb13.

We further experimentally examined the induction of sexual fruiting-body formation on an artificial medium between different isolates with the opposite MAT (Table S10). Initial tests with those eight strains containing both MAT1-1 and MAT1-2 loci (Table S1) demonstrated that only the Bb175 strain could produce sexual fruiting bodies (FBs), perithecia and asci (Fig. 4d). We then performed single-spore isolations and confirmed the presence of both MAT1-1 and MAT1-2 in Bb175 and the karyotypes of other isolates (Fig. 4e). Interestingly, the MAT1-2 isolate of Bb175 mated with and produced sexual FBs with a few other MAT1-1 isolates (4/29 of pairing tests). Among the pairing tests, sexual FBs were also formed between Bb13- and the G1-lineage MAT1-1 isolate (1/13 of pairing tests) and between Bb17- and the G2-lineage MAT1-1 strains (2/18 of pairing tests; Table S10). Thus, the data imply that outcrossing might be substantially deviated from random mating in *Beauveria* populations in natura, which may be due to the high frequency of vegetative incompatibility between different isolates of *B. bassiana* [39].

### Gene flow between geographic populations

**Fig. 5:**
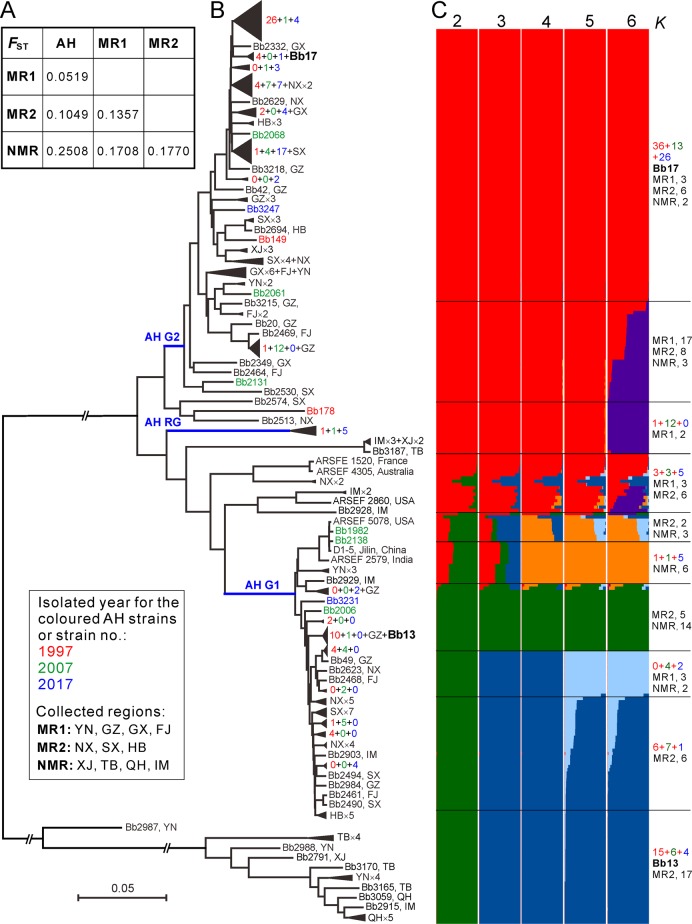
Divergence and gene flow between geographic populations. **a** Median genetic differentiation (*F*_ST_) between the pairwise populations. AH, MR1, MR2 and NMR populations are as shown in Fig. 1. **b** Phylogenetic relationships of all isolates collected from different geographic origins. The Neighbour-joining tree was obtained based on the average pairwise genetic distance of all biallelic SNPs. For simplicity, lineage grouping is determined at a cut-off value of ANI > 99%. The blue branch lines represent the associations with the AH population phylogenies obtained in Fig. 2a. Two released strains Bb13 and Bb17 are highlighted in bold. **c** Genetic relationships among fungal isolates based on the estimation of individual ancestry. The bar plots in each line show the ancestry proportion over a range of presumed ancestry number, *K* = 2–6. Abbreviations for geographic origins are as shown in the caption of Fig. 1.

Long-distance migrations have been frequently evident for fungal plant pathogens through aerial dispersal and human commercial activities [40]. In addition to those known routes, the propagules of insect pathogens can also hitchhike along with migrating insect hosts [41]. To determine *Beauveria* demography, we analyzed the genetic relationships of *Beauveria* isolates between AH and other geographic populations. We found that the AH population has the least genetic differentiation with the MR1-originated isolates followed by the MR2 and NMR populations (Figs. 5a and S9), which is generally consistent with the Chinese monsoon climate features (Fig. 1). Again, the phylogenetic tree recovered the two main AH G1- and G2-like lineages comprising most isolates sampled from either the MR1 or MR2 regions and leaving most NMR isolates distantly separated (Fig. 5b).

We performed ancestry estimation and the results confirmed that AH isolates demonstrated clear signs of admixture with those strains collected from either the MR1 or MR2 regions (Fig. 5c). In total, 9 MR1, 8 MR2 and 2 NMR isolates had a genetic admixture with the AH G1-lineage, while 27 MR1, 17 MR2 and 3 NMR strains were closely related to the G2-lineage. Taken together, the data revealed that frequent gene flow might have occurred between AH and other populations as well as between different well-separated populations, which might have played additional roles in population replacement and expansion of *B. bassiana*.

## Conclusion

We report the ecological and evolutionary scenario through time of a population of *B. bassiana* from a forest farmland after releasing exotic strains to control caterpillar pests for 20 years. We found that the *Beauveria* population is largely clonal in nature and the released strains could persist in the field for a long time (20 years) but with low recovery rates (<8% after 20 years) that can rule out the dilution-effect concerns of biocontrol introductions, which is in contrast to effect of the plant fungal pathogen introduction [12]. Isolate jumping to different insect hosts frequently occurred in the field and the released strains could similarly infect non-target insects, however, which may not pose any substantial threats to non-target insect population safety. In contrast to the widely reported arms-race coevolution between fungal pathogens and plant hosts in managed ecosystems [10, 11], we found that the *Beauveria* population evolved under balancing selection (i.e. a trench-warfare scenario) even with substantial population replacements once a decade time through the joint effects of adaptation, non-random outcrossing and isolate migrations. In particular, the wide host range of *B. bassiana* may help relax the selection pressure on fungal population structure. Overall, in addition to unveiling the unique and real-time features of fungal population genomics and evolution, our data can greatly alleviate the safety concerns of the biocontrol application of mycoinsecticides. Considering that *Beauveria* species are also soil dwelling [1], future efforts are still required to expand the field sampling depth for the population study. Population evolution of the other species of entomopathogens also remains to be investigated, especially for the biocontrol species such as *Metarhizium acridum* with a narrow host range.

## Materials and methods

### Isolate collections

Sampling location was at the Magushan Forest Farm (30.57° N; 118.55° E) in AH province, Southeast China. It is a planted pine forest (ca. 20,000 ha) with the frequent infestation of the Masson’s pine caterpillar (*D. punctatus*). The formulations of *B. bassiana* strains Bb17 (used late 1995 and 1996) and Bb13 (applied late 1997) produced by a company were once applied subsequently to control the caterpillar pests [7]. Both strains were originally isolated from the pine caterpillars. Field collections were conducted in 1997–1999, 2007 and 2017 at different seasons (Table S1). The insect cadavers were collected and the isolates were purified, morphologically identified, stored in −80 °C and catalogued at the Research Centre of Entomopathogenic fungi (RCEF), Anhui Agricultural University, China. The infected insect hosts were also identified at least to the order levels. To place the AH population in the context of around populations, large-scale collections were also performed at other 16 representative places located in the MR or NMR in China [42]. The cultures were purified, identified and catalogued at RCEF and those isolates with clearly identified hosts were also included in population genomics analysis (Table S1). Seven strains of *B. bassiana* with available genomic sequencing reads were also included in our population genomic analysis [16, 18]. These strains were originally isolated from different insect hosts and different countries, including Australia, Denmark, France, India and the USA (Table S2). In particular, the high-quality genome data of the strain ARSEF 8028 sequenced with 12 near-complete chromosomes were used as a reference [16].

### DNA extraction, library construction and Illumina sequencing

Each isolate was inoculated on the glass paper lined on the potato dextrose medium and the day 4 sample was collected for genomic DNA extraction using the DNA-EZ Reagents (Sangon Biotech, Shanghai). After quality test by agarose gel analysis, the quantified DNA samples (1 μg each) were used for paired-end library constructions (with an insert size of ~400 bp) by following the Illumina’s standard library preparation procedure. The quantified libraries were sequenced using an Illumina Hiseq X system to generate ~4 Gb raw data for each isolate. Draft de novo assembly of the read data was performed for each isolate using SOAPdenovo2 [43].

### Read mapping and SNP calling

The raw sequencing data were processed for base calling using the software CASAVA v1.8.2 (Illumina), and the quality control was conducted using the programme Trimmomatic with default parameters [44]. The high-quality reads were mapped to the reference genome sequence of the strain ARSEF 8028 using Burrows–Wheeler Aligner with the MEM module at default parameter sets (BWA, v0.7.4) [45]. The sequencing depth and coverage were calculated for each isolate based on the alignments and processed using custom perl scripts. SNP variants were identified using the best-practice pipeline of GATK toolkit (v3.8) [46]. To obtain the high-quality results for further analyses, the idiomorphic MAT locus was excluded and we only retained biallelic SNPs.

### Population genomic analysis

For phylogenetic analysis, the neighbour-joining tree was constructed using the PHYLIP software (v3.5) on the basis of a distance matrix. PCA of whole-genome SNPs was performed with the smartPCA programme in the EIGENSOFT package (v. 5.1). ANI was estimated between pairwise isolates using OrthoANI [20]. Population genetic structure and individual ancestry was estimated using FRAPPE [47]. The pre-defined clusters from *K* = 2 to *K* = 6 were run with 10,000 maximum interactions. LD was estimated for each chromosome in 500 bp windows using Haploview [48] with the parameters “-maxdistance 200 -dprime -minGeno 0.6 -minMAF 0.1”. Genome-wide LD decay was represented by the mean value of *r*^*2*^. Since LD is sensitive to the sample size, equal number of samples were randomly selected from each group. Population genetic statistics of Tajima’s *D*, and *F*_ST_ were calculated in 5 kb windows using the custom script as described [49]. The cut-off lines were drawn in figures based on the Wright’s threshold (*F*_ST_ = 0.05–0.15) for moderate differentiation [50]. The genome-wide selection signatures were identified using Sweepfinder2 [34], and CLRs were computed every 1 kb along each chromosome. All 1-kb genomic windows were sorted based on the CLR score and the highest 5% quantile was considered as loci under selection. Genes encoded in the selection regions were subjected to analysis for both functional enrichment and identifying putative fungal effectors using EffectorP [51]. Population RRs were estimated by scanning each chromosome with 10 kb sliding windows using the software package FastEPRR [38].

### Mating-type determination and sexuality induction

To determine the MAT of each isolate, the sequences of *MAT1-1-1* and *MAT1-2-1* obtained from the respective reference genomes (Table S2) were used for BLASTN analysis against the assembled genome sequence of each strain. In addition, PCR verifications were conducted using the primer pairs Mat1F (CAATTCTCCGTCATCTGCAA, synthesized by Sangon Biotech) and Mat1R (TTCTTCTGTGGCAAGGCTCT) for *MAT1-1-1* gene, and Mat2F (CAGACATACCATTGTGAAGCAG) and Mat2R (TGCTGCTCGAGGAGAGCTT) for *MAT1-2-1* gene. The sexual stage of *B. bassiana* has been identified as *Cordyceps bassiana* [17]. To determine the potential of sexuality between the opposite MAT isolates, formation of sexual FBs was induced on the unmilled brown rice containing silkworm pupal homogenates [52]. Microscopic examination of the formation of sexual perithecia and asci was performed for those opposite MAT isolates that could produce FBs.

### Estimation of genetic crossover between released strains and local isolates

To identify potential recombination events between the released strains (i.e. Bb13 and Bb17) and local isolates, we performed genome-wide scanning for the presence of conserved blocks between Bb13- and G1-lineage isolates and between Bb17- and G2-lineage isolates (Fig. 2a). We considered the long blocks containing continuously identical SNP alleles between two strains as potential segments that were exchanged between the genome of Bb13 or Bb17 and the target genome. As a stringent threshold, such crossover blocks were limited to local segments of at least 1500 continuous SNPs with identical genotypes, an equivalent of ∼5 kb resolution [53]. Both Bb13 and Bb17 are of MAT1-2 MAT that is unlikely to recombine with isolates of the same MAT, we therefore hypothesized such identical blocks to be originally conserved sequences across the MAT1-2 strains. These regions were excluded from the identified blocks being also present in the opposite MAT (i.e. MAT1-1) isolates based on the overlapping analysis. The number of the remained blocks shared between each MAT1-1 isolate and Bb13 or Bb17 was then estimated and implicated as the reminiscent of crossover between the released strain and local isolates.

### Insect bioassays

To verify the virulence difference between isolates, a few strains were selected for bioassays including the two released strains Bb13 and Bb17, Bb269, Bb3241 and ARSEF 8028 from different insect hosts (Table S1). The spores harvested from two-week old potato dextrose agar plates were used for immersion bioassays against the female adults of *Drosophila melanogaster* (representing the Dipteran order insect) and the wax moth (*Galleria mellonella*) larvae (representing the Lepidopteran order insect) using the same concentration of spore suspension (3 × 10^7^ spores ml^−1^); and mealworm larvae (*Tenebrio molitor*, representing the Colepteran order insect) using a spore suspension of 5 × 10^7^ spores ml^−1^. There are three replicates for each isolate per insect species and all experiments were repeated twice. The median lethal time (LT_50_) was estimated for each isolate and compared with each other using a Kaplan–Meier analysis [54].

## Supplementary information

Fig. S1

Fig. S2

Fig. S3

Fig. S4

Fig. S5

Fig. S6

Fig. S7

Fig. S8

Fig. S9

Table S1

Table S2

Table S3

Table S4

Table S5

Table S6

Table S7

Table S8

Table S9

Table S10

## Data Availability

The whole-genome sequence data in this study has been deposited at DDBJ/ENA/GenBank under the Bioproject ID PRJNA488964 and the Biosample IDs SAMN09946300–SAMN09946609.
