# Supplementary material for: Population genomics and evolution of a fungal pathogen after releasing exotic strains to control insect pests for 20 years
Source: ISME J. 2020 Feb 28;14(6):1422–34. doi: 10.1038/s41396-020-0620-8 (PMC7242398; doi:10.1038/s41396-020-0620-8)
Supplement: Supplementary file 1 — Fig. S1 [file 41396_2020_620_MOESM1_ESM.pdf]

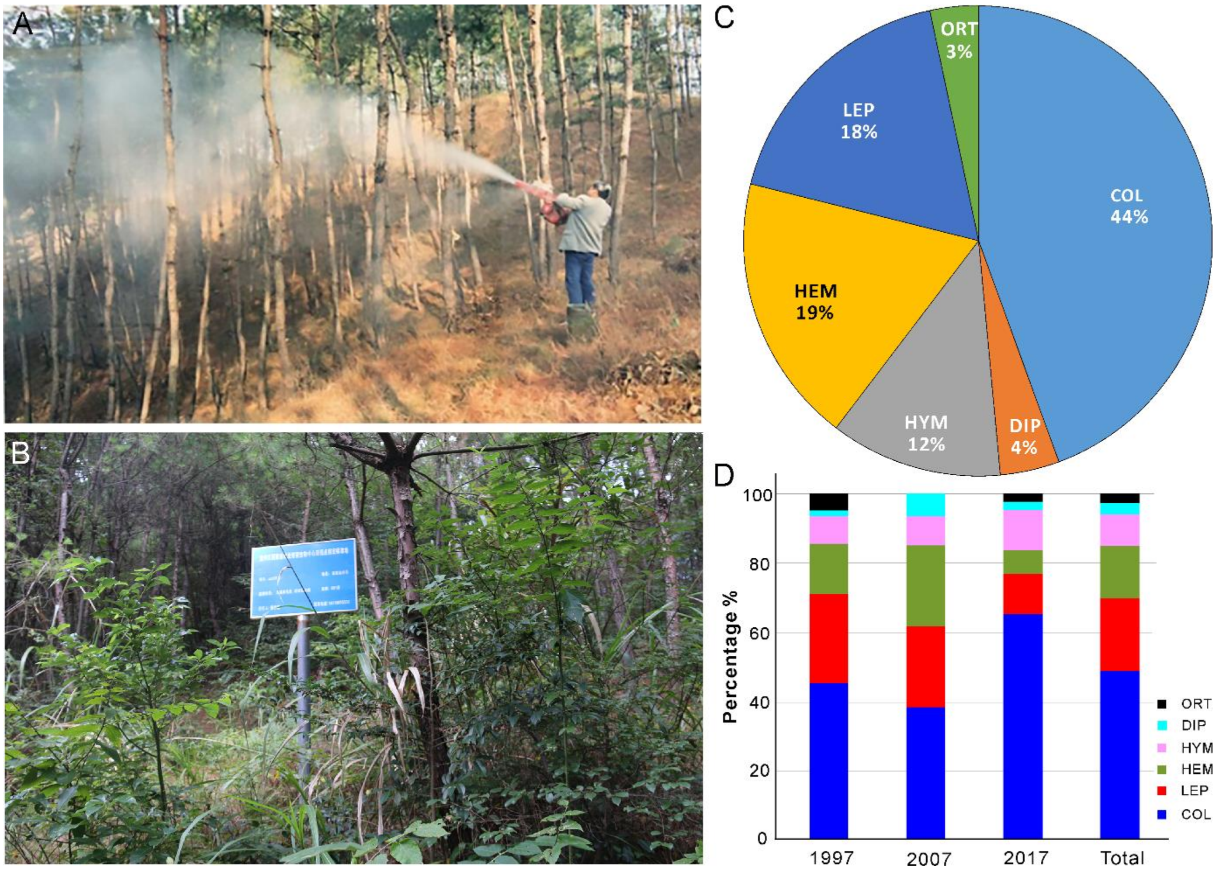

Fig. S1. Forest forage comparison and insect host estimation. (A) Field release of *Beauveria* spores formulated in oil at the Magushan pine forest farm in 1996. (B) The status of pine farmland vegetation taken in 2017. (C) Percentage estimation of the insect host orders originally infected by the 277 isolates of *B. bassiana* included in this study. (D) Insect order composition of the isolates collected from the AH biocontrol site. The abbreviations of insect host orders are: COL for Coleoptera; LEP, Lepidoptera; HEM, Hemiptera; HYM, Hymenoptera; DIP, Diptera; and ORT, Orthoptera.
