# Supplementary material for: Population genomics and evolution of a fungal pathogen after releasing exotic strains to control insect pests for 20 years
Source: ISME J. 2020 Feb 28;14(6):1422–34. doi: 10.1038/s41396-020-0620-8 (PMC7242398; doi:10.1038/s41396-020-0620-8)
Supplement: Supplementary file 2 — Fig. S2 [file 41396_2020_620_MOESM2_ESM.pdf]

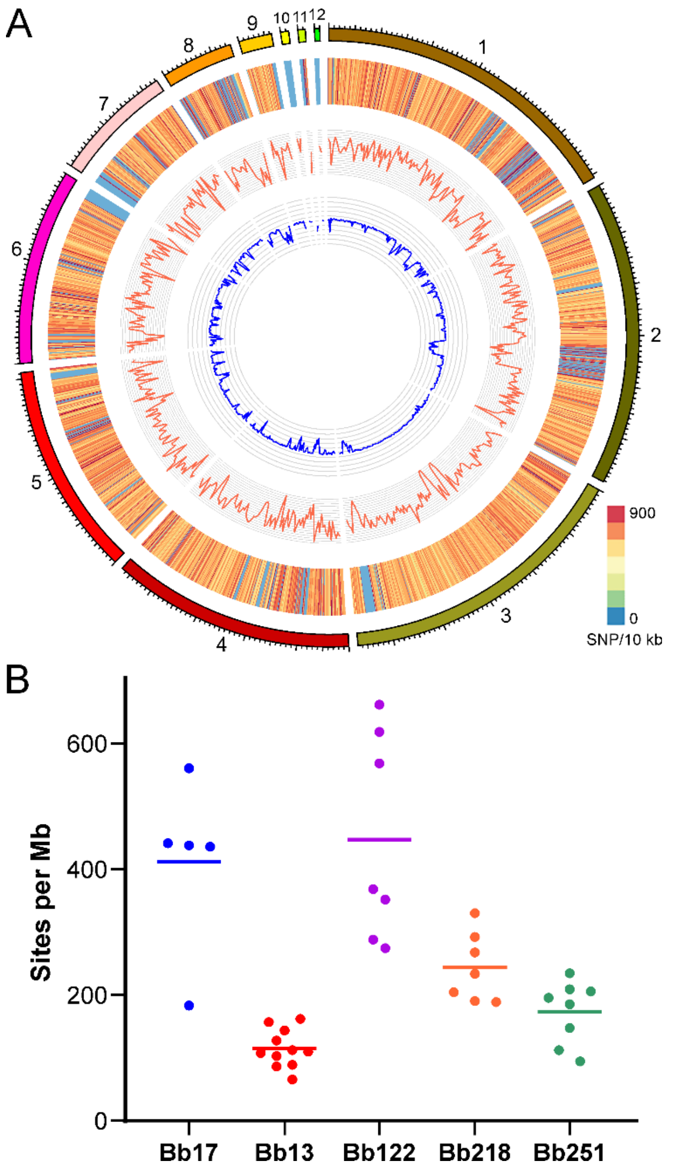

Fig. S2. Genome resequencing and mutation estimation. (A) Schematic summary of different characteristics estimated from the sequenced isolates. From inside to the outside: GC%; gene richness; SNP richness (lined to the scale shown at the right bottom) and the schematic chromosomes. (B) Estimation of genome-wide mutations between released and recovered strains and between different clonal isolates. The released strains Bb13/Bb17 and representative isolates Bb122 (C7 lineage), Bb218 (C14 lineage) and Bb251 (C17 lineage; Figure 2a) were used as individual reference for detection of the mutation sites present in the recovered or clonal isolates collected at different times, i.e. the clonal isolates collected at the single sampling date (e.g. C17 and C18 lineages) were not included in analysis due to (replicative) time concern.
