# Supplementary material for: Population genomics and evolution of a fungal pathogen after releasing exotic strains to control insect pests for 20 years
Source: ISME J. 2020 Feb 28;14(6):1422–34. doi: 10.1038/s41396-020-0620-8 (PMC7242398; doi:10.1038/s41396-020-0620-8)
Supplement: Supplementary file 3 — Fig. S3 [file 41396_2020_620_MOESM3_ESM.pdf]

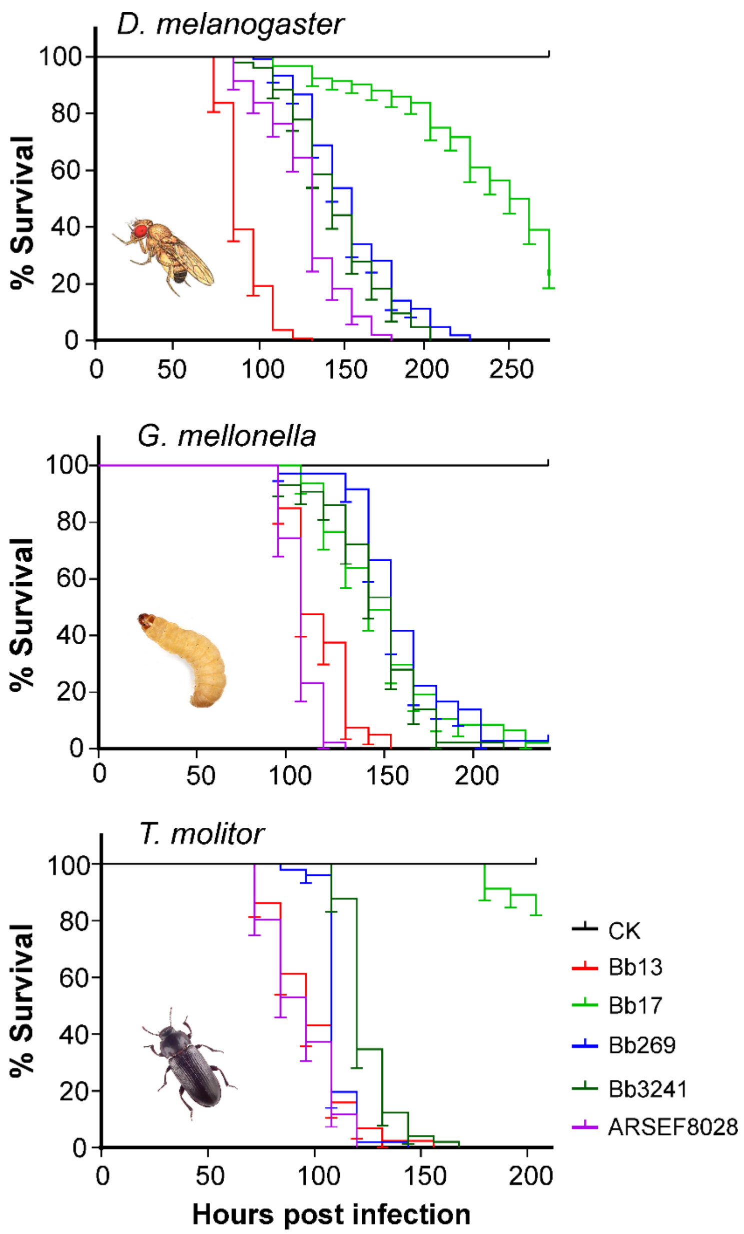

Fig. S3. Insect bioassays. Survival of the fruit fly *D. melanogaster*, *G. mellonella* and *T. molitor* after topical infection with the spores of different isolates. The background information for the selected isolates are shown in Table S8, representing the released strains (Bb13 and Bb17), Orthopteran host-origin isolate (Bb269), Dipteran host-origin isolate (Bb3241) and Hemipteran host-origin isolate (Bb8028).
