# Supplementary material for: Population genomics and evolution of a fungal pathogen after releasing exotic strains to control insect pests for 20 years
Source: ISME J. 2020 Feb 28;14(6):1422–34. doi: 10.1038/s41396-020-0620-8 (PMC7242398; doi:10.1038/s41396-020-0620-8)
Supplement: Supplementary file 4 — Fig. S4 [file 41396_2020_620_MOESM4_ESM.pdf]

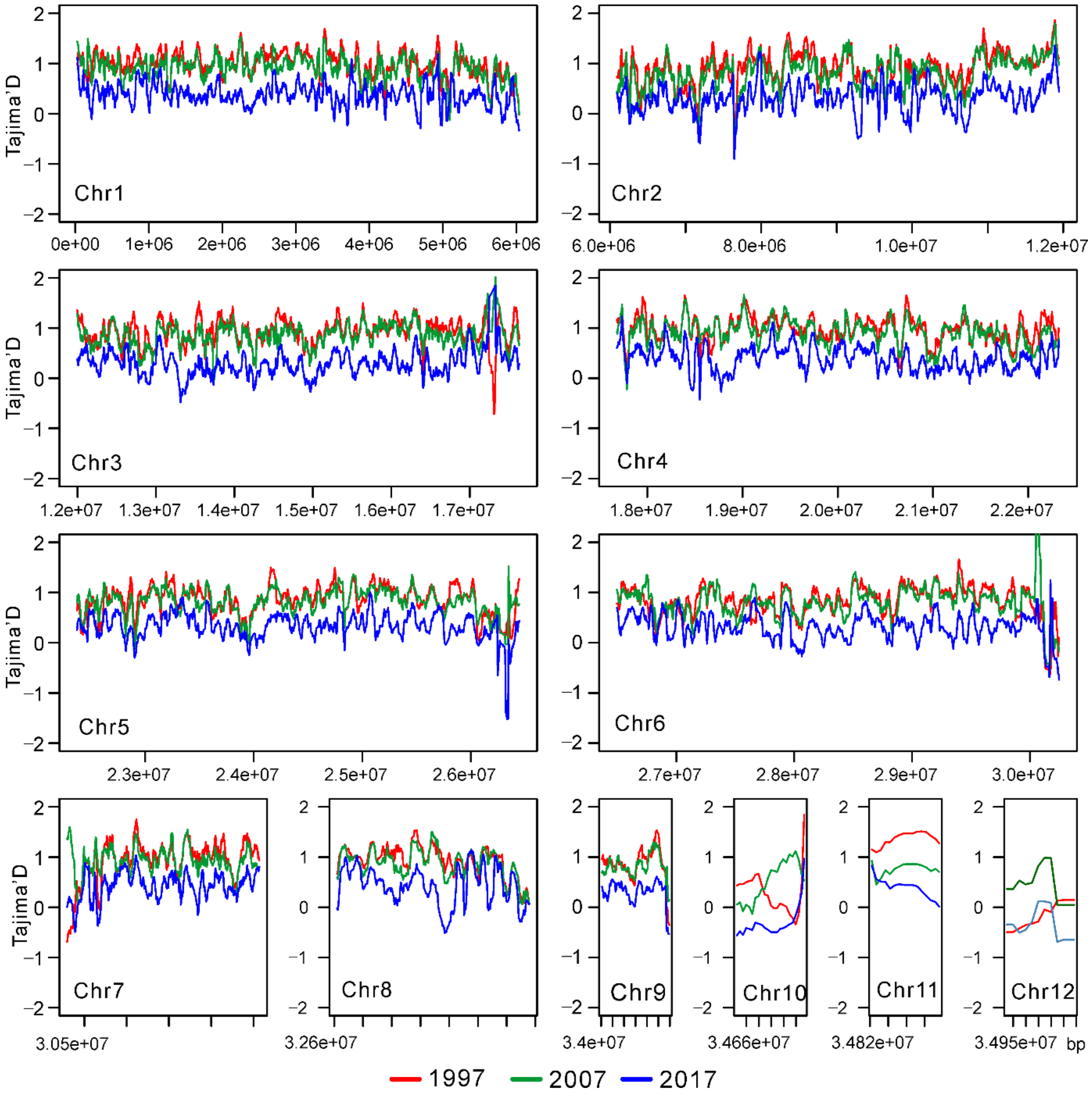

Fig. S4. Comparative estimation of genetic diversity at individual chromosome level between populations. The Tajima's *D* indices were estimated in 5 kb windows along individual chromosomes for the three populations of *B. bassiana*.
