# Supplementary material for: Population genomics and evolution of a fungal pathogen after releasing exotic strains to control insect pests for 20 years
Source: ISME J. 2020 Feb 28;14(6):1422–34. doi: 10.1038/s41396-020-0620-8 (PMC7242398; doi:10.1038/s41396-020-0620-8)
Supplement: Supplementary file 5 — Fig. S5 [file 41396_2020_620_MOESM5_ESM.pdf]

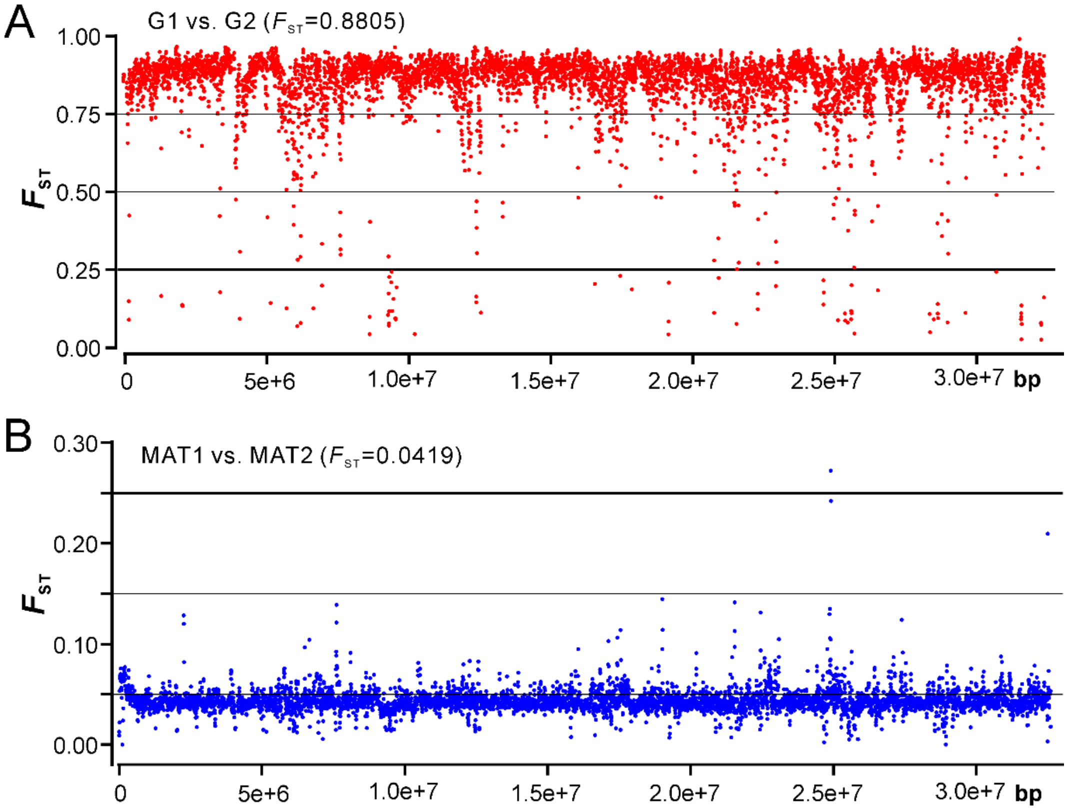

Fig. S5. Genetic divergence between phylogenetic lineages and mating-types. (A) Substantial genetic divergence between G1- and G2-lineage isolates obtained in Figure 2*A*. (B) Marginal genetic divergence between MAT1-1 (MAT1) and MAT1-2 (MAT2) isolates of *B. bassiana*. The value of F_{ST} was calculated across 5 kb windows.
