# Supplementary material for: Population genomics and evolution of a fungal pathogen after releasing exotic strains to control insect pests for 20 years
Source: ISME J. 2020 Feb 28;14(6):1422–34. doi: 10.1038/s41396-020-0620-8 (PMC7242398; doi:10.1038/s41396-020-0620-8)
Supplement: Supplementary file 6 — Fig. S6 [file 41396_2020_620_MOESM6_ESM.pdf]

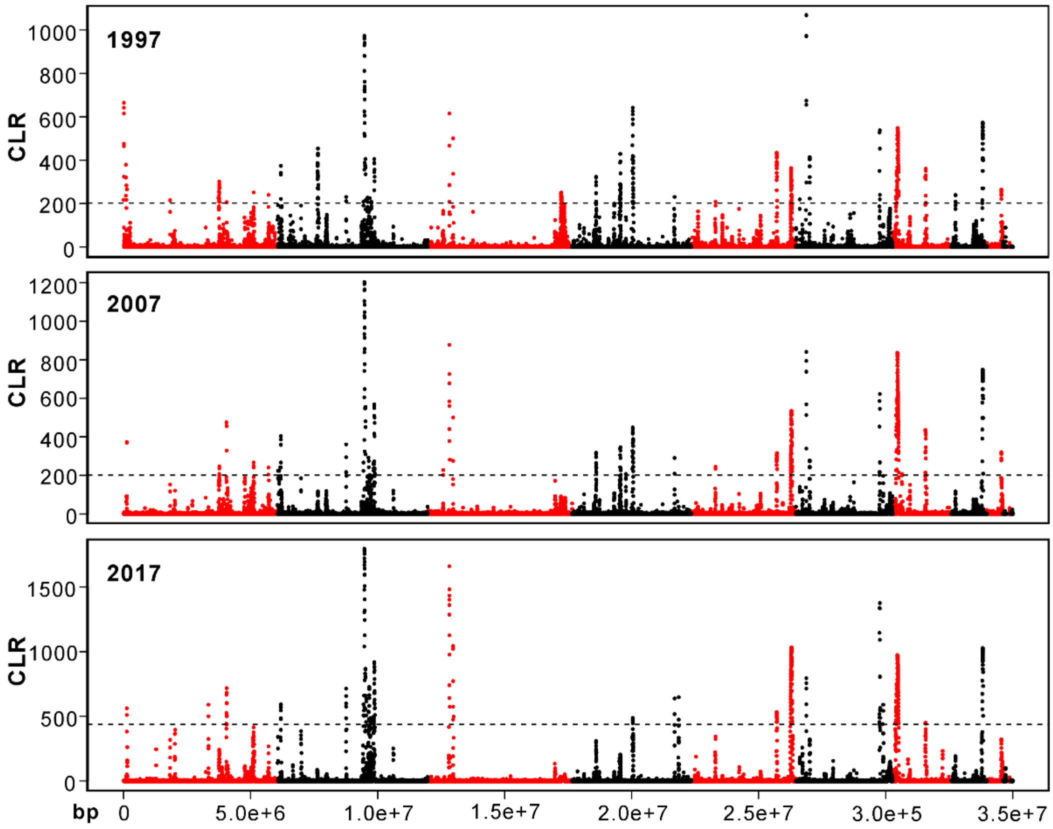

Fig. S6. Genome-wide scanning for regions under selection in each population. Estimation of the composite likelihood ratio (CLR) values across 1 kb windows for each population. The cutoff line in each panel shows the top 5% of CLR values for each population.
