# Supplementary material for: Population genomics and evolution of a fungal pathogen after releasing exotic strains to control insect pests for 20 years
Source: ISME J. 2020 Feb 28;14(6):1422–34. doi: 10.1038/s41396-020-0620-8 (PMC7242398; doi:10.1038/s41396-020-0620-8)
Supplement: Supplementary file 8 — Fig. S8 [file 41396_2020_620_MOESM8_ESM.pdf]

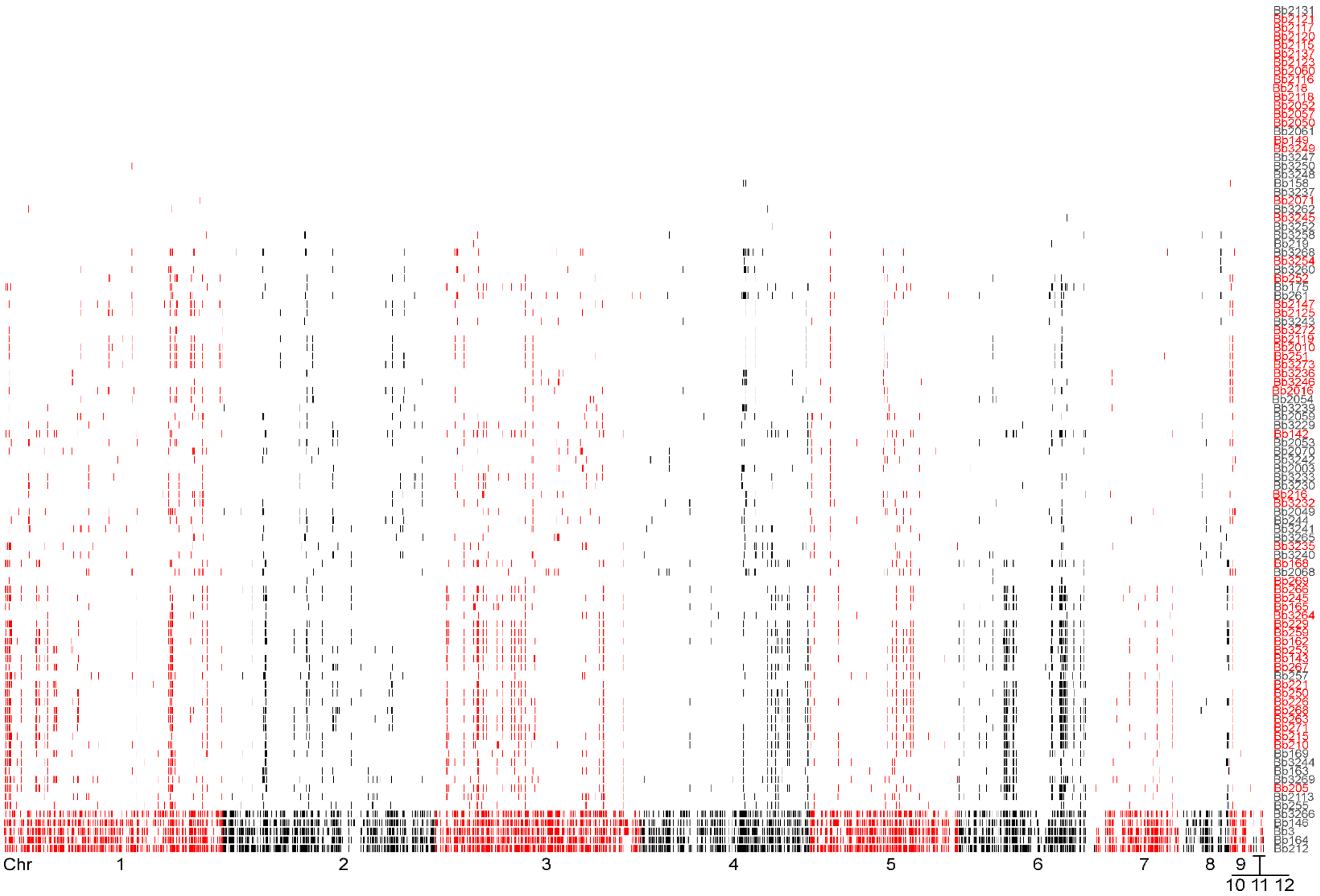

Fig. S8. Genomic-wide analysis of the conserved genomic blocks present between the released and recovered strains. The conserved blocks, i.e., those containing ≥1500 consecutive and identical SNPs, were detected and shown between the released strain Bb17 (MAT1-2 type) and those isolates clustered in the same G1 lineage (Figure 2a). The strains labeled in red contain an opposite mating type (i.e., MAT1-1) to Bb17 with potential for recombination.
