# Supplementary material for: Population genomics and evolution of a fungal pathogen after releasing exotic strains to control insect pests for 20 years
Source: ISME J. 2020 Feb 28;14(6):1422–34. doi: 10.1038/s41396-020-0620-8 (PMC7242398; doi:10.1038/s41396-020-0620-8)
Supplement: Supplementary file 9 — Fig. S9 [file 41396_2020_620_MOESM9_ESM.pdf]

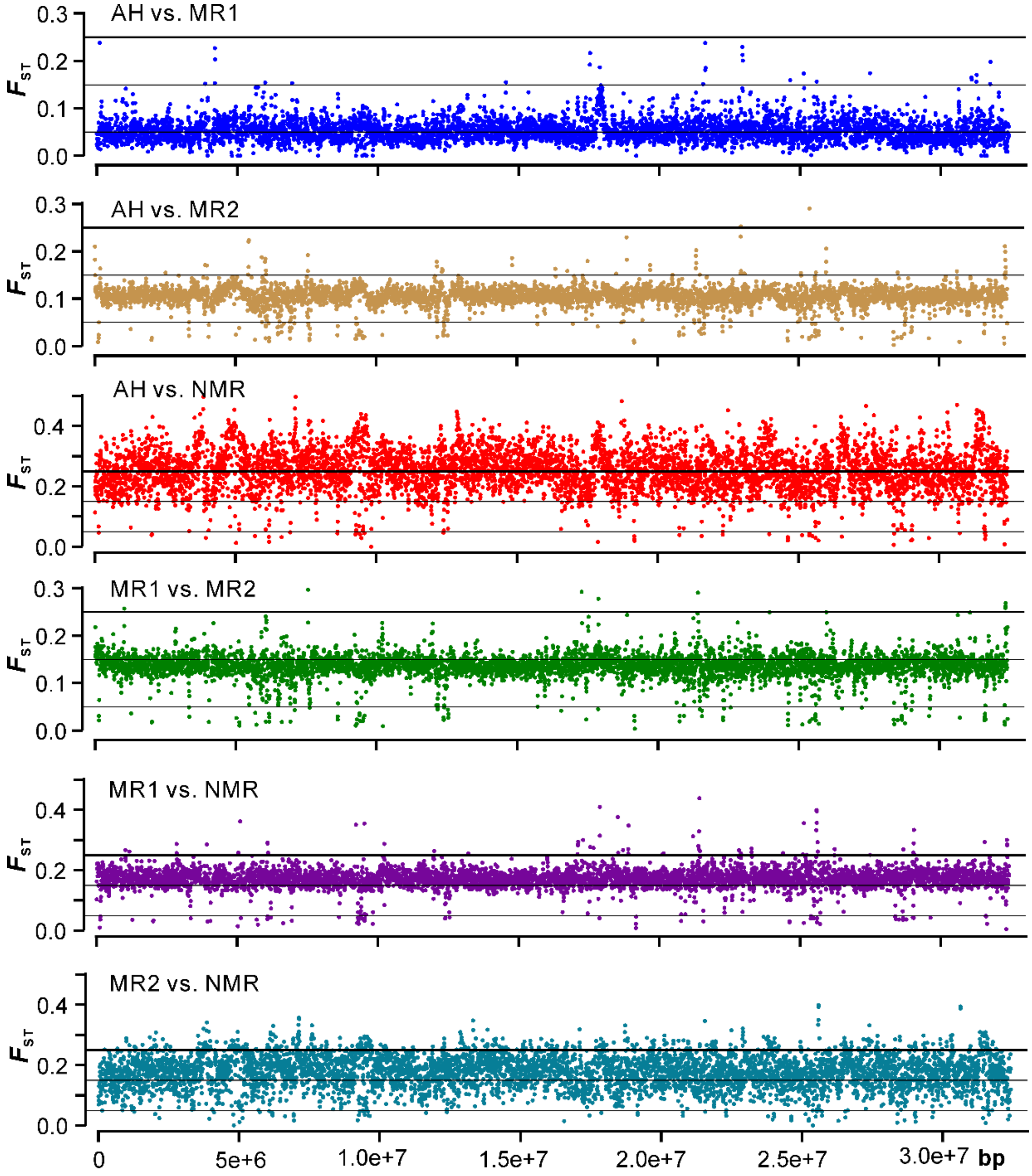

Fig. S9. Genome-wide analysis of genetic divergence between different geographic populations. The value of F_{ST} was calculated across 5 kb windows. The Wright's cutoff lines for $F_{ST} = 0.05$, $F_{ST} = 0.15$ and $F_{ST} = 0.25$ are indicated in each panel. The populations AH (An-Hui Province), MR1 (Monsoon region 1), MR2 (Monsoon region 1) and NMR (Non-monsoon region) are as shown in Figure 1.
