# Supplementary material for: Population genomics and evolution of a fungal pathogen after releasing exotic strains to control insect pests for 20 years
Source: ISME J. 2020 Feb 28;14(6):1422–34. doi: 10.1038/s41396-020-0620-8 (PMC7242398; doi:10.1038/s41396-020-0620-8)
Supplement: Supplementary file 10 — Table S1 [file 41396_2020_620_MOESM10_ESM.pdf]

**Table S1.** Information of the *Beauveria bassiana* isolates included in this study.

| Strains | RCEF No. | Mating type | Insect hosts | Insect host order abbreviation | Geographic origin | Abbreviation of geographic origin | Collection date (y/m/d-collection no.) |
|---|---|---|---|---|---|---|---|
| Bb13 | RCEF0013 | MAT1-2 | Lepidoptera: Lasiocampidae, *Dendrolimus punctatus* | LEP | / | / | / |
| Bb17 | RCEF0017 | MAT1-2 | Lepidoptera: Lasiocampidae, *Dendrolimus punctatus* | LEP | / | / | / |
| Bb3 | RCEF0003 | MAT1-2 | Lepidoptera: Lasiocampidae, *Dendrolimus punctatus* | LEP | An-Hui | AH | 1997/11/27-05 |
| Bb4 | RCEF0004 | MAT1-2 | Lepidoptera: Lasiocampidae, *Dendrolimus punctatus* | LEP | An-Hui | AH | 1997/11/28-01 |
| Bb7 | RCEF0007 | MAT1-2 | Lepidoptera: Lasiocampidae, *Dendrolimus punctatus* | LEP | An-Hui | AH | 1997/11/30-02 |
| Bb8 | RCEF0008 | MAT1-2 | Lepidoptera: Lasiocampidae, *Dendrolimus punctatus* | LEP | An-Hui | AH | 1997/11/30-03 |
| Bb9 | RCEF0009 | MAT1-2 | Lepidoptera: Lasiocampidae, *Dendrolimus punctatus* | LEP | An-Hui | AH | 1997/11/30-04 |
| Bb122 | RCEF0303 | MAT1-1 | Coleoptera: Curculionidae | COL | An-Hui | AH | 1998/01/10-01 |
| Bb124 | RCEF0305 | MAT1-1 | Coleoptera: Curculionidae | COL | An-Hui | AH | 1998/01/10-15 |
| Bb125 | RCEF0306 | MAT1-2 | Orthoptera | ORT | An-Hui | AH | 1998/01/07-25 |
| Bb126 | RCEF0307 | MAT1-2 | Coleoptera: Curculionidae | COL | An-Hui | AH | 1998/01/07-27 |
| Bb142 | RCEF0323 | MAT1-1 | Coleoptera: Curculionidae | COL | An-Hui | AH | 1998/04/05-04 |
| Bb143 | RCEF0324 | MAT1-1 | Orthoptera: Acrididae | ORT | An-Hui | AH | 1998/04/05-06 |
| Bb145 | RCEF0326 | MAT1-2 | Coleoptera: Curculionidae | COL | An-Hui | AH | 1998/04/04-12 |
| Bb146 | RCEF0327 | MAT1-2 | Coleoptera: Curculionidae | COL | An-Hui | AH | 1998/04/04-21 |
| Bb148 | RCEF0329 | MAT1-1 | Coleoptera: Curculionidae | COL | An-Hui | AH | 1998/04/06-19 |
| Bb149 | RCEF0330 | MAT1-1 | Hemiptera: Pentatomidae | HEM | An-Hui | AH | 1998/04/05-23 |
| Bb150 | RCEF0331 | MAT1-2 | Hymenoptera: Vespidae | HYM | An-Hui | AH | 1998/04/05-26 |
| Bb158 | RCEF0339 | MAT1-1/2 | Hymenoptera: Formicidae | HYM | An-Hui | AH | 1998/01/08-35 |
| Bb162 | RCEF0343 | MAT1-1 | Hemiptera: Alydidae, *Riptortus pedestris* | HEM | An-Hui | AH | 1998/05/07-38 |
| Bb163 | RCEF0344 | MAT1-2 | Coleoptera: Curculionidae, *Sympiezomias* sp. | COL | An-Hui | AH | 1998/05/07-48 |
| Bb164 | RCEF0345 | MAT1-2 | Hemiptera: Pentatomidae, *Halyomorpha picus* | HEM | An-Hui | AH | 1998/05/07-28 |
| Bb165 | RCEF0346 | MAT1-1 | Hemiptera: Cercopidae | HEM | An-Hui | AH | 1998/05/07-30 |
| Bb167 | RCEF0348 | MAT1-2 | Coleoptera: Curculionidae | COL | An-Hui | AH | 1998/05/08-01 |
| Bb168 | RCEF0349 | MAT1-1 | Hymenoptera: Ichneumonidae | HYM | An-Hui | AH | 1998/05/08-12 |
| Bb169 | RCEF0350 | MAT1-2 | Coleoptera: Coccineidae, *Coccinea septempunctata* | COL | An-Hui | AH | 1998/05/08-15 |

| | | | | | | | |
|---|---|---|---|---|---|---|---|
| Bb174 | RCEF0355 | MAT1-1 | Lepidoptera: Lasiocampidae, *Dendrolimus punctatus* | LEP | An-Hui | AH | 1998/05/10-08 |
| Bb175 | RCEF0356 | MAT1-1/2 | Hymenoptera: Apidae | HYM | An-Hui | AH | 1998/05/07-31 |
| Bb178 | RCEF0359 | MAT1-2 | Lepidoptera | LEP | An-Hui | AH | 1998/05/08-22 |
| Bb181 | RCEF0362 | MAT1-1 | Hemiptera: Plataspidae | HEM | An-Hui | AH | 1998/05/10-03 |
| Bb183 | RCEF0364 | MAT1-2 | Hemiptera: Plataspidae | HEM | An-Hui | AH | 1998/05/10-05 |
| Bb185 | RCEF0366 | MAT1-1 | Lepidoptera | LEP | An-Hui | AH | 1998/05/10-09 |
| Bb205 | RCEF0391 | MAT1-1 | Coleoptera: Chrysomelidae | COL | An-Hui | AH | 1998/07/08-01 |
| Bb208 | RCEF0394 | MAT1-2 | Coleoptera: Chrysomelidae | COL | An-Hui | AH | 1998/07/08-07 |
| Bb210 | RCEF0396 | MAT1-1 | Coleoptera: Curculionidae, *Sympiezomias* sp. | COL | An-Hui | AH | 1998/07/08-09 |
| Bb211 | RCEF0397 | MAT1-1 | Coleoptera: Curculionidae, *Sympiezomias* sp. | COL | An-Hui | AH | 1998/07/08-14 |
| Bb212 | RCEF0398 | MAT1-2 | Coleoptera: Chrysomelidae | COL | An-Hui | AH | 1998/07/08-16 |
| Bb215 | RCEF0401 | MAT1-1 | Coleoptera: Chrysomelidae | COL | An-Hui | AH | 1998/07/08-19 |
| Bb216 | RCEF0402 | MAT1-1 | Coleoptera: Chrysomelidae | COL | An-Hui | AH | 1998/07/08-21 |
| Bb218 | RCEF0404 | MAT1-1 | Hemiptera: Pentatomidae, *Halyomorpha picus* | HEM | An-Hui | AH | 1998/09/10-01 |
| Bb219 | RCEF0405 | MAT1-1/2 | Coleoptera: Curculionidae | COL | An-Hui | AH | 1998/09/10-02 |
| Bb221 | RCEF0407 | MAT1-1 | Coleoptera: Curculionidae, *Sympiezomias* sp. | COL | An-Hui | AH | 1998/09/10-06 |
| Bb226 | RCEF0412 | MAT1-1 | Coleoptera: Curculionidae | COL | An-Hui | AH | 1998/09/10-14 |
| Bb229 | RCEF0415 | MAT1-1 | Coleoptera: Curculionidae | COL | An-Hui | AH | 1998/09/10-17 |
| Bb242 | RCEF0471 | MAT1-1 | Lepidoptera: Lymantriidae, *Lymantria dissoluta* | LEP | An-Hui | AH | 1999/01/27-03 |
| Bb244 | RCEF0473 | MAT1-2 | Lepidoptera: Lymantriidae, *Lymantria dissoluta* | LEP | An-Hui | AH | 1999/01/27-06 |
| Bb245 | RCEF0474 | MAT1-1 | Coleoptera: Curculionidae | COL | An-Hui | AH | 1999/01/27-07 |
| Bb248 | RCEF0477 | MAT1-2 | Lepidoptera: Lasiocampidae, *Dendrolimus punctatus* | LEP | An-Hui | AH | 1999/01/27-21 |
| Bb249 | RCEF0478 | MAT1-2 | Lepidoptera: Lymantriidae, *Lymantria dissoluta* | LEP | An-Hui | AH | 1999/01/27-22 |
| Bb250 | RCEF0479 | MAT1-1 | Lepidoptera: Lasiocampidae, *Dendrolimus punctatus* | LEP | An-Hui | AH | 1999/01/27-27 |
| Bb251 | RCEF0480 | MAT1-1 | Coleoptera: Curculionidae, *Sympiezomias* sp. | COL | An-Hui | AH | 1999/01/27-29 |
| Bb252 | RCEF0481 | MAT1-1 | Lepidoptera: Lasiocampidae, *Dendrolimus punctatus* | LEP | An-Hui | AH | 1999/01/27-30 |
| Bb253 | RCEF0482 | MAT1-1 | Hemiptera: Reduviidae | HEM | An-Hui | AH | 1999/01/27-33 |
| Bb255 | RCEF0484 | MAT1-2 | Coleoptera: Curculionidae | COL | An-Hui | AH | 1998/11/27-11 |
| Bb257 | RCEF0486 | MAT1-2 | Coleoptera: Curculionidae | COL | An-Hui | AH | 1998/11/27-15 |
| Bb259 | RCEF0488 | MAT1-1 | Lepidoptera: Lymantriidae, *Lymantria dissoluta* | LEP | An-Hui | AH | 1998/11/27-17 |
| Bb261 | RCEF0490 | MAT1-2 | Hymenoptera: Formicidae | HYM | An-Hui | AH | 1998/11/27-22 |
| Bb262 | RCEF0491 | MAT1-1 | Lepidoptera: Aegeriidae | LEP | An-Hui | AH | 1998/11/27-24 |

| | | | | | | | |
|---|---|---|---|---|---|---|---|
| Bb263 | RCEF0492 | MAT1-1 | Coleoptera: Curculionidae | COL | An-Hui | AH | 1998/11/27-26 |
| Bb266 | RCEF0495 | MAT1-1 | Coleoptera: Curculionidae, *Sympiezomias* sp. | COL | An-Hui | AH | 1998/11/27-30 |
| Bb267 | RCEF0496 | MAT1-1 | Hemiptera: Reduviidae | HEM | An-Hui | AH | 1998/11/27-32 |
| Bb268 | RCEF0497 | MAT1-1 | Diptera | DIP | An-Hui | AH | 1998/11/27-33 |
| Bb269 | RCEF0498 | MAT1-1 | Orthoptera: Tettigoniidae | ORT | An-Hui | AH | 1998/11/27-35 |
| Bb271 | RCEF0500 | MAT1-1 | Coleoptera: Curculionidae | COL | An-Hui | AH | 1998/11/27-38 |
| Bb1982 | RCEF3483 | MAT1-1 | Lepidoptera: Lasiocampidae, *Dendrolimus punctatus* | LEP | An-Hui | AH | 2007/05/11-60 |
| Bb1999 | RCEF3569 | MAT1-2 | Coleoptera: Coccineidae | COL | An-Hui | AH | 2007/06/19-41 |
| Bb2000 | RCEF3570 | MAT1-1 | Lepidoptera: Lasiocampidae, *Dendrolimus punctatus* | LEP | An-Hui | AH | 2007/06/19-43 |
| Bb2001 | RCEF3571 | MAT1-1 | Lepidoptera: Lasiocampidae, *Dendrolimus punctatus* | LEP | An-Hui | AH | 2007/06/19-46 |
| Bb2003 | RCEF3573 | MAT1-2 | Diptera | DIP | An-Hui | AH | 2007/06/19-85 |
| Bb2006 | RCEF3602 | MAT1-1/2 | Coleoptera: Curculionidae | COL | An-Hui | AH | 2007/06/19-103 |
| Bb2007 | RCEF3609 | MAT1-1 | Hymenoptera | HYM | An-Hui | AH | 2007/06/18-13 |
| Bb2009 | RCEF3611 | MAT1-2 | Coleoptera: Curculionidae, *Shirahoshizo juglandis* | COL | An-Hui | AH | 2007/06/18-29 |
| Bb2010 | RCEF3612 | MAT1-1 | Coleoptera: Curculionidae, *Shirahoshizo juglandis* | COL | An-Hui | AH | 2007/06/18-30 |
| Bb2014 | RCEF3616 | MAT1-2 | Lepidoptera: Lasiocampidae, *Dendrolimus punctatus* | LEP | An-Hui | AH | 2007/06/19-65 |
| Bb2016 | RCEF3618 | MAT1-1 | Lepidoptera: Lasiocampidae, *Dendrolimus punctatus* | LEP | An-Hui | AH | 2007/06/19-90 |
| Bb2049 | RCEF3759 | MAT1-2 | Hymenoptera: Formicidae | HYM | An-Hui | AH | 2007/07/25-09 |
| Bb2050 | RCEF3760 | MAT1-1 | Coleoptera: Coccineidae | COL | An-Hui | AH | 2007/07/25-10 |
| Bb2052 | RCEF3762 | MAT1-1 | Coleoptera: Coccineidae | COL | An-Hui | AH | 2007/07/25-13 |
| Bb2053 | RCEF3763 | MAT1-2 | Coleoptera: Curculionidae, *Shirahoshizo juglandis* | COL | An-Hui | AH | 2007/07/25-21 |
| Bb2054 | RCEF3764 | MAT1-2 | Coleoptera: Chrysomelidae | COL | An-Hui | AH | 2007/07/25-22 |
| Bb2056 | RCEF3766 | MAT1-1 | Lepidoptera: Lymantriidae, *Dasychira* sp. | LEP | An-Hui | AH | 2007/07/25-28 |
| Bb2057 | RCEF3767 | MAT1-1 | Hemiptera: Plataspidae | HEM | An-Hui | AH | 2007/07/25-29 |
| Bb2059 | RCEF3769 | MAT1-2 | Coleoptera: Curculionidae | COL | An-Hui | AH | 2007/07/26-40 |
| Bb2060 | RCEF3770 | MAT1-1 | Hemiptera: Plataspidae | HEM | An-Hui | AH | 2007/07/26-42 |
| Bb2061 | RCEF3771 | MAT1-2 | Diptera | DIP | An-Hui | AH | 2007/07/26-50 |
| Bb2062 | RCEF3772 | MAT1-2 | Coleoptera: Curculionidae | COL | An-Hui | AH | 2007/07/26-63 |
| Bb2063 | RCEF3773 | MAT1-1 | Lepidoptera: Lasiocampidae, *Dendrolimus punctatus* | LEP | An-Hui | AH | 2007/07/26-64 |
| Bb2064 | RCEF3774 | MAT1-1 | Coleoptera: Coccineidae | COL | An-Hui | AH | 2007/07/26-69 |
| Bb2068 | RCEF3778 | MAT1-2 | Diptera: Culicidae | DIP | An-Hui | AH | 2007/07/26-80 |
| Bb2070 | RCEF3780 | MAT1-2 | Coleoptera: Coccineidae | COL | An-Hui | AH | 2007/07/26-84 |

| | | | | | | | |
|---|---|---|---|---|---|---|---|
| Bb2071 | RCEF3781 | MAT1-1 | Hymenoptera: Formicidae | HYM | An-Hui | AH | 2007/07/26-85 |
| Bb2072 | RCEF3782 | MAT1-2 | Hemiptera: Coreidae | HEM | An-Hui | AH | 2007/07/26-88 |
| Bb2113 | RCEF3920 | MAT1-2 | Hemiptera: Coreidae | HEM | An-Hui | AH | 2007/08/21-25 |
| Bb2115 | RCEF3922 | MAT1-1 | Hemiptera: Coreidae | HEM | An-Hui | AH | 2007/08/21-35 |
| Bb2116 | RCEF3923 | MAT1-1 | Hemiptera: Coreidae | HEM | An-Hui | AH | 2007/08/21-37 |
| Bb2117 | RCEF3924 | MAT1-1 | Hemiptera: Coreidae | HEM | An-Hui | AH | 2007/08/21-47 |
| Bb2118 | RCEF3925 | MAT1-1 | Hemiptera: Coreidae | HEM | An-Hui | AH | 2007/08/21-51 |
| Bb2119 | RCEF3926 | MAT1-1 | Coleoptera: Curculionidae | COL | An-Hui | AH | 2007/08/21-57 |
| Bb2120 | RCEF3927 | MAT1-1 | Hemiptera: Coreidae | HEM | An-Hui | AH | 2007/08/21-61 |
| Bb2121 | RCEF3928 | MAT1-1 | Hemiptera: Coreidae | HEM | An-Hui | AH | 2007/08/21-65 |
| Bb2123 | RCEF3930 | MAT1-1 | Hemiptera: Plataspidae | HEM | An-Hui | AH | 2007/08/21-68 |
| Bb2125 | RCEF3932 | MAT1-1 | Lepidoptera: Sphingidae | LEP | An-Hui | AH | 2007/08/21-72 |
| Bb2126 | RCEF3933 | MAT1-2 | Coleoptera: Curculionidae | COL | An-Hui | AH | 2007/08/21-75 |
| Bb2131 | RCEF4012 | MAT1-2 | Lepidoptera: Lasiocampidae, *Dendrolimus punctatus* | LEP | An-Hui | AH | 2007/09/24-01 |
| Bb2132 | RCEF4013 | MAT1-2 | Lepidoptera: Lasiocampidae, *Dendrolimus punctatus* | LEP | An-Hui | AH | 2007/09/24-17 |
| Bb2135 | RCEF4016 | MAT1-2 | Lepidoptera: Lasiocampidae | LEP | An-Hui | AH | 2007/09/24-45 |
| Bb2136 | RCEF4017 | MAT1-1 | Hymenoptera: Formicidae | HYM | An-Hui | AH | 2007/09/25-50 |
| Bb2137 | RCEF4018 | MAT1-1 | Coleoptera: Curculionidae | COL | An-Hui | AH | 2007/09/25-75 |
| Bb2138 | RCEF4019 | MAT1-1 | Coleoptera: Curculionidae, *Shirahoshizo juglandis* | COL | An-Hui | AH | 2007/09/25-79 |
| Bb2139 | RCEF4020 | MAT1-1 | Coleoptera: Curculionidae | COL | An-Hui | AH | 2007/09/25-82 |
| Bb2147 | RCEF4046 | MAT1-1 | Coleoptera: Curculionidae | COL | An-Hui | AH | 2007/11/22-33 |
| Bb3229 | RCEF6365 | MAT1-2 | Coleoptera: Curculionidae, *Shirahoshizo juglandis* | COL | An-Hui | AH | 2017/07/17-02 |
| Bb3230 | RCEF6366 | MAT1-2 | Coleoptera: Carabidae | COL | An-Hui | AH | 2017/07/17-03 |
| Bb3231 | RCEF6367 | MAT1-2 | Coleoptera: Chrysomelidae, *Smaragdina* sp. | COL | An-Hui | AH | 2017/07/17-04 |
| Bb3232 | RCEF6368 | MAT1-1 | Coleoptera: Chrysomelidae, *Smragdina* sp. | COL | An-Hui | AH | 2017/07/17-05 |
| Bb3233 | RCEF6369 | MAT1-2 | Coleoptera: Chrysomelidae | COL | An-Hui | AH | 2017/07/17-06 |
| Bb3235 | RCEF6371 | MAT1-1 | Hymenoptera | HYM | An-Hui | AH | 2017/07/17-08 |
| Bb3236 | RCEF6372 | MAT1-1 | Coleoptera: Chrysomelidae | COL | An-Hui | AH | 2017/07/17-09 |
| Bb3237 | RCEF6373 | MAT1-2 | Coleoptera: Carabidae | COL | An-Hui | AH | 2017/07/17-11 |
| Bb3238 | RCEF6374 | MAT1-1 | Coleoptera: Chrysomelidae | COL | An-Hui | AH | 2017/07/17-12 |
| Bb3239 | RCEF6375 | MAT1-2 | Coleoptera: Chrysomelidae | COL | An-Hui | AH | 2017/07/17-14 |
| Bb3240 | RCEF6376 | MAT1-2 | Hymenoptera: Formicidae | HYM | An-Hui | AH | 2017/07/17-15 |

| | | | | | | | |
|---|---|---|---|---|---|---|---|
| Bb3241 | RCEF6377 | MAT1-2 | Diptera: Celyphidae | DIP | An-Hui | AH | 2017/07/17-16 |
| Bb3242 | RCEF6378 | MAT1-2 | Lepidoptera | LEP | An-Hui | AH | 2017/07/17-17 |
| Bb3243 | RCEF6379 | MAT1-2 | Hemiptera: Reduviidae | HEM | An-Hui | AH | 2017/07/17-18 |
| Bb3244 | RCEF6380 | MAT1-2 | Coleoptera: Elateridae, *Campsosternus auratus* | COL | An-Hui | AH | 2017/07/17-19 |
| Bb3245 | RCEF6381 | MAT1-1 | Coleoptera: Chrysomelidae | COL | An-Hui | AH | 2017/07/17-20 |
| Bb3246 | RCEF6382 | MAT1-1 | Coleoptera: Eumolpidae | COL | An-Hui | AH | 2017/07/17-21 |
| Bb3247 | RCEF6383 | MAT1-2 | Coleoptera: Chrysomelidae | COL | An-Hui | AH | 2017/07/17-22 |
| Bb3248 | RCEF6384 | MAT1-2 | Coleoptera: Chrysomelidae, *Smaragdina* sp. | COL | An-Hui | AH | 2017/07/17-23 |
| Bb3249 | RCEF6385 | MAT1-1 | Coleoptera | COL | An-Hui | AH | 2017/07/17-24 |
| Bb3250 | RCEF6386 | MAT1-2 | Coleoptera: Chrysomelidae, *Smaragdina* sp. | COL | An-Hui | AH | 2017/07/17-25 |
| Bb3251 | RCEF6387 | MAT1-2 | Hemiptera: Cicadelidae | HEM | An-Hui | AH | 2017/07/17-26 |
| Bb3252 | RCEF6388 | MAT1-2 | Coleoptera: Chrysomelidae | COL | An-Hui | AH | 2017/07/17-27 |
| Bb3253 | RCEF6389 | MAT1-2 | Coleoptera: Curculionidae | COL | An-Hui | AH | 2017/07/17-28 |
| Bb3254 | RCEF6390 | MAT1-1 | Hymenoptera: Formicidae | HYM | An-Hui | AH | 2017/07/17-29 |
| Bb3257 | RCEF6393 | MAT1-2 | Coleoptera: Curculionidae | COL | An-Hui | AH | 2017/08/24-04 |
| Bb3258 | RCEF6394 | MAT1-2 | Lepidoptera | LEP | An-Hui | AH | 2017/08/24-05 |
| Bb3259 | RCEF6395 | MAT1-2 | Hymenoptera: Formicidae | HYM | An-Hui | AH | 2017/08/24-06 |
| Bb3260 | RCEF6396 | MAT1-2 | Coleoptera: Chrysomelidae, *Smaragdina* sp. | COL | An-Hui | AH | 2017/08/25-17 |
| Bb3261 | RCEF6397 | MAT1-2 | Hemiptera: Pentatomidae | HEM | An-Hui | AH | 2017/08/25-26 |
| Bb3262 | RCEF6398 | MAT1-2 | Hymenoptera: Formicidae | HYM | An-Hui | AH | 2017/08/25-27 |
| Bb3263 | RCEF6399 | MAT1-2 | Coleoptera: Chrysomelidae | COL | An-Hui | AH | 2017/08/25-31 |
| Bb3264 | RCEF6400 | MAT1-1 | Lepidoptera | LEP | An-Hui | AH | 2017/08/25-33 |
| Bb3265 | RCEF6401 | MAT1-2 | Coleoptera: Chrysomelidae | COL | An-Hui | AH | 2017/08/25-36 |
| Bb3266 | RCEF6402 | MAT1-2 | Coleoptera: Chrysomelidae | COL | An-Hui | AH | 2017/08/25-40 |
| Bb3267 | RCEF6403 | MAT1-2 | Lepidoptera | LEP | An-Hui | AH | 2017/08/25-44 |
| Bb3268 | RCEF6404 | MAT1-2 | Coleoptera: Curculionidae | COL | An-Hui | AH | 2017/09/25-01 |
| Bb3269 | RCEF6405 | MAT1-2 | Lepidoptera | LEP | An-Hui | AH | 2017/09/25-04 |
| Bb3270 | RCEF6406 | MAT1-1 | Coleoptera: Chrysomelidae | COL | An-Hui | AH | 2017/09/25-05 |
| Bb3271 | RCEF6407 | MAT1-1 | Orthoptera: Catantopidae | ORT | An-Hui | AH | 2017/09/25-06 |
| Bb3272 | RCEF6408 | MAT1-1 | Coleoptera: Curculionidae | COL | An-Hui | AH | 2017/09/25-13 |
| Bb3273 | RCEF6409 | MAT1-1 | Coleoptera: Curculionidae | COL | An-Hui | AH | 2017/09/25-14 |
| Bb3274 | RCEF6410 | MAT1-1 | Coleoptera: Curculionidae | COL | An-Hui | AH | 2017/09/25-15 |

| | | | | | | | |
|---|---|---|---|---|---|---|---|
| Bb2461 | RCEF4993 | MAT1-2 | Hemiptera | HEM | Fu-Jiang | FJ | 2010/07/14-12 |
| Bb2462 | RCEF4994 | MAT1-1 | Hymenoptera: Formicidae | HYM | Fu-Jiang | FJ | 2010/07/14-14 |
| Bb2464 | RCEF4996 | MAT1-1 | Coleoptera: Curculionidae | COL | Fu-Jiang | FJ | 2010/07/15-1 |
| Bb2468 | RCEF5000 | MAT1-1 | Hemiptera: Membracidae | HEM | Fu-Jiang | FJ | 2010/07/15-20 |
| Bb2469 | RCEF5001 | MAT1-2 | Hemiptera: Membracidae | HEM | Fu-Jiang | FJ | 2010/07/16-1 |
| Bb2472 | RCEF5004 | MAT1-2 | Hemiptera: Membracidae | HEM | Fu-Jiang | FJ | 2010/07/16-4 |
| Bb2477 | RCEF5009 | MAT1-2 | Hemiptera: Membracidae | HEM | Fu-Jiang | FJ | 2010/07/16-9 |
| Bb2332 | RCEF4649 | MAT1-2 | Hymenoptera: Formicidae | HYM | Guang-Xi | GX | 2009/07/21-10 |
| Bb2334 | RCEF4651 | MAT1-2 | Coleoptera: Chrysomelidae | COL | Guang-Xi | GX | 2009/07/22-07 |
| Bb2336 | RCEF4653 | MAT1-1 | Hemiptera: Membracidae | HEM | Guang-Xi | GX | 2009/07/22-12 |
| Bb2337 | RCEF4654 | MAT1-1 | Coleoptera: Curculionidae | COL | Guang-Xi | GX | 2009/07/22-13 |
| Bb2338 | RCEF4655 | MAT1-2 | Diptera | DIP | Guang-Xi | GX | 2009/07/22-15 |
| Bb2340 | RCEF4657 | MAT1-1 | Lepidoptera | LEP | Guang-Xi | GX | 2009/07/22-18 |
| Bb2343 | RCEF4660 | MAT1-2 | Coleoptera: Coccineidae | COL | Guang-Xi | GX | 2009/07/22-32 |
| Bb2345 | RCEF4662 | MAT1-2 | Lepidoptera | LEP | Guang-Xi | GX | 2009/07/22-34 |
| Bb2348 | RCEF4665 | MAT1-2 | Diptera | DIP | Guang-Xi | GX | 2009/07/22-47 |
| Bb2349 | RCEF4666 | MAT1-2 | Coleoptera: Curculionidae | COL | Guang-Xi | GX | 2009/07/22-49 |
| Bb20 | RCEF0225 | MAT1-1 | Coleoptera | COL | Gui-Zhou | GZ | 1996/08/06-10 |
| Bb29 | RCEF0229 | MAT1-2 | Hymenoptera: Ichneumonidae | HYM | Gui-Zhou | GZ | 1996/08/07-19 |
| Bb42 | RCEF0232 | MAT1-2 | Coleoptera: Cistelidae | COL | Gui-Zhou | GZ | 1996/08/08-11 |
| Bb49 | RCEF0235 | MAT1-1 | Hemiptera | HEM | Gui-Zhou | GZ | 1996/08/08-43 |
| Bb50 | RCEF0236 | MAT1-1 | Hymenoptera: Tenthredinidae | HYM | Gui-Zhou | GZ | 1996/08/08-51 |
| Bb54 | RCEF0237 | MAT1-1 | Lepidoptera | LEP | Gui-Zhou | GZ | 1996/08/08-54 |
| Bb55 | RCEF0238 | MAT1-1 | Lepidoptera | LEP | Gui-Zhou | GZ | 1996/08/08-70 |
| Bb2984 | RCEF5771 | MAT1-1 | Coleoptera: Carabidae | COL | Gui-Zhou | GZ | 2011/10/24-18 |
| Bb3214 | RCEF6313 | MAT1-2 | Hymenoptera: Apidae | HYM | Gui-Zhou | GZ | 2014/10/28-1 |
| Bb3215 | RCEF6314 | MAT1-1 | Hymenoptera: Diprionidae | HYM | Gui-Zhou | GZ | 2014/11/10-1 |
| Bb3218 | RCEF6317 | MAT1-1 | Hemiptera: Fulgoridae, *Ricania* sp. | HEM | Gui-Zhou | GZ | 2014/11/05-1 |
| Bb2693 | RCEF5360 | MAT1-1 | Coleoptera: Chrysomelidae | COL | He-Bei | HB | 2010/09/24-48 |
| Bb2694 | RCEF5361 | MAT1-2 | Hemiptera: Plataspidae | HEM | He-Bei | HB | 2010/09/24-49 |
| Bb2695 | RCEF5362 | MAT1-1/2 | Diptera | DIP | He-Bei | HB | 2010/09/24-50 |
| Bb2696 | RCEF5363 | MAT1-1 | Coleoptera: Chrysomelidae | COL | He-Bei | HB | 2010/09/24-51 |

| | | | | | | | |
|---|---|---|---|---|---|---|---|
| Bb2699 | RCEF5366 | MAT1-2 | Coleoptera: Chrysomelidae | COL | He-Bei | HB | 2010/09/24-54 |
| Bb2700 | RCEF5367 | MAT1-1 | Coleoptera: Curculionidae | COL | He-Bei | HB | 2010/09/24-55 |
| Bb2701 | RCEF5368 | MAT1-1 | Lepidoptera | LEP | He-Bei | HB | 2010/09/24-56 |
| Bb2702 | RCEF5369 | MAT1-1 | Lepidoptera | LEP | He-Bei | HB | 2010/09/24-58 |
| Bb2703 | RCEF5370 | MAT1-2 | Hemiptera: Cicadelidae | HEM | He-Bei | HB | 2010/09/24-59 |
| Bb2901 | RCEF5657 | MAT1-2 | Hymenoptera: Formicidae | HYM | Inner Mongol | IM | 2011/08/08-2 |
| Bb2903 | RCEF5659 | MAT1-1 | Hemiptera: Pentatomidae | HEM | Inner Mongol | IM | 2011/08/08-4 |
| Bb2908 | RCEF5664 | MAT1-2 | Coleoptera: Chrysomelidae | COL | Inner Mongol | IM | 2011/08/08-12 |
| Bb2910 | RCEF5666 | MAT1-1 | Coleoptera: Tenebrionidae | COL | Inner Mongol | IM | 2011/08/08-14 |
| Bb2915 | RCEF5671 | MAT1-2 | Lepidoptera: Lasiocampidae | LEP | Inner Mongolia | IM | 2011/08/08-23 |
| Bb2916 | RCEF5672 | MAT1-1 | Coleoptera: Chrysomelidae | COL | Inner Mongol | IM | 2011/08/08-24 |
| Bb2924 | RCEF5680 | MAT1-2 | Coleoptera: Carabidae | COL | Inner Mongol | IM | 2011/08/08-38 |
| Bb2928 | RCEF5684 | MAT1-1 | Lepidoptera | LEP | Inner Mongol | IM | 2011/08/08-48 |
| Bb2929 | RCEF5685 | MAT1-1 | Coleoptera: Curculionidae | COL | Inner Mongol | IM | 2011/08/08-50 |
| Bb2513 | RCEF5147 | MAT1-1/2 | Coleoptera: Chrysomelidae | COL | Ning-Xia | NX | 2010/09/17-2 |
| Bb2583 | RCEF5237 | MAT1-2 | Hemiptera: Cicadelidae | HEM | Ning-Xia | NX | 2010/09/17-1 |
| Bb2587 | RCEF5241 | MAT1-2 | Diptera: Culicidae | DIP | Ning-Xia | NX | 2010/09/17-6 |
| Bb2589 | RCEF5243 | MAT1-1 | Hemiptera: Pentatomidae | HEM | Ning-Xia | NX | 2010/09/17-12 |
| Bb2590 | RCEF5244 | MAT1-2 | Hemiptera: Coreidae | HEM | Ning-Xia | NX | 2010/09/17-13 |
| Bb2592 | RCEF5246 | MAT1-2 | Coleoptera: Coccineidae | COL | Ning-Xia | NX | 2010/09/17-15 |
| Bb2594 | RCEF5248 | MAT1-1 | Hemiptera: Pentatomidae | HEM | Ning-Xia | NX | 2010/09/17-17 |
| Bb2596 | RCEF5250 | MAT1-1 | Hemiptera: Fulgoridae | HEM | Ning-Xia | NX | 2010/09/17-21 |
| Bb2606 | RCEF5260 | MAT1-1 | Lepidoptera: Pyralidae | LEP | Ning-Xia | NX | 2010/09/17-37 |
| Bb2609 | RCEF5263 | MAT1-2 | Coleoptera: Chrysomelidae | COL | Ning-Xia | NX | 2010/09/17-40 |
| Bb2613 | RCEF5275 | MAT1-1 | Hymenoptera: Apidae | HYM | Ning-Xia | NX | 2010/09/17-48 |
| Bb2615 | RCEF5277 | MAT1-1 | Lepidoptera | LEP | Ning-Xia | NX | 2010/09/17-54 |
| Bb2623 | RCEF5285 | MAT1-2 | Diptera | DIP | Ning-Xia | NX | 2010/09/17-66 |
| Bb2629 | RCEF5291 | MAT1-1 | Orthoptera: Tetrigidae | ORT | Ning-Xia | NX | 2010/09/17-75 |
| Bb2665 | RCEF5326 | MAT1-1 | Coleoptera: Coccineidae | COL | Ning-Xia | NX | 2010/09/17-126 |
| Bb2671 | RCEF5332 | MAT1-2 | Coleoptera | COL | Ning-Xia | NX | 2010/09/17-132 |
| Bb2721 | RCEF5388 | MAT1-1/2 | Hemiptera | HEM | Ning-Xia | NX | 2010/09/17-34 |
| Bb2723 | RCEF5390 | MAT1-2 | Hymenoptera | HYM | Ning-Xia | NX | 2010/09/17-49 |

| | | | | | | | |
|---|---|---|---|---|---|---|---|
| Bb3055 | RCEF5886 | MAT1-2 | Coleoptera: Chrysomelidae | COL | Qing-Hai | QH | 2012/06/11-1 |
| Bb3057 | RCEF5888 | MAT1-2 | Coleoptera: Curculionidae | COL | Qing-Hai | QH | 2012/06/11-9 |
| Bb3059 | RCEF5890 | MAT1-2 | Hemiptera: Membracidae | HEM | Qing-Hai | QH | 2012/06/11-14 |
| Bb3062 | RCEF5893 | MAT1-2 | Lepidoptera | LEP | Qing-Hai | QH | 2012/06/11-24 |
| Bb3063 | RCEF5894 | MAT1-1 | Coleoptera: Carabidae | COL | Qing-Hai | QH | 2012/06/11-27 |
| Bb3070 | RCEF5901 | MAT1-2 | Diptera: Culicidae | DIP | Qing-Hai | QH | 2012/06/11-49 |
| Bb2490 | RCEF5124 | MAT1-2 | Coleoptera: Curculionidae | COL | Shan-Xi | SX | 2010/09/23-5 |
| Bb2491 | RCEF5125 | MAT1-2 | Hemiptera: Pentatomidae | HEM | Shan-Xi | SX | 2010/09/23-8 |
| Bb2492 | RCEF5126 | MAT1-2 | Coleoptera: Chrysomelidae, *Oides decempunctata* | COL | Shan-Xi | SX | 2010/09/23-10 |
| Bb2493 | RCEF5127 | MAT1-2 | Orthoptera: Pyrgomorphidae, *Atractomorpha sinensis* | ORT | Shan-Xi | SX | 2010/09/23-14 |
| Bb2494 | RCEF5128 | MAT1-1 | Coleoptera: Chrysomelidae | COL | Shan-Xi | SX | 2010/09/23-15 |
| Bb2499 | RCEF5133 | MAT1-1 | Hymenoptera: Formicidae | HYM | Shan-Xi | SX | 2010/09/23-36 |
| Bb2501 | RCEF5135 | MAT1-2 | Coleoptera: Epilachninae, *Henosepilachna* sp. | COL | Shan-Xi | SX | 2010/09/23-47 |
| Bb2503 | RCEF5137 | MAT1-1 | Coleoptera: Chrysomelidae | COL | Shan-Xi | SX | 2010/09/23-55 |
| Bb2509 | RCEF5143 | MAT1-2 | Hemiptera: Miridae | HEM | Shan-Xi | SX | 2010/09/23-64 |
| Bb2520 | RCEF5154 | MAT1-2 | Hemiptera: Coreidae | HEM | Shan-Xi | SX | 2010/09/23-100 |
| Bb2521 | RCEF5155 | MAT1-1 | Coleoptera: Chrysomelidae, *Plagiodera versicolora* | COL | Shan-Xi | SX | 2010/09/23-101 |
| Bb2524 | RCEF5158 | MAT1-1 | Hemiptera: Miridae | HEM | Shan-Xi | SX | 2010/09/23-110 |
| Bb2525 | RCEF5159 | MAT1-2 | Orthoptera: Acrididae | ORT | Shan-Xi | SX | 2010/09/23-114 |
| Bb2526 | RCEF5160 | MAT1-2 | Hemiptera: Pentatomidae | HEM | Shan-Xi | SX | 2010/09/23-115 |
| Bb2530 | RCEF5164 | MAT1-1 | Lepidoptera | LEP | Shan-Xi | SX | 2010/09/23-71 |
| Bb2544 | RCEF5178 | MAT1-2 | Hymenoptera: Formicidae | HYM | Shan-Xi | SX | 2010/09/23-153 |
| Bb2566 | RCEF5215 | MAT1-2 | Hymenoptera | HYM | Shan-Xi | SX | 2010/09/23-1 |
| Bb2567 | RCEF5216 | MAT1-2 | Orthoptera: Mantidae | ORT | Shan-Xi | SX | 2010/09/23-12 |
| Bb2574 | RCEF5223 | MAT1-1 | Hemiptera | HEM | Shan-Xi | SX | 2010/09/23-102 |
| Bb3165 | RCEF6160 | MAT1-2 | Coleoptera | COL | Tibet | TB | 2013/07/03-4 |
| Bb3170 | RCEF6165 | MAT1-1 | Coleoptera: Curculionidae | COL | Tibet | TB | 2013/07/04-16 |
| Bb3179 | RCEF6174 | MAT1-2 | Coleoptera | COL | Tibet | TB | 2013/07/05-7 |
| Bb3181 | RCEF6176 | MAT1-2 | Hemiptera: Cicadelidae | HEM | Tibet | TB | 2013/07/05-13 |
| Bb3185 | RCEF6180 | MAT1-2 | Hemiptera: Pentatomidae | HEM | Tibet | TB | 2013/07/05-31 |
| Bb3187 | RCEF6182 | MAT1-2 | Hymenoptera: Formicidae | HYM | Tibet | TB | 2013/07/05-41 |
| Bb3188 | RCEF6183 | MAT1-2 | Coleoptera: Chrysomelidae | COL | Tibet | TB | 2013/07/05-43 |

| | | | | | | | |
|---|---|---|---|---|---|---|---|
| Bb2787 | RCEF5462 | MAT1-1 | Coleoptera: Carabidae | COL | Xin-Jiang | XJ | 2010/08/31-02 |
| Bb2788 | RCEF5463 | MAT1-2 | Lepidoptera | LEP | Xin-Jiang | XJ | 2010/08/31-05 |
| Bb2789 | RCEF5464 | MAT1-1 | Hymenoptera: Apidae | HYM | Xin-Jiang | XJ | 2010/09/28-05 |
| Bb2791 | RCEF5466 | MAT1-1 | Coleoptera | COL | Xin-Jiang | XJ | 2010/09/28-09 |
| Bb2796 | RCEF5471 | MAT1-1 | Diptera | LEP | Xin-Jiang | XJ | 2010/09/29-15 |
| Bb2800 | RCEF5475 | MAT1-1 | Coleoptera: Chrysomelidae | COL | Xin-Jiang | XJ | 2010/08/26-03 |
| Bb2801 | RCEF5476 | MAT1-2 | Coleoptera: Coccineidae | COL | Xin-Jiang | XJ | 2010/08/26-05 |
| Bb2805 | RCEF5480 | MAT1-2 | Hymenoptera | HYM | Xin-Jiang | XJ | 2010/08/26-15 |
| Bb2978 | RCEF5765 | MAT1-1 | Coleoptera: Chrysomelidae | COL | Yun-Nan | YN | 2011/10/10-1 |
| Bb2987 | RCEF5802 | MAT1-1/2 | Hymenoptera: Formicidae | HYM | Yun-Nan | YN | 2011/10/24-7 |
| Bb2988 | RCEF5803 | MAT1-1 | Coleoptera | COL | Yun-Nan | YN | 2011/10/24-10 |
| Bb2993 | RCEF5808 | MAT1-2 | Coleoptera: Chrysomelidae | COL | Yun-Nan | YN | 2011/10/24-22 |
| Bb2995 | RCEF5810 | MAT1-2 | Coleoptera | COL | Yun-Nan | YN | 2011/10/24-1 |
| Bb2996 | RCEF5811 | MAT1-2 | Hymenoptera: Formicidae | HYM | Yun-Nan | YN | 2011/10/24-3 |
| Bb2998 | RCEF5813 | MAT1-1 | Coleoptera: Chrysomelidae | COL | Yun-Nan | YN | 2011/10/24-10 |
| Bb3002 | RCEF5817 | MAT1-1 | Coleoptera: Curculionidae | COL | Yun-Nan | YN | 2011/10/26-11 |
| Bb3003 | RCEF5818 | MAT1-2 | Hymenoptera: Formicidae | HYM | Yun-Nan | YN | 2011/10/26-12 |
| Bb3004 | RCEF5819 | MAT1-2 | Coleoptera: Chrysomelidae | COL | Yun-Nan | YN | 2011/10/26-13 |
| Bb3009 | RCEF5824 | MAT1-1 | Hemiptera | HEM | Yun-Nan | YN | 2011/10/26-21 |
| Bb3010 | RCEF5825 | MAT1-1 | Orthoptera: Pyrgomorphidae, *Atractomorpha* sp. | ORT | Yun-Nan | YN | 2011/10/26-23 |
