# Supplementary material for: Population genomics and evolution of a fungal pathogen after releasing exotic strains to control insect pests for 20 years
Source: ISME J. 2020 Feb 28;14(6):1422–34. doi: 10.1038/s41396-020-0620-8 (PMC7242398; doi:10.1038/s41396-020-0620-8)
Supplement: Supplementary file 11 — Table S2 [file 41396_2020_620_MOESM11_ESM.pdf]

Table S2. Sequencing information of each isolate.

Strains	RCEF No.	Raw reads	Raw bases	Clean reads	Clean bases	Q20%	Q30%	Depth	Reads mapping %	Assembly (bp)	Scaffold No.	N50 (bp)	N90 (bp)	GC%
Bb3	RCEF0003	28,281,304	4,242,195,600	22,667,482	3,239,196,532	96.66	89.74	58.56	64.54	36,897,126	1,062	232,076	15,951	47.88
Bb4	RCEF0004	37,770,464	5,665,569,600	34,557,936	5,075,623,329	98.25	94.40	124.00	86.94	34,630,796	1,133	198,906	17,061	49.15
Bb7	RCEF0007	26,163,374	3,924,506,100	20,068,906	2,849,082,274	96.33	88.89	71.33	88.20	35,922,647	582	422,552	35,365	48.63
Bb8	RCEF0008	32,440,924	4,866,138,600	25,891,902	3,658,880,897	96.22	87.70	89.22	86.05	36,916,586	1,155	381,872	22,441	49.59
Bb9	RCEF0009	27,215,864	4,082,379,600	22,289,760	3,171,867,062	96.28	87.72	78.27	86.86	37,314,447	1,397	371,258	19,620	49.79
Bb13	RCEF0013	30,521,928	4,578,289,200	26,456,902	3,871,524,857	97.51	92.53	96.82	87.93	35,569,091	755	407,122	28,668	48.57
Bb17	RCEF0017	38,011,996	5,701,799,400	34,197,400	5,005,617,761	97.46	92.61	91.92	65.82	36,942,940	1,315	215,323	12,238	47.94
Bb20	RCEF0225	54,313,326	8,146,998,900	43,608,972	8,146,998,900	97.61	92.99	53.09	31.71	37,159,455	5,226	18,494	2,345	43.77
Bb29	RCEF0229	36,012,636	5,401,895,400	31,318,792	5,401,895,400	97.57	92.55	109.56	84.60	32,367,092	4,903	11,089	2,874	48.25
Bb42	RCEF0232	35,115,850	5,267,377,500	30,485,216	5,267,377,500	96.80	90.85	60.94	49.82	33,115,648	1,005	239,579	23,475	45.64
Bb49	RCEF0235	26,210,202	3,931,530,300	23,179,408	3,931,530,300	98.03	93.66	71.51	76.22	34,702,846	1,631	183,865	12,437	47.05
Bb50	RCEF0236	27,757,232	4,163,584,800	23,558,806	4,163,584,800	96.43	89.74	62.25	65.28	36,869,283	1,019	274,671	18,582	47.32
Bb54	RCEF0237	25,574,848	3,836,227,200	22,213,146	3,836,227,200	97.89	93.48	53.32	59.52	36,890,123	1,214	231,456	14,906	47.1
Bb55	RCEF0238	27,096,934	4,064,540,100	23,385,180	4,064,540,100	97.82	93.34	49.81	53.20	35,173,486	1,253	206,461	15,913	46.47
Bb122	RCEF0303	35,616,174	5,342,426,100	27,555,028	3,885,468,255	96.06	87.30	92.20	83.92	35,689,009	905	268,425	22,091	48.6
Bb124	RCEF0305	31,774,246	4,766,136,900	28,611,872	4,190,408,399	97.98	93.69	108.60	91.08	34,654,536	819	361,821	27,570	49.02
Bb125	RCEF0306	36,246,572	5,436,985,800	32,692,780	4,802,987,508	98.47	95.10	96.29	73.15	36,538,041	1,876	58,394	9,361	48
Bb126	RCEF0307	35,079,070	5,261,860,500	27,282,628	3,894,915,380	96.66	89.86	97.08	87.66	35,943,750	547	359,591	34,858	48.52
Bb142	RCEF0323	34,619,900	5,192,985,000	31,401,668	4,605,576,738	98.10	94.01	92.74	72.22	35,021,089	1,103	279,015	18,400	47.9
Bb143	RCEF0324	31,428,214	4,714,232,100	25,436,452	3,636,488,847	96.61	88.60	65.78	65.42	35,017,664	1,327	254,582	12,521	48.08
Bb145	RCEF0326	33,484,640	5,022,696,000	30,606,878	4,495,198,563	98.17	94.19	108.52	85.20	35,562,059	1,217	226,876	15,140	48.88
Bb146	RCEF0327	31,288,816	4,693,322,400	24,169,274	3,406,645,505	96.24	87.82	59.37	62.86	40,621,811	2,328	178,363	5,653	50
Bb148	RCEF0329	29,605,316	4,440,797,400	23,804,970	3,431,278,849	97.21	91.31	62.05	65.06	33,713,561	669	374,040	33,945	50.75
Bb149	RCEF0330	29,579,524	4,436,928,600	24,698,694	3,558,426,395	97.17	89.99	54.25	55.91	36,726,384	2,045	99,180	7,418	46.26
Bb150	RCEF0331	33,091,568	4,963,735,200	29,819,324	4,372,079,882	98.09	93.96	108.64	87.63	33,965,512	758	284,807	32,136	49.85
Bb158	RCEF0339	36,910,294	5,536,544,100	26,499,670	3,747,895,947	96.13	88.47	42.24	40.48	71,269,139	1,871	376,688	30,518	50.45
Bb162	RCEF0343	29,958,714	4,493,807,100	23,691,312	3,403,502,214	96.74	89.97	63.88	67.03	35,338,432	768	315,144	26,874	47.66
Bb163	RCEF0344	28,298,494	4,244,774,100	23,132,250	3,340,922,794	96.88	90.10	63.61	68.00	35,193,731	808	338,300	22,578	47.48
Bb164	RCEF0345	30,668,174	4,600,226,100	24,060,884	3,447,757,950	96.84	90.20	62.66	64.94	36,972,445	1,028	209,549	17,092	47.89
Bb165	RCEF0346	28,130,628	4,219,594,200	21,301,892	2,980,006,865	95.77	87.18	36.79	46.11	33,782,613	1,251	114,765	17,852	47.55
Bb167	RCEF0348	32,965,326	4,944,798,900	30,045,488	4,408,235,872	98.12	94.06	107.01	85.68	35,389,656	968	331,112	21,512	48.51

Bb168	RCEF0349	40,240,254	6,036,038,100	35,677,338	5,233,175,448	97.85	93.54	26.53	18.19	51,254,656	6,017	117,617	2,276	43.64
Bb169	RCEF0350	90,467,974	13,570,196,100	73,586,788	10,656,830,804	97.05	90.56	186.20	63.58	35,646,659	1,661	116,093	8,511	47.63
Bb174	RCEF0355	26,868,750	4,030,312,500	21,386,228	3,070,640,298	96.91	90.48	73.07	83.92	36,687,899	675	344,161	32,678	48.42
Bb175	RCEF0356	33,133,952	4,970,092,800	29,978,276	4,400,675,113	98.35	94.79	67.62	55.87	33,989,419	2,448	60,248	5,230	46.48
Bb178	RCEF0359	27,199,790	4,079,968,500	24,844,936	3,646,935,190	98.17	94.22	86.26	83.51	36,698,283	14,637	2,868	1,255	48.56
Bb181	RCEF0362	32,411,592	4,861,738,800	29,666,078	4,359,447,684	98.21	94.29	102.83	83.52	34,726,046	1,094	265,711	16,485	49.61
Bb183	RCEF0364	28,654,314	4,298,147,100	21,412,176	3,025,565,028	95.95	87.72	74.04	86.32	35,953,230	499	385,552	51,345	48.44
Bb185	RCEF0366	32,667,288	4,900,093,200	29,664,358	4,350,195,836	98.07	93.95	106.49	86.86	34,731,710	922	263,580	28,619	48.1
Bb205	RCEF0391	28,784,164	4,317,624,600	24,695,808	3,585,725,594	97.29	90.96	67.32	67.58	35,763,086	917	348,262	21,949	47.52
Bb208	RCEF0394	30,367,694	4,555,154,100	25,665,934	3,722,920,636	97.14	90.56	83.71	81.54	35,399,159	1,035	252,255	19,424	48.55
Bb210	RCEF0396	32,166,374	4,824,956,100	26,749,216	3,861,227,298	96.89	89.98	70.95	65.84	35,114,465	990	318,285	17,924	47.61
Bb211	RCEF0397	28,458,782	4,268,817,300	23,034,724	3,315,755,905	97.04	90.78	79.00	83.88	35,875,192	752	239,593	26,272	48.64
Bb212	RCEF0398	26,099,568	3,914,935,200	22,596,636	3,292,853,232	97.74	93.11	65.43	71.05	34,855,649	1,403	210,441	13,135	49.35
Bb215	RCEF0401	32,591,990	4,888,798,500	29,848,220	4,383,208,814	98.19	94.23	90.42	73.55	34,254,986	1,032	316,528	20,219	48.34
Bb216	RCEF0402	30,401,608	4,560,241,200	25,850,494	3,752,178,433	97.08	90.50	57.53	54.78	32,955,172	765	374,124	39,778	47.3
Bb218	RCEF0404	27,238,470	4,085,770,500	22,771,762	3,295,753,082	97.31	91.63	55.82	60.56	37,380,325	1,275	185,380	14,484	46.02
Bb219	RCEF0405	30,973,740	4,646,061,000	25,479,874	3,672,499,206	97.15	91.01	64.46	62.82	36,255,304	2,408	57,527	6,533	46.35
Bb221	RCEF0407	28,367,280	4,255,092,000	24,291,028	3,524,856,371	97.13	90.59	64.75	66.16	35,305,887	1,086	244,843	16,411	48.15
Bb226	RCEF0412	31,020,906	4,653,135,900	27,800,246	4,069,329,620	97.98	93.68	83.98	73.37	34,637,167	998	372,899	17,932	48.29
Bb229	RCEF0415	27,630,694	4,144,604,100	21,658,016	3,089,020,566	96.34	88.77	56.01	65.19	36,499,587	1,321	147,716	12,388	48.06
Bb242	RCEF0471	28,660,772	4,299,115,800	22,567,072	3,202,804,559	96.20	87.99	73.62	81.62	35,667,946	1,031	236,576	18,726	48.61
Bb244	RCEF0473	34,123,882	5,118,582,300	31,024,504	4,552,870,089	98.14	94.09	93.23	72.98	34,886,979	967	453,407	19,180	48.46
Bb245	RCEF0474	37,957,858	5,693,678,700	30,000,786	4,252,596,465	96.31	88.08	71.10	61.02	35,756,869	1,535	127,833	10,791	48.2
Bb248	RCEF0477	28,512,040	4,276,806,000	22,555,848	3,224,450,620	96.37	88.63	75.69	83.19	35,590,940	817	354,271	24,433	48.72
Bb249	RCEF0478	34,690,520	5,203,578,000	28,788,392	4,100,960,852	96.42	88.29	90.74	80.38	38,048,583	1,997	124,266	9,034	49.47
Bb250	RCEF0479	29,677,314	4,451,597,100	23,124,136	3,299,075,860	96.35	88.62	63.02	68.39	35,249,769	871	330,437	20,423	47.97
Bb251	RCEF0480	33,123,288	4,968,493,200	30,016,896	4,401,713,424	98.10	94.00	86.85	71.01	34,655,215	1,091	218,117	16,936	48.61
Bb252	RCEF0481	43,189,836	6,478,475,400	36,872,062	5,291,222,902	96.81	89.23	74.36	53.03	35,297,152	2,505	43,966	5,589	47.9
Bb253	RCEF0482	26,600,858	3,990,128,700	22,159,478	3,149,909,712	96.35	88.14	63.01	71.45	38,553,628	2,242	312,937	5,055	49.75
Bb255	RCEF0484	26,724,832	4,008,724,800	20,536,374	2,918,494,982	96.35	88.85	48.69	60.83	35,195,647	910	244,700	18,790	47.8
Bb257	RCEF0486	31,828,024	4,774,203,600	26,760,896	3,877,758,750	97.11	90.51	72.77	67.47	35,231,917	844	315,261	20,887	47.75
Bb259	RCEF0488	30,881,782	4,632,267,300	26,133,628	3,809,047,717	97.43	91.32	59.53	56.58	35,186,880	965	244,404	18,202	47.05
Bb261	RCEF0490	29,119,816	4,367,972,400	23,131,798	3,303,238,476	96.35	88.79	59.47	64.09	36,363,178	713	433,469	29,209	48
Bb262	RCEF0491	28,562,552	4,284,382,800	25,456,866	3,718,942,923	97.22	92.02	86.60	82.81	34,889,179	1,158	200,854	16,555	49.39
Bb263	RCEF0492	27,158,266	4,073,739,900	22,718,616	3,291,335,424	96.92	90.03	61.46	67.08	35,394,498	819	347,964	24,299	47.81

Bb266	RCEF0495	29,234,934	4,385,240,100	21,552,076	3,008,311,244	95.49	86.50	45.99	56.65	34,765,293	1,785	64,783	8,968	47.91
Bb267	RCEF0496	28,703,618	4,305,542,700	26,071,890	3,824,002,017	97.63	93.18	53.39	51.06	32,934,165	915	199,273	29,848	47.61
Bb268	RCEF0497	30,702,562	4,605,384,300	25,546,014	3,648,486,306	96.29	87.90	66.04	65.07	35,355,079	765	316,264	32,453	47.68
Bb269	RCEF0498	29,592,426	4,438,863,900	24,253,012	3,440,730,563	96.33	88.00	51.10	54.08	36,663,373	3,129	48,027	4,170	45.71
Bb271	RCEF0500	32,664,804	4,899,720,600	26,775,720	3,819,493,681	96.35	88.10	72.97	68.62	35,334,381	978	290,117	17,191	47.88
Bb1982	RCEF3483	32,262,528	4,839,379,200	25,231,886	3,563,151,799	96.48	88.55	81.16	80.48	34,863,979	1,037	251,261	20,313	48.25
Bb1999	RCEF3569	37,927,754	5,689,163,100	29,778,862	4,225,367,283	96.44	88.47	98.35	83.07	35,070,268	1,076	384,770	16,352	49.43
Bb2000	RCEF3570	37,631,520	5,644,728,000	30,319,034	4,313,913,912	96.44	88.40	105.01	85.85	35,755,885	669	338,453	32,013	48.46
Bb2001	RCEF3571	38,261,304	5,739,195,600	31,185,196	4,451,766,054	96.85	89.40	95.62	78.79	35,548,702	1,339	143,067	12,711	48.6
Bb2003	RCEF3573	35,170,278	5,275,541,700	31,250,856	4,571,563,636	97.75	93.36	75.78	60.63	35,864,389	1,331	129,835	12,086	48.08
Bb2006	RCEF3602	34,260,908	5,139,136,200	27,851,728	3,972,095,487	96.61	88.78	101.49	89.98	37,805,135	3,934	35,984	3,085	45.55
Bb2007	RCEF3609	31,052,020	4,657,803,000	28,505,786	4,188,286,586	97.63	92.95	109.28	92.11	33,447,182	775	306,979	38,977	49.09
Bb2009	RCEF3611	28,219,714	4,232,957,100	21,053,166	2,964,546,445	96.30	88.14	72.02	85.66	35,659,714	464	377,211	43,520	47.94
Bb2010	RCEF3612	29,359,922	4,403,988,300	22,074,656	3,111,120,743	96.32	88.14	59.57	68.36	35,591,240	772	489,515	24,696	48.34
Bb2014	RCEF3616	28,991,470	4,348,720,500	22,472,460	3,183,722,017	96.16	87.96	57.47	65.02	33,351,591	532	525,124	43,547	50.5
Bb2016	RCEF3618	30,365,950	4,554,892,500	23,714,334	3,363,375,385	96.71	89.32	62.19	67.02	33,550,731	1,229	240,082	16,243	49.04
Bb2049	RCEF3759	37,217,232	5,582,584,800	27,952,054	3,931,703,969	96.86	89.82	53.10	50.15	32,680,394	756	298,808	35,675	47.53
Bb2050	RCEF3760	35,383,744	5,307,561,600	32,213,150	4,726,379,624	98.12	94.07	92.85	70.26	36,525,886	1,662	145,768	9,693	46.82
Bb2052	RCEF3762	29,668,684	4,450,302,600	23,166,692	3,291,136,617	96.85	89.50	44.33	48.94	35,281,724	1,152	160,847	22,219	45.38
Bb2053	RCEF3763	33,103,434	4,965,515,100	21,178,010	2,909,099,813	95.39	86.30	54.53	67.39	35,686,309	671	417,752	30,178	48.14
Bb2054	RCEF3764	29,378,388	4,406,758,200	22,054,088	3,093,969,504	95.94	87.24	58.93	68.13	34,918,450	566	426,820	44,049	47.92
Bb2056	RCEF3766	33,500,036	5,025,005,400	30,188,560	4,427,124,779	98.08	93.93	108.84	86.88	33,790,171	1,054	207,888	20,582	50.03
Bb2057	RCEF3767	33,556,646	5,033,496,900	26,634,778	3,766,365,865	96.29	87.81	69.29	66.09	37,945,539	1,103	196,308	17,832	45.77
Bb2059	RCEF3769	31,705,190	4,755,778,500	24,085,430	3,407,932,856	96.23	87.63	66.78	70.12	35,577,614	596	410,471	37,197	47.92
Bb2060	RCEF3770	33,011,298	4,951,694,700	27,540,496	3,931,421,401	96.63	88.60	48.74	44.78	34,293,211	1,144	146,995	22,175	45.2
Bb2061	RCEF3771	31,466,044	4,719,906,600	28,322,470	4,151,827,531	98.04	93.86	84.43	72.61	33,401,986	750	295,103	41,916	49.2
Bb2062	RCEF3772	31,464,842	4,719,726,300	26,165,370	3,752,865,994	96.77	88.92	19.56	19.10	33,341,594	789	417,754	41,734	5023
Bb2063	RCEF3773	38,958,280	5,843,742,000	32,133,246	4,578,306,957	96.54	88.43	109.16	86.32	34,829,444	673	213,611	34,802	48.18
Bb2064	RCEF3774	30,894,814	4,634,222,100	26,110,638	3,740,150,311	96.88	89.16	91.94	86.55	35,258,112	328	400,273	73,367	47.85
Bb2068	RCEF3778	28,177,000	4,226,550,000	22,895,912	3,255,659,970	96.57	88.43	60.91	66.75	36,562,948	892	393,807	20,244	48.24
Bb2070	RCEF3780	34,824,310	5,223,646,500	30,948,464	4,543,656,444	98.17	94.24	83.58	65.95	34,900,914	665	362,036	30,660	47.81
Bb2071	RCEF3781	29,858,014	4,478,702,100	25,098,804	3,615,354,097	97.49	90.76	39.67	40.14	32,485,372	1,032	269,110	32,981	46.72
Bb2072	RCEF3782	29,615,248	4,442,287,200	23,902,078	3,403,716,504	96.61	88.78	84.44	87.35	34,671,097	504	334,576	40,282	48
Bb2113	RCEF3920	28,472,654	4,270,898,100	22,782,132	3,229,890,331	96.24	87.65	63.51	70.12	35,125,507	605	325,204	41,151	47.81
Bb2115	RCEF3922	61,372,696	9,205,904,400	54,918,620	8,039,690,479	97.99	93.77	156.83	69.36	37,296,580	1,147	192,251	17,601	45.68

Bb2116	RCEF3923	29,985,796	4,497,869,400	27,320,334	4,009,626,059	97.62	93.02	71.29	63.54	37,509,920	1,268	169,149	13,026	46.03
Bb2117	RCEF3924	29,774,812	4,466,221,800	27,458,374	4,042,707,957	97.89	93.75	64.15	57.15	35,434,080	1,520	129,909	10,841	45.81
Bb2118	RCEF3925	30,600,772	4,590,115,800	24,291,056	3,460,407,462	96.51	88.33	64.63	66.74	37,985,418	1,129	196,146	16,756	45.93
Bb2119	RCEF3926	34,877,794	5,231,669,100	28,142,202	4,028,952,913	96.52	88.33	76.93	68.24	35,315,010	790	364,440	24,653	47.95
Bb2120	RCEF3927	30,986,406	4,647,960,900	27,147,958	3,967,165,466	97.29	92.22	76.26	68.35	36,550,008	1,287	156,790	14,986	46.13
Bb2121	RCEF3928	32,307,102	4,846,065,300	26,354,094	3,770,336,146	96.57	88.39	69.63	65.96	37,857,197	1,195	199,863	15,587	45.93
Bb2123	RCEF3930	29,519,956	4,427,993,400	23,877,422	3,436,323,437	96.91	90.13	59.66	62.15	37,633,262	933	217,355	22,718	45.67
Bb2125	RCEF3932	37,012,362	5,551,854,300	26,820,616	3,805,325,708	96.24	88.70	71.82	67.63	35,303,889	678	453,384	31,661	47.95
Bb2126	RCEF3933	39,900,134	5,985,020,100	29,512,624	4,192,423,817	96.44	89.41	100.28	84.36	35,680,349	444	380,524	45,926	47.45
Bb2131	RCEF4012	30,514,750	4,577,212,500	26,914,396	3,933,832,700	97.30	92.23	68.40	61.93	38,210,780	1,906	117,622	9,165	44.99
Bb2132	RCEF4013	49,796,406	7,469,460,900	37,459,812	5,346,579,716	96.87	90.61	119.06	81.19	35,968,318	1,162	127,619	16,324	47.97
Bb2135	RCEF4016	41,198,400	6,179,760,000	31,796,582	4,536,749,304	96.64	89.89	108.71	84.57	34,667,880	553	549,913	41,898	49.24
Bb2136	RCEF4017	44,402,050	6,660,307,500	31,830,032	4,520,385,445	96.23	89.04	65.67	51.14	68,808,939	1,070	555,909	43,231	51.02
Bb2137	RCEF4018	30,260,638	4,539,095,700	23,850,652	3,388,096,074	96.62	88.99	55.40	58.70	37,423,061	1,118	203,446	17,374	45.95
Bb2138	RCEF4019	27,645,542	4,146,831,300	21,051,286	2,989,335,424	96.36	89.03	67.52	80.01	36,735,790	948	282,884	22,746	48.53
Bb2139	RCEF4020	33,002,684	4,950,402,600	29,766,932	4,355,444,048	97.32	92.25	103.49	83.87	34,996,235	1,225	204,025	14,759	48.78
Bb2147	RCEF4046	31,501,856	4,725,278,400	24,518,028	3,482,440,880	96.88	89.30	47.59	50.38	33,853,305	824	291,537	32,495	47.58
Bb2332	RCEF4649	34,642,746	5,196,411,900	29,761,496	5,196,411,900	96.62	90.25	70.44	59.36	35,538,745	678	368,379	28,551	47.3
Bb2334	RCEF4651	31,512,230	4,726,834,500	26,298,748	4,726,834,500	96.25	89.37	67.78	64.06	35,554,728	837	244,057	32,734	47.04
Bb2336	RCEF4653	34,343,168	5,151,475,200	29,396,092	5,151,475,200	96.73	90.74	57.73	49.24	32,428,870	874	385,861	40,526	47.22
Bb2337	RCEF4654	26,378,510	3,956,776,500	22,185,838	3,956,776,500	97.75	93.09	62.20	69.22	35,172,292	1,191	411,922	15,116	48.3
Bb2338	RCEF4655	29,508,274	4,426,241,100	25,554,578	4,426,241,100	96.85	90.86	42.60	41.69	40,568,730	2,293	90,819	8,110	47.78
Bb2340	RCEF4657	36,176,858	5,426,528,700	31,542,704	5,426,528,700	97.95	93.58	52.49	42.75	35,109,109	2,589	74,212	5,539	47.02
Bb2343	RCEF4660	35,419,884	5,312,982,600	29,621,800	5,312,982,600	97.47	92.52	48.33	40.81	33,003,511	916	264,083	30,306	47.33
Bb2345	RCEF4662	35,074,640	5,261,196,000	29,874,536	5,261,196,000	97.67	92.85	63.28	52.80	32,598,176	985	305,113	29,250	47.66
Bb2348	RCEF4665	32,719,620	4,907,943,000	26,615,532	4,907,943,000	97.42	92.25	60.68	56.49	35,339,281	1,086	278,523	17,277	47.58
Bb2349	RCEF4666	25,752,540	3,862,881,000	21,216,668	3,862,881,000	97.58	92.74	47.92	55.90	37,646,532	917	264,004	21,424	45.97
Bb2461	RCEF4993	33,490,252	5,023,537,800	27,624,782	5,023,537,800	97.35	91.85	95.74	84.58	35,458,576	618	312,793	38,766	48.3
Bb2462	RCEF4994	33,597,998	5,039,699,700	28,071,630	5,039,699,700	97.60	92.68	59.70	53.14	35,039,579	1,195	236,130	16,625	47.93
Bb2464	RCEF4996	36,346,022	5,451,903,300	32,496,722	5,451,903,300	98.37	94.52	70.33	55.20	32,389,852	5,315	10,782	2,438	49.35
Bb2468	RCEF5000	70,008,672	10,501,300,800	50,330,742	10,501,300,800	98.48	95.31	136.62	71.84	34,881,030	4,150	15,433	3,640	48.97
Bb2469	RCEF5001	26,683,638	4,002,545,700	23,446,522	4,002,545,700	97.96	93.64	47.68	51.07	2,741,622	113	105,792	12,893	47.53
Bb2472	RCEF5004	28,488,518	4,273,277,700	25,209,540	4,273,277,700	97.97	93.74	50.84	50.16	34,344,819	1,235	226,028	20,599	46.63
Bb2477	RCEF5009	28,810,376	4,321,556,400	24,980,028	4,321,556,400	97.55	92.48	71.00	70.33	33,537,713	933	198,245	25,841	49.4
Bb2490	RCEF5124	44,852,328	6,727,849,200	36,628,952	6,727,849,200	97.25	91.48	128.97	85.89	32,220,746	5,069	10,384	2,789	47.72

Bb2491	RCEF5125	28,066,788	4,210,018,200	23,820,970	4,210,018,200	96.75	90.44	79.77	82.45	36,295,402	838	296,246	25,746	48.1
Bb2492	RCEF5126	25,666,036	3,849,905,400	20,765,558	3,849,905,400	97.37	92.20	69.70	82.23	27,586,235	832	221,346	16,133	49.04
Bb2493	RCEF5127	29,408,998	4,411,349,700	23,854,590	4,411,349,700	97.81	93.32	28.96	32.04	36,541,004	2,318	61,662	6,589	46.63
Bb2494	RCEF5128	34,600,244	5,190,036,600	30,379,016	5,190,036,600	98.18	94.12	55.47	45.70	33,887,559	1,816	79,844	10,102	46.96
Bb2499	RCEF5133	41,253,794	6,188,069,100	32,854,114	6,188,069,100	98.33	94.47	72.87	55.60	41,919,433	3,094	36,551	6,358	48.78
Bb2501	RCEF5135	51,205,324	7,680,798,600	45,212,972	7,680,798,600	98.05	93.91	90.49	50.17	36,028,570	3,279	33,660	4,276	48.75
Bb2503	RCEF5137	42,872,384	6,430,857,600	33,222,166	6,430,857,600	97.66	92.58	73.17	56.73	36,515,186	1,380	163,072	11,701	47.07
Bb2509	RCEF5143	27,181,914	4,077,287,100	22,245,196	4,077,287,100	97.51	92.57	67.45	76.56	35,225,014	1,353	216,509	13,479	48.14
Bb2513	RCEF5147	36,907,186	5,536,077,900	30,573,626	5,536,077,900	97.61	92.76	78.90	64.13	14,687,713	8,973	1,529	1,067	48.23
Bb2520	RCEF5154	29,042,286	4,356,342,900	23,961,032	4,356,342,900	97.47	92.37	84.40	85.59	34,975,549	866	271,950	25,590	48.29
Bb2521	RCEF5155	34,831,438	5,224,715,700	28,177,072	5,224,715,700	97.41	92.26	75.34	65.64	32,871,970	5,302	10,761	2,614	46.93
Bb2524	RCEF5158	37,328,174	5,599,226,100	30,231,374	5,599,226,100	97.51	92.50	93.24	76.92	37,129,947	1,131	200,150	15,964	48.3
Bb2525	RCEF5159	27,193,642	4,079,046,300	21,858,630	4,079,046,300	97.24	91.80	75.43	84.24	38,433,357	2,682	56,743	5,497	46.76
Bb2526	RCEF5160	26,366,332	3,954,949,800	22,166,704	3,954,949,800	97.60	92.61	79.33	86.57	35,252,673	842	226,262	25,088	48.16
Bb2530	RCEF5164	31,768,176	4,765,226,400	27,010,164	4,765,226,400	98.13	94.37	22.96	21.42	39,482,617	6,151	17,673	1,934	42.39
Bb2544	RCEF5178	28,485,082	4,272,762,300	22,976,536	4,272,762,300	97.31	91.88	77.55	82.35	34,677,648	1,233	272,109	18,212	48.54
Bb2566	RCEF5215	25,969,498	3,895,424,700	22,604,552	3,895,424,700	97.82	93.07	53.87	58.42	39,933,426	1,694	207,002	9,787	46.8
Bb2567	RCEF5216	36,079,336	5,411,900,400	28,542,794	5,411,900,400	97.26	91.85	51.50	45.27	33,415,996	5,830	9,340	2,464	46.3
Bb2574	RCEF5223	39,908,248	5,986,237,200	33,786,448	5,986,237,200	97.66	92.77	93.33	67.52	10,543,811	6,167	1,636	1,076	47.73
Bb2583	RCEF5237	41,836,402	6,275,460,300	35,382,714	6,275,460,300	97.96	93.62	98.91	70.68	32,180,295	8,640	5,465	1,568	47.55
Bb2587	RCEF5241	43,149,906	6,472,485,900	37,560,054	6,472,485,900	98.22	94.42	49.53	34.12	25,854,629	10,060	3,025	1,346	47.68
Bb2589	RCEF5243	25,395,584	3,809,337,600	20,215,230	3,809,337,600	95.73	88.05	67.62	83.03	35,857,227	668	340,863	34,285	48.69
Bb2590	RCEF5244	28,778,102	4,316,715,300	22,304,282	4,316,715,300	97.05	91.14	78.06	85.90	34,852,892	649	316,896	35,585	48.84
Bb2592	RCEF5246	27,316,386	4,097,457,900	22,721,262	4,097,457,900	97.56	92.74	62.92	68.76	34,114,776	1,027	254,513	26,243	47.67
Bb2594	RCEF5248	37,998,690	5,699,803,500	30,595,226	5,699,803,500	97.28	91.95	108.67	86.74	35,338,944	468	367,405	62,960	48.65
Bb2596	RCEF5250	39,591,924	5,938,788,600	32,744,352	5,938,788,600	97.47	92.27	116.88	86.48	32,278,240	5,321	9,805	2,621	48.53
Bb2606	RCEF5260	33,132,436	4,969,865,400	28,241,182	4,969,865,400	97.67	92.76	101.45	86.46	32,069,108	4,396	12,701	3,172	48.59
Bb2609	RCEF5263	29,905,878	4,485,881,700	23,761,974	4,485,881,700	97.17	91.55	68.98	71.54	34,063,087	622	305,966	35,772	49.59
Bb2613	RCEF5275	36,678,576	5,501,786,400	31,021,984	5,501,786,400	97.70	92.83	78.83	62.05	32,220,683	9,182	4,665	1,645	48.38
Bb2615	RCEF5277	30,619,372	4,592,905,800	25,701,232	4,592,905,800	97.30	91.78	92.11	87.02	34,728,224	1,019	303,058	19,321	49.07
Bb2623	RCEF5285	25,425,548	3,813,832,200	20,571,376	3,813,832,200	97.26	91.88	68.34	80.69	35,615,509	910	255,985	19,880	48.76
Bb2629	RCEF5291	30,400,484	4,560,072,600	25,313,660	4,560,072,600	97.61	92.61	55.68	55.24	35,974,233	1,604	184,396	9,208	48.16
Bb2665	RCEF5326	27,527,576	4,129,136,400	24,079,852	4,129,136,400	97.77	92.92	64.41	65.32	37,156,043	1,274	357,877	13,206	48.35
Bb2671	RCEF5332	31,103,660	4,665,549,000	25,426,442	4,665,549,000	96.41	89.89	80.62	79.88	35,080,446	1,038	243,262	17,235	48.24
Bb2693	RCEF5360	34,682,862	5,202,429,300	27,897,804	5,202,429,300	96.59	90.57	79.65	73.88	35,006,307	1,507	85,716	12,900	47.74

Bb2694	RCEF5361	29,944,190	4,491,628,500	26,769,366	4,491,628,500	97.96	93.44	63.24	58.49	39,010,942	1,924	187,552	7,861	46.89
Bb2695	RCEF5362	26,094,784	3,914,217,600	20,820,758	3,914,217,600	96.39	89.84	68.19	82.19	37,494,233	1,804	128,386	8,400	47.41
Bb2696	RCEF5363	27,093,866	4,064,079,900	23,667,696	4,064,079,900	97.88	93.21	62.61	65.11	35,921,479	728	418,515	23,388	48.05
Bb2699	RCEF5366	29,568,266	4,435,239,900	23,463,316	4,435,239,900	96.41	89.87	77.89	82.74	35,913,500	649	386,061	33,488	48.21
Bb2700	RCEF5367	35,635,026	5,345,253,900	27,817,682	5,345,253,900	96.62	90.65	42.34	39.41	33,571,813	988	241,390	24,988	46.35
Bb2701	RCEF5368	27,069,558	4,060,433,700	21,731,740	4,060,433,700	96.37	89.75	69.85	80.38	36,480,815	1,178	264,958	15,930	48.54
Bb2702	RCEF5369	30,276,438	4,541,465,700	24,700,216	4,541,465,700	96.42	89.92	57.35	59.09	34,641,188	998	361,707	18,183	47.67
Bb2703	RCEF5370	31,856,850	4,778,527,500	25,766,010	4,778,527,500	96.38	89.79	87.14	84.09	36,287,272	822	387,890	24,803	48.42
Bb2721	RCEF5388	34,048,508	5,107,276,200	28,173,162	5,107,276,200	96.61	90.33	26.05	23.59	66,424,741	6,182	20,474	4,999	48.8
Bb2723	RCEF5390	27,860,860	4,179,129,000	24,072,486	4,179,129,000	97.95	93.88	56.96	58.76	36,229,306	1,150	261,138	17,574	47.06
Bb2787	RCEF5462	29,370,922	4,405,638,300	25,653,750	4,405,638,300	97.92	93.78	58.62	56.86	36,081,771	1,468	174,359	11,834	47
Bb2788	RCEF5463	34,825,914	5,223,887,100	30,021,958	5,223,887,100	98.26	94.69	41.31	35.74	33,452,152	4,096	16,178	3,397	47.7
Bb2789	RCEF5464	28,963,844	4,344,576,600	25,639,380	4,344,576,600	97.95	93.42	64.88	62.39	34,467,764	1,019	338,525	20,307	47.96
Bb2791	RCEF5466	37,714,758	5,657,213,700	32,309,448	5,657,213,700	97.78	93.42	21.50	16.74	34,792,403	710	455,436	28,698	50.65
Bb2796	RCEF5471	49,246,354	7,386,953,100	40,061,946	7,386,953,100	98.29	94.38	85.74	53.95	39,699,121	1,756	213,239	7,346	47.88
Bb2800	RCEF5475	36,765,360	5,514,804,000	32,494,010	5,514,804,000	98.05	94.14	86.17	65.15	37,230,174	1,258	172,708	13,900	47.83
Bb2801	RCEF5476	36,043,346	5,406,501,900	31,735,150	5,406,501,900	97.64	92.73	82.43	63.39	32,972,997	6,226	8,222	2,307	48.55
Bb2805	RCEF5480	35,971,464	5,395,719,600	30,651,140	5,395,719,600	97.21	91.81	79.31	63.85	36,593,697	1,203	196,493	14,145	48.56
Bb2901	RCEF5657	29,206,360	4,380,954,000	23,912,912	4,380,954,000	97.09	91.12	61.41	63.24	35,927,023	5,244	12,436	2,828	48.32
Bb2903	RCEF5659	30,979,636	4,646,945,400	25,257,074	4,646,945,400	97.09	91.24	90.37	87.20	32,103,711	4,856	11,318	2,827	48.09
Bb2908	RCEF5664	29,099,446	4,364,916,900	25,530,526	4,364,916,900	97.97	93.90	59.83	58.63	35,767,135	1,516	137,928	11,140	48.86
Bb2910	RCEF5666	40,519,540	6,077,931,000	34,314,972	6,077,931,000	97.67	93.23	93.32	67.43	34,234,028	1,175	175,158	14,926	50.29
Bb2915	RCEF5671	32,411,840	4,861,776,000	27,532,184	4,861,776,000	97.89	93.66	17.74	16.24	34,051,727	358	455,521	92,211	50.91
Bb2916	RCEF5672	30,633,882	4,595,082,300	24,628,680	4,595,082,300	97.29	91.64	67.00	67.52	32,273,150	5,039	10,572	2,789	50.03
Bb2924	RCEF5680	33,140,206	4,971,030,900	25,471,582	4,971,030,900	97.26	91.51	53.82	54.26	35,036,494	1,486	157,304	12,628	48.93
Bb2928	RCEF5684	30,108,214	4,516,232,100	25,388,030	4,516,232,100	97.22	91.45	73.95	71.40	31,018,671	15,668	2,093	1,149	49.37
Bb2929	RCEF5685	36,606,808	5,491,021,200	25,157,184	5,491,021,200	97.54	92.30	60.00	61.07	33,149,583	844	225,382	29,228	48.2
Bb2978	RCEF5765	34,523,888	5,178,583,200	28,762,876	5,178,583,200	97.28	91.72	78.66	67.42	32,567,673	5,295	10,324	2,637	48.16
Bb2984	RCEF5771	33,710,762	5,056,614,300	28,483,338	5,056,614,300	97.14	91.53	87.95	77.40	31,827,488	7,534	5,912	1,950	47.32
Bb2987	RCEF5802	32,930,794	4,939,619,100	26,273,742	4,939,619,100	97.49	92.24	8.10	8.31	58,775,967	17,358	4,513	1,538	49.28
Bb2988	RCEF5803	32,601,584	4,890,237,600	27,184,248	4,890,237,600	97.26	91.54	18.07	16.69	33,238,796	2,711	24,353	5,710	51.21
Bb2993	RCEF5808	32,359,628	4,853,944,200	27,544,952	4,853,944,200	97.24	91.38	18.23	16.77	34,922,877	591	712,914	40,698	50.36
Bb2995	RCEF5810	27,805,620	4,170,843,000	22,923,012	4,170,843,000	97.09	91.02	12.92	14.35	34,067,532	474	805,641	75,816	49.67
Bb2996	RCEF5811	28,555,332	4,283,299,800	23,288,588	4,283,299,800	97.10	91.07	14.08	15.41	34,163,601	399	842,662	76,940	49.79
Bb2998	RCEF5813	34,229,136	5,134,370,400	28,985,940	5,134,370,400	97.21	91.28	20.28	17.58	34,711,847	518	761,986	48,014	50.03

Bb3002	RCEF5817	31,499,532	4,724,929,800	26,573,856	4,724,929,800	97.15	91.25	81.43	75.87	34,056,061	940	183,223	24,974	47.49
Bb3003	RCEF5818	37,824,914	5,673,737,100	31,517,220	5,673,737,100	97.75	93.34	106.30	83.91	33,926,517	1,255	148,075	19,489	48.4
Bb3004	RCEF5819	37,600,530	5,640,079,500	29,184,584	5,640,079,500	97.09	91.11	82.88	69.98	31,962,361	4,870	10,849	2,819	48.46
Bb3009	RCEF5824	30,009,708	4,501,456,200	25,250,670	4,501,456,200	97.77	93.36	72.88	71.03	34,609,836	1,063	228,143	18,927	48.78
Bb3010	RCEF5825	45,027,788	6,754,168,200	38,642,682	6,754,168,200	97.87	93.59	128.91	81.39	34,980,331	684	266,983	39,408	47.41
Bb3055	RCEF5886	29,756,220	4,463,433,000	25,963,502	4,463,433,000	98.06	94.13	17.29	16.78	34,159,977	356	560,367	87,008	50.83
Bb3057	RCEF5888	31,020,606	4,653,090,900	26,159,572	4,653,090,900	97.81	93.47	17.07	16.51	33,772,758	360	627,636	100,282	50.58
Bb3059	RCEF5890	31,935,514	4,790,327,100	26,710,804	4,790,327,100	98.00	93.93	14.68	14.17	33,327,504	710	226,149	37,376	50.46
Bb3062	RCEF5893	48,384,448	7,257,667,200	42,728,338	7,257,667,200	98.26	94.54	21.44	12.95	35,299,570	1,447	83,350	13,625	49.76
Bb3063	RCEF5894	33,087,540	4,963,131,000	27,712,788	4,963,131,000	98.10	94.21	10.71	10.06	34,302,462	815	218,778	31,566	49
Bb3070	RCEF5901	34,712,326	5,206,848,900	30,071,584	5,206,848,900	98.13	94.25	19.94	16.90	33,730,976	679	412,776	44,327	50.74
Bb3165	RCEF6160	37,270,574	5,590,586,100	32,666,556	5,590,586,100	98.10	94.16	20.17	15.57	34,163,985	660	402,494	40,527	49.93
Bb3170	RCEF6165	38,789,252	5,818,387,800	31,970,966	5,818,387,800	97.43	92.16	23.00	17.88	35,472,422	394	548,476	62,682	50.35
Bb3179	RCEF6174	31,100,926	4,665,138,900	26,929,760	4,665,138,900	98.03	94.00	17.53	16.52	35,261,182	956	406,108	22,087	50.28
Bb3181	RCEF6176	36,193,406	5,429,010,900	30,491,210	5,429,010,900	97.82	93.51	19.88	16.39	35,563,698	681	541,786	32,586	50.18
Bb3185	RCEF6180	34,723,416	5,208,512,400	29,689,424	5,208,512,400	97.93	93.73	18.31	15.51	35,346,149	849	493,967	26,580	50.24
Bb3187	RCEF6182	53,233,930	7,985,089,500	46,258,850	7,985,089,500	98.16	94.33	109.62	59.35	36,101,692	1,644	129,879	10,074	48.85
Bb3188	RCEF6183	37,346,848	5,602,027,200	30,376,806	5,602,027,200	97.55	92.81	18.48	15.61	35,906,997	754	391,859	25,991	49.48
Bb3214	RCEF6313	30,753,114	4,612,967,100	25,416,348	4,612,967,100	97.50	92.43	79.53	76.94	33,304,167	704	506,150	41,021	48.93
Bb3215	RCEF6314	27,642,048	4,146,307,200	24,667,706	4,146,307,200	98.01	93.58	59.28	59.37	34,519,447	1,122	316,201	16,074	49.33
Bb3218	RCEF6317	43,909,008	6,586,351,200	36,957,322	6,586,351,200	97.57	92.83	71.76	47.73	32,509,644	830	321,308	37,209	46.59
Bb3229	RCEF6365	36,981,980	5,547,297,000	33,428,500	4,935,077,686	98.09	94.27	52.13	38.32	47,356,441	1,742	78,782	15,041	56.49
Bb3230	RCEF6366	55,456,912	8,318,536,800	51,341,712	7,607,061,961	96.58	90.44	137.82	64.43	35,517,650	699	215,423	46,860	48.64
Bb3231	RCEF6367	41,371,358	6,205,703,700	37,441,084	5,529,594,182	98.08	94.14	109.81	71.26	48,491,039	2,235	83,634	9,852	56.13
Bb3232	RCEF6368	47,161,472	7,074,220,800	43,457,476	6,457,169,206	97.13	91.70	118.35	65.22	35,642,563	576	214,984	37,045	48.28
Bb3233	RCEF6369	34,667,822	5,200,173,300	31,992,764	4,730,711,666	98.37	94.80	68.85	53.36	44,808,256	3,825	62,707	3,533	54.83
Bb3235	RCEF6371	72,092,618	10,813,892,700	66,214,394	9,843,033,316	97.23	91.88	185.83	67.14	35,495,767	663	185,695	32,806	48.07
Bb3236	RCEF6372	42,465,216	6,369,782,400	38,619,738	5,697,826,107	98.09	94.02	51.91	32.96	53,502,881	1,470	131,885	19,141	56.46
Bb3237	RCEF6373	38,874,542	5,831,181,300	35,584,328	5,254,524,280	98.19	94.17	13.82	10.21	47,450,720	13,201	4,895	1,490	55.32
Bb3238	RCEF6374	31,021,326	4,653,198,900	27,808,908	4,086,143,598	98.18	94.31	9.54	9.48	22,388,266	14,632	1,504	1,079	59.43
Bb3239	RCEF6375	43,673,362	6,551,004,300	39,557,286	5,835,818,962	98.12	94.11	90.18	55.67	51,344,787	4,248	62,376	4,068	55.97
Bb3240	RCEF6376	61,401,588	9,210,238,200	56,755,022	8,434,338,939	97.21	91.86	160.72	67.85	35,283,772	647	148,402	35,811	48.45
Bb3241	RCEF6377	47,783,292	7,167,493,800	39,234,816	5,778,734,749	95.89	88.98	109.56	67.38	35,252,704	653	171,538	33,499	48.48
Bb3242	RCEF6378	61,361,602	9,204,240,300	55,724,792	8,279,032,710	97.08	91.58	159.56	68.51	35,240,640	639	143,141	37,373	48.53
Bb3243	RCEF6379	37,459,472	5,618,920,800	33,891,084	4,986,460,953	97.97	93.55	20.55	15.21	52,985,076	3,992	51,867	4,694	56.36

Bb3244	RCEF6380	37,147,424	5,572,113,600	34,444,898	5,096,427,063	98.32	94.67	90.56	63.69	37,812,587	3,691	100,261	2,451	52.25
Bb3245	RCEF6381	37,468,520	5,620,278,000	33,283,356	4,898,499,098	98.13	94.01	19.45	15.04	44,978,380	11,149	6,135	1,650	56.74
Bb3246	RCEF6382	42,217,954	6,332,693,100	35,485,686	5,227,511,451	95.78	88.75	100.87	68.56	34,694,040	814	167,931	24,534	48.71
Bb3247	RCEF6383	32,353,608	4,853,041,200	25,446,804	3,691,998,304	98.05	93.94	3.22	3.85	2,320,700	1,912	1,193	1,029	51.14
Bb3248	RCEF6384	35,666,898	5,350,034,700	29,959,048	4,406,608,234	98.37	94.83	12.84	11.73	3,806,421	3,071	1,208	1,029	55.25
Bb3249	RCEF6385	36,023,354	5,403,503,100	31,570,164	4,650,348,403	98.18	94.27	33.36	28.10	50,436,812	11,531	7,052	1,720	55.53
Bb3250	RCEF6386	37,545,496	5,631,824,400	32,535,134	4,731,140,980	97.92	93.65	11.37	9.01	41,392,096	16,212	2,769	1,252	57.22
Bb3251	RCEF6387	38,957,928	5,843,689,200	33,933,834	4,991,282,683	97.97	93.56	75.82	54.55	49,129,324	2,380	143,879	11,619	56.69
Bb3252	RCEF6388	46,034,250	6,905,137,500	41,578,066	6,127,226,131	98.16	93.81	3.28	2.01	21,491,618	5,510	10,744	1,355	57.42
Bb3253	RCEF6389	34,810,602	5,221,590,300	28,036,918	4,092,094,246	94.62	86.47	88.50	77.05	33,973,797	2,654	32,189	5,527	50.84
Bb3254	RCEF6390	44,044,184	6,606,627,600	39,395,600	5,794,595,344	98.16	93.88	12.38	7.94	51,585,417	13,530	5,081	1,667	55.26
Bb3257	RCEF6393	35,946,258	5,391,938,700	31,792,366	4,686,465,011	98.12	93.96	87.53	67.39	50,680,505	4,107	116,334	3,649	55.33
Bb3258	RCEF6394	44,919,814	6,737,972,100	40,410,534	5,944,367,243	98.17	94.03	22.32	13.96	51,537,377	5,768	35,811	2,689	56.43
Bb3259	RCEF6395	36,433,538	5,465,030,700	23,778,390	3,486,556,363	98.07	94.03	55.36	59.32	11,992,584	8,380	1,406	1,064	54.41
Bb3260	RCEF6396	34,021,746	5,103,261,900	27,056,782	3,964,865,602	95.28	87.71	72.87	65.32	34,072,582	3,362	23,888	4,086	49.68
Bb3261	RCEF6397	34,725,156	5,208,773,400	30,011,974	4,418,835,597	98.01	93.69	84.27	71.80	45,814,584	5,204	26,607	3,105	52.74
Bb3262	RCEF6398	36,003,994	5,400,599,100	26,954,546	3,934,501,486	97.71	93.27	64.44	58.70	37,192,093	2,049	51,900	8,042	48.75
Bb3263	RCEF6399	38,017,360	5,702,604,000	33,164,774	4,883,627,327	98.28	94.66	121.02	87.09	33,960,609	1,024	179,051	24,266	50.75
Bb3264	RCEF6400	36,296,752	5,444,512,800	32,247,606	4,759,498,955	98.18	94.18	67.63	52.73	39,924,985	4,500	64,847	2,328	51.91
Bb3265	RCEF6401	40,690,712	6,103,606,800	34,454,212	5,061,893,092	98.10	94.16	89.71	63.10	36,955,347	889	191,925	25,877	48.76
Bb3266	RCEF6402	25,378,212	3,806,731,800	20,821,870	3,063,818,608	95.89	88.99	50.56	58.82	37,123,342	1,355	96,066	14,420	48.43
Bb3267	RCEF6403	41,048,622	6,157,293,300	18,678,638	2,743,102,786	97.88	93.44	53.60	72.75	11,931,306	7,210	1,621	1,087	58.31
Bb3268	RCEF6404	35,673,796	5,351,069,400	32,013,786	4,701,717,943	98.24	94.45	47.29	36.78	52,678,249	3,453	89,103	5,563	56.2
Bb3269	RCEF6405	38,090,136	5,713,520,400	34,746,006	5,140,350,682	98.20	94.27	93.14	64.53	35,351,360	742	317,857	25,760	48.35
Bb3270	RCEF6406	34,355,288	5,153,293,200	31,037,340	4,588,322,333	98.13	93.97	88.61	68.60	34,928,990	1,450	578,245	23,993	51.61
Bb3271	RCEF6407	33,812,924	5,071,938,600	30,292,682	4,475,974,434	98.09	93.88	81.74	65.48	37,886,788	2,789	272,814	3,423	53.12
Bb3272	RCEF6408	40,653,472	6,098,020,800	32,482,660	4,769,237,488	95.09	87.19	13.88	10.42	33,440,728	4,683	12,025	3,128	49.7
Bb3273	RCEF6409	36,785,564	5,517,834,600	33,585,518	4,971,651,127	98.23	94.25	97.92	70.24	34,820,259	1,069	364,437	17,077	49.33
Bb3274	RCEF6410	38,140,618	5,721,092,700	34,672,522	5,129,937,066	98.21	94.22	97.04	67.41	33,502,818	470	542,045	59,947	50.74
