# Supplementary material for: Population genomics and evolution of a fungal pathogen after releasing exotic strains to control insect pests for 20 years
Source: ISME J. 2020 Feb 28;14(6):1422–34. doi: 10.1038/s41396-020-0620-8 (PMC7242398; doi:10.1038/s41396-020-0620-8)
Supplement: Supplementary file 12 — Table S3 [file 41396_2020_620_MOESM12_ESM.pdf]

Table S3. Previously genome-sequenced strains of *Beauveria bassiana* included in this study.

**Strains**	**NCBI access**	**Mating type**	**Host classification**	**Geographic origin**	**Collection date (y/m/d)**
ARSEF 4305	JTCY00000000	MAT1-2	Soil	Australia: Taania	1994/06/6
ARSEF 8028	JRHA00000000	MAT1-2	Hemiptera: Anthocoridae, *Anthocoris nemorum*	Denmark: Zealand	2002/06/25
ARSEF 1520	JTCW00000000	MAT1-2	Hemiptera: Miridae, *Lygus* sp.	France	1984/06/27
ARSEF 2597	JTCX00000000	MAT1-1	Lepidoptera: Hyblaeidae, *Hyblaea puer*	India	1988/09/12
D1-5	ANFO00000000	MAT1-1	Lepidoptera: Crambidae, *Ostrinia furnacalis*	China: Jilin	Not available
ARSEF 2860	ADAH00000000	MAT1-1	Hemiptera: Aphididae, *Schizaphis graminum*	USA: Idaho	1987/07/17
ARSEF 5078	JTCZ00000000	MAT1-1	Lepidoptera: Pyralidae	USA: Washington	1994/01/17
