# Supplementary material for: Population genomics and evolution of a fungal pathogen after releasing exotic strains to control insect pests for 20 years
Source: ISME J. 2020 Feb 28;14(6):1422–34. doi: 10.1038/s41396-020-0620-8 (PMC7242398; doi:10.1038/s41396-020-0620-8)
Supplement: Supplementary file 13 — Table S4 [file 41396_2020_620_MOESM13_ESM.pdf]

Table S4. Statistics of the mating-type and host origins for the isolates included in this study.

**Statistic**	**Anhui population**						**All collected isolates**
	1997	2007	2017	G1 lineage	G2 lineage	Total	
Sample size	62	47	43	46	99	152	277
MAT1-1 (#)	35	28	14	18	55	77	133
MAT1-2 (#)	24	18	29	28	41	71	136
MAT1-1/2 (#)	3	1	0	1	3	4	8
Insect host order (#)	6	5	6	5	6	6	6
Colepteran	28	18	28	18	51	74	131
Dipteran	1	3	1	0	5	5	11
Hemipteran	9	11	3	4	18	23	53
Hymenopteran	5	4	5	4	10	14	32
Lepidopteran	16	11	5	20	13	32	52
Orthopteran	3	0	1	1	2	4	9
