# Supplementary material for: Population genomics and evolution of a fungal pathogen after releasing exotic strains to control insect pests for 20 years
Source: ISME J. 2020 Feb 28;14(6):1422–34. doi: 10.1038/s41396-020-0620-8 (PMC7242398; doi:10.1038/s41396-020-0620-8)
Supplement: Supplementary file 14 — Table S5 [file 41396_2020_620_MOESM14_ESM.pdf]

Table S5. Statistics and features of the clonal groups identified within the Anhui population of *Beauveria bass*

**Clonal group**	**Isolate no.**	**MAT**	**Collected year**	**Collected season***	**Orignal insect host order**
C1	2	MAT1-2	2007/2017	SU×2	LEP/HEM
C2	3	MAT1-1	2017	AU×3	COL/ORT×2
C3	2	MAT1-1	2007	SP/AU	COL/LEP
C4	2	MAT1-2	2007/2017	SU×2	COL×2
C5	2	MAT1-1	1997	SP×2	LEP/HEM
C6	2	MAT1-1	2007	SU×2	LEP×2
C7	8	MAT1-1	1997×4/2007×4	SU×3/AU×2/WI×3	COL×3/LEP×4/HYM
C8	11	MAT1-2	1997×10/2007	SP×3/SU/AU/WI×6	COL×3/LEP×6/HEM/HYM
C9	2	MAT1-1	1997/2007	SP/SU	COL/LEP
C10	3	MAT1-2	2007	SU×3	COL×2/HEM
C11	2	MAT1-2	1997	SP/WI	COL/ORT
C12	4	MAT1-2	2017	SU×4	COL/LEP/HEM/HYM
C13	5	MAT1-1	2007	SU×4/AU	COL×3/HEM×2
C14	8	MAT1-1	1997/2007×7	SU×8	HEM×8
C15	5	MAT1-2	1997×4/2017	SP×2/SU×2/WI	COL×3/HEM/LEP
C16	2	MAT1-1	1997	SP/SU	COL/HEM
C17	8	MAT1-1	1997	SP/SU/WI×6	COL×4/LEP/HEM×2/DIP
C18	10	MAT1-1	1997	SP×3/SU×4/WI×3	COL×6/LEP/HEM/HYM/ORT
C19	2	MAT1-2	2017	SU×2	DIP/HYM
C20	2	MAT1-2	1997/2007	SU/WI	COL/LEP
C21	9	MAT1-1	1997×2/2007×5/2017×	SU×4/AU×3/WI×2	COL×6/LEP×3
C22	3	MAT1-2	2017	SU×3	COL×3
C23	3	MAT1-1	2017	SU×3	COL×3
C24	2	MAT1-2	2017	SU/AU	COL×2

*Season: SP for Spring (Mar. 9- May 20); SU, Summar (May 21- Sept. 18); AU, Autumnn (Sept. 19-Nov. 26); WI, Winter (Nov. 27-next Mar. 8).

iana .
