# Supplementary material for: Population genomics and evolution of a fungal pathogen after releasing exotic strains to control insect pests for 20 years
Source: ISME J. 2020 Feb 28;14(6):1422–34. doi: 10.1038/s41396-020-0620-8 (PMC7242398; doi:10.1038/s41396-020-0620-8)
Supplement: Supplementary file 15 — Table S6 [file 41396_2020_620_MOESM15_ESM.pdf]

Table S6. Calculation of the average nucleotide identity (ANI, %) between the released strain Bb13-related isolates.

	**Bb13**	Bb126	Bb150	Bb167	Bb183	Bb208	Bb2132	Bb248	Bb4	Bb7	Bb8	Bb9
Bb126	99.8746	100										
Bb150	99.901	99.902	100									
Bb167	99.8807	99.889	99.902	100								
Bb183	99.8834	99.91	99.897	99.8735	100							
Bb208	99.8766	99.875	99.877	99.8694	99.8668	100						
Bb2132	99.8633	99.87	99.89	99.872	99.8749	99.8457	100					
Bb248	99.869	99.893	99.894	99.8798	99.8579	99.8956	99.8571	100				
Bb4	99.8697	99.879	99.88	99.8725	99.8641	99.8637	99.854	99.8763	100			
Bb7	99.8822	99.906	99.902	99.8782	99.9002	99.8785	99.8708	99.8935	99.8757	100		
Bb8	99.8793	99.906	99.899	99.8731	99.8968	99.8736	99.8699	99.883	99.8654	99.8931	100	
Bb9	99.8812	99.894	99.899	99.8766	99.8834	99.872	99.8704	99.8803	99.8584	99.9062	99.885	100
