# Supplementary material for: Population genomics and evolution of a fungal pathogen after releasing exotic strains to control insect pests for 20 years
Source: ISME J. 2020 Feb 28;14(6):1422–34. doi: 10.1038/s41396-020-0620-8 (PMC7242398; doi:10.1038/s41396-020-0620-8)
Supplement: Supplementary file 16 — Table S7 [file 41396_2020_620_MOESM16_ESM.pdf]

Table S7. Calculation of the average nucleotide identity (ANI, %) between the released strain Bb17-related isolates.

	**Bb17**	Bb3	Bb146	Bb164	Bb212	Bb3266
Bb3	99.8335	100				
Bb146	99.8	99.8194	100			
Bb164	99.8392	99.9019	99.8184	100		
Bb212	99.8719	99.8547	99.8375	99.8594	100	
Bb3266	99.7758	99.7726	99.7972	99.7776	99.7888	100
