# Supplementary material for: Population genomics and evolution of a fungal pathogen after releasing exotic strains to control insect pests for 20 years
Source: ISME J. 2020 Feb 28;14(6):1422–34. doi: 10.1038/s41396-020-0620-8 (PMC7242398; doi:10.1038/s41396-020-0620-8)
Supplement: Supplementary file 17 — Table S8 [file 41396_2020_620_MOESM17_ESM.pdf]

Table S8. Virulence assays against different insects after topical infection with the selected isolates.

Insects	Strains	Mating type	Original host	LT_{50} (hrs)	Bb13		Bb17		Bb269		Bb3241	
					$\chi 2$	*P* value	$\chi 2$	*P* value	$\chi 2$	*P* value	$\chi 2$	*P* value
Drosophila melanogaster	Bb13	MAT1-2	Lepidoptera: Lasiocampidae, *Dendrolimus punctatus*	84±1.14								
	Bb17	MAT1-2	Lepidoptera: Lasiocampidae, *Dendrolimus punctatus*	252±7.19	224.12	1.14E-50						
	Bb269	MAT1-1	Orthoptera: Tettigoniidae	156±3.16	231.89	2.31E-52	136.31	1.70E-31				
	Bb3241	MAT1-2	Diptera: Celyphidae	156±3.16	207.97	3.80E-47	150.90	1.10E-34	5.04	0.02		
	ARSEF 8028	MAT1-2	Hemiptera: Anthocoridae, *Anthocoris nemorum*	132±1.59	146.60	9.58E-34	165.47	7.22E-38	43.96	3.34E-11	23.24	1.43E-06
Galleria mellonella	Bb13	MAT1-2	Lepidoptera: Lasiocampidae, *Dendrolimus punctatus*	108±3.99								
	Bb17	MAT1-2	Lepidoptera: Lasiocampidae, *Dendrolimus punctatus*	144±5.14	35.35	2.75E-09						
	Bb269	MAT1-1	Orthoptera: Tettigoniidae	156±3.94	54.19	1.82E-13	1.58	0.21				
	Bb3241	MAT1-2	Diptera: Celyphidae	156±3.72	37.77	7.94E-10	0.29	0.59	3.83	0.05		
	ARSEF 8028	MAT1-2	Hemiptera: Anthocoridae, *Anthocoris nemorum*	108±1.51	12.82	3.44E-04	68.94	1.01E-16	74.54	5.93E-18	64.53	9.53E-16
Tribolium castaneum	Bb13	MAT1-2	Lepidoptera: Lasiocampidae, *Dendrolimus punctatus*	107±2.47								
	Bb17	MAT1-2	Lepidoptera: Lasiocampidae, *Dendrolimus punctatus*	NA								
	Bb269	MAT1-1	Orthoptera: Tettigoniidae	106±2.11	0.046	0.83						
	Bb3241	MAT1-2	Diptera: Celyphidae	134±4.05	30.73	2.97E-08	NA		32.64	1.11E-08		
	ARSEF 8028	MAT1-2	Hemiptera: Anthocoridae, *Anthocoris nemorum*	94±1.72	19.64	9.00E-06	NA		20.78	5.00E-06	64.65	8.94E-16
