# Supplementary material for: Population genomics and evolution of a fungal pathogen after releasing exotic strains to control insect pests for 20 years
Source: ISME J. 2020 Feb 28;14(6):1422–34. doi: 10.1038/s41396-020-0620-8 (PMC7242398; doi:10.1038/s41396-020-0620-8)
Supplement: Supplementary file 18 — Table S9 [file 41396_2020_620_MOESM18_ESM.pdf]

Table S9. Analysis of the putative effector genes identified with significant selective signatures (top 5% in composite likelihood ratio test) in each population.

**1997 population**		
**Gene ID**	**Annotation**	**Probability***
PQK07921.1	Putative effector	0.571
PQK08001.1	Putative effector	0.654
PQK09679.1	Putative effector	0.595
PQK10125.1	Putative effector	0.848
PQK10249.1	Putative effector	0.56
PQK11371.1	Putative effector	0.621
PQK12965.1	Putative effector	0.817
PQK13105.1	Putative effector	0.727
PQK13230.1	Putative effector	0.723
PQK13756.1	Putative effector	0.638
PQK14447.1	Putative effector	0.781
PQK14483.1	Putative effector	0.688
PQK14484.1	Putative effector	0.611
PQK16844.1	Putative effector	0.732
PQK16862.1	Putative effector	0.728
PQK17161.1	Putative effector	0.598
PQK17162.1	Putative effector	0.604
PQK18051.1	Putative effector	0.593
PQK07922.1	Hypothetical protein	
PQK07964.1	Hypothetical protein	
PQK07968.1	Hypothetical protein	
PQK07995.1	Hypothetical protein	
PQK09101.1	Hypothetical protein	
PQK09627.1	Hypothetical protein	
PQK09637.1	Hypothetical protein	
PQK09694.1	Hypothetical protein	
PQK09811.1	Hypothetical protein	
PQK10241.1	Hypothetical protein	
PQK10242.1	Hypothetical protein	
PQK10252.1	Hypothetical protein	
PQK11342.1	Hypothetical protein	
PQK11425.1	Hypothetical protein	
PQK11429.1	Hypothetical protein	
PQK11671.1	Hypothetical protein	
PQK12984.1	Hypothetical protein	
PQK12989.1	Hypothetical protein	
PQK13089.1	Hypothetical protein	
PQK13232.1	Hypothetical protein	
PQK13809.1	Hypothetical protein	
PQK14125.1	Hypothetical protein	
PQK14416.1	Hypothetical protein	
PQK14548.1	Hypothetical protein	
PQK14876.1	Hypothetical protein	
PQK15430.1	Hypothetical protein	
PQK15471.1	Hypothetical protein	
PQK15630.1	Hypothetical protein	
PQK15631.1	Hypothetical protein	
PQK15632.1	Hypothetical protein	
PQK15804.1	Hypothetical protein	
PQK16328.1	Hypothetical protein	
PQK16329.1	Hypothetical protein	
PQK16774.1	Hypothetical protein	

PQK16798.1	Hypothetical protein	
PQK16800.1	Hypothetical protein	
PQK16841.1	Hypothetical protein	
PQK16849.1	Hypothetical protein	
PQK16850.1	Hypothetical protein	
PQK16851.1	Hypothetical protein	
PQK16856.1	Hypothetical protein	
PQK16860.1	Hypothetical protein	
PQK17439.1	Hypothetical protein	
PQK17993.1	Hypothetical protein	
PQK17994.1	Hypothetical protein	
PQK18002.1	Hypothetical protein	
PQK18008.1	Hypothetical protein	
PQK18052.1	Hypothetical protein	
PQK14906.1	IPR000420(Yeast PIR protein repeat)	2.40E-05
PQK18004.1	IPR000477(Reverse transcriptase domain)	8.20E-29
PQK18006.1	IPR000477(Reverse transcriptase domain)	3.70E-40
PQK09804.1	IPR000560(Histidine phosphatase superfamily, clade-2)	6.00E-110
PQK13670.1	IPR001138(Zn(2)-C6 fungal-type DNA-binding domain)	1.63E-11
PQK17160.1	IPR001144(Heat-labile enterotoxin, A chain)	1.90E-46
PQK09986.1	IPR001214(SET domain)	5.60E-10
PQK16831.1	IPR001214(SET domain)	5.80E-09
PQK12982.1	IPR001401(Dynamin, GTPase domain)	5.62E-91
PQK13749.1	IPR001401(Dynamin, GTPase domain)	5.61E-80
PQK16767.1	IPR001401(Dynamin, GTPase domain)	3.27E-99
PQK16855.1	IPR001401(Dynamin, GTPase domain)	6.14E-90
PQK09815.1	IPR001452(SH3 domain)	1.20E-08
PQK15678.1	IPR002654(Glycosyl transferase, family 25)	1.79E-15
PQK16330.1	IPR002889(Carbohydrate-binding WSC)	1.40E-13
PQK15833.1	IPR003663(Sugar/inositol transporter)	7.70E-114
PQK10143.1	IPR004298(Nicotianamine synthase)	3.50E-25
PQK12978.1	IPR004827(Basic-leucine zipper domain)	2.90E-08
PQK14893.1	IPR005062(SAC3/GANP/THP3)	5.30E-88
PQK09813.1	IPR005198(Glycoside hydrolase, family 76)	8.90E-46
PQK07939.1	IPR005607(BSD domain)	2.70E-20
PQK13672.1	IPR005828(Major facilitator, sugar transporter-like)	2.70E-89
PQK10148.1	IPR006274(Carbamoyl-phosphate synthase, small subunit)	4.50E-134
PQK10149.1	IPR006275(Carbamoyl-phosphate synthase, large subunit)	0
PQK11628.1	IPR006539(P-type ATPase, subfamily IV)	1.00E-153
PQK09814.1	IPR006813(Glycosyl transferase, family 17)	2.20E-43
PQK10020.1	IPR007219(Transcription factor domain, fungi)	1.70E-19
PQK11428.1	IPR007219(Transcription factor domain, fungi)	1.30E-19
PQK11566.1	IPR007219(Transcription factor domain, fungi)	1.60E-12
PQK14887.1	IPR007219(Transcription factor domain, fungi)	9.00E-33
PQK14892.1	IPR007307(Low temperature viability protein Ltv1)	1.50E-125
PQK15680.1	IPR007484(Peptidase M28)	6.30E-25
PQK12361.1	IPR008422(Homeobox KN domain)	4.70E-17
PQK15129.1	IPR008427(Extracellular membrane protein, CFEM domain)	1.00E-05
PQK16043.1	IPR008427(Extracellular membrane protein, CFEM domain)	9.20E-09
PQK11573.1	IPR008631(Glycogen synthase)	0
PQK07923.1	IPR008949(Isoprenoid synthase domain superfamily)	2.40E-43
PQK08512.1	IPR009003(Peptidase S1, PA clan)	8.22E-19
PQK15075.1	IPR009003(Peptidase S1, PA clan)	4.88E-42
PQK10164.1	IPR009057(Homeobox-like domain superfamily)	2.14E-16
PQK16818.1	IPR009057(Homeobox-like domain superfamily)	1.15E-16
PQK12974.1	IPR009291(Vacuolar protein sorting-associated protein 62)	1.40E-09
PQK07963.1	IPR010730(Heterokaryon incompatibility)	6.00E-24

PQK10381.1	IPR010730(Heterokaryon incompatibility)	3.00E-19
PQK16833.1	IPR010730(Heterokaryon incompatibility)	9.20E-38
PQK08977.1	IPR011009(Protein kinase-like domain superfamily)	1.30E-23
PQK09127.1	IPR011009(Protein kinase-like domain superfamily)	2.42E-07
PQK09955.1	IPR011009(Protein kinase-like domain superfamily)	1.57E-36
PQK10245.1	IPR011009(Protein kinase-like domain superfamily)	2.78E-16
PQK15805.1	IPR011009(Protein kinase-like domain superfamily)	4.27E-22
PQK16770.1	IPR011009(Protein kinase-like domain superfamily)	1.80E-05
PQK07933.1	IPR011022(Arrestin C-terminal-like domain)	1.20E-24
PQK08527.1	IPR011141(Polyketide synthase, type III)	6.20E-72
PQK11565.1	IPR011989(Armadillo-like helical)	0
PQK07924.1	IPR011990(Tetratricopeptide-like helical domain superfamily)	5.70E-08
PQK13090.1	IPR012110(Thiamine pyrophosphate (TPP)-dependent enzyme)	3.00E-179
PQK12985.1	IPR012171(Fatty acid desaturase)	3.60E-161
PQK11669.1	IPR012334(Pectin lyase fold)	8.00E-122
PQK15635.1	IPR012337(Ribonuclease H-like superfamily)	3.41E-26
PQK16834.1	IPR012337(Ribonuclease H-like superfamily)	6.32E-19
PQK07927.1	IPR012419(Cas1p 10 TM acyl transferase domain)	9.80E-119
PQK09610.1	IPR012719(T-complex protein 1, gamma subunit)	0
PQK17991.1	IPR013087(Zinc finger C2H2-type)	1.3
PQK13180.1	IPR013320(Concanavalin A-like lectin/glucanase domain superfamily)	4.62E-25
PQK09589.1	IPR013530(Protein-arginine deiminase, C-terminal)	2.60E-13
PQK15636.1	IPR013530(Protein-arginine deiminase, C-terminal)	2.00E-112
PQK10126.1	IPR013785(Aldolase-type TIM barrel)	6.50E-130
PQK11571.1	IPR013857(NADH:ubiquinone oxidoreductase intermediate-associated protein 30)	1.50E-37
PQK11657.1	IPR013966(DASH complex subunit Spc34)	1.20E-23
PQK09810.1	IPR014381(DNA-directed RNA polymerase RPB5 subunit, eukaryote/virus)	1.30E-72
PQK16772.1	IPR015421(Pyridoxal phosphate-dependent transferase, major domain)	9.40E-70
PQK07989.1	IPR016024(Armadillo-type fold)	1.25E-75
PQK11557.1	IPR016084(Haem oxygenase-like, multi-helical)	8.60E-11
PQK11569.1	IPR017230(Rab geranylgeranyltransferase complex component A)	1.90E-134
PQK09212.1	IPR019626(Stress-induced protein, KGG, repeat)	9.60E-12
PQK08526.1	IPR020841(Polyketide synthase, beta-ketoacyl synthase domain)	9.70E-165
PQK10154.1	IPR021109(Aspartic peptidase domain superfamily)	4.98E-24
PQK17992.1	IPR021711(Protein of unknown function DUF3295)	2.20E-39
PQK09693.1	IPR021765(Mycotoxin biosynthesis protein UstYa-like)	2.60E-48
PQK10238.1	IPR021842(Protein of unknown function DUF3435)	3.70E-52
PQK12981.1	IPR021842(Protein of unknown function DUF3435)	1.60E-15
PQK15629.1	IPR021842(Protein of unknown function DUF3435)	2.10E-60
PQK16859.1	IPR022099(Protein of unknown function DUF3638)	1.50E-46
PQK16854.1	IPR022190(Protein of unknown function DUF3716)	4.10E-07
PQK10244.1	IPR022198(Protein of unknown function DUF3723)	1.90E-163
PQK18003.1	IPR022198(Protein of unknown function DUF3723)	9.40E-172
PQK12980.1	IPR023395(Mitochondrial carrier domain superfamily)	8.00E-31
PQK16842.1	IPR023606(CoA-transferase family III domain superfamily)	1.83E-51
PQK14995.1	IPR025122(Domain of unknown function DUF4048)	1.80E-08
PQK14996.1	IPR025444(Monooxygenase af470-like)	3.70E-26
PQK11570.1	IPR027408(PNPase/RNase PH domain superfamily)	1.70E-88
PQK07953.1	IPR027417(P-loop containing nucleoside triphosphate hydrolase)	2.55E-45
PQK10140.1	IPR027417(P-loop containing nucleoside triphosphate hydrolase)	4.17E-61
PQK13257.1	IPR027417(P-loop containing nucleoside triphosphate hydrolase)	4.19E-52
PQK13382.1	IPR027417(P-loop containing nucleoside triphosphate hydrolase)	1.33E-63
PQK13955.1	IPR027417(P-loop containing nucleoside triphosphate hydrolase)	2.56E-39
PQK14891.1	IPR027417(P-loop containing nucleoside triphosphate hydrolase)	3.50E-36
PQK14994.1	IPR027417(P-loop containing nucleoside triphosphate hydrolase)	5.67E-42
PQK11556.1	IPR027443(Isopenicillin N synthase-like)	6.00E-32

PQK18049.1	IPR027796(OTT_1508-like deaminase)	2.10E-18
PQK11579.1	IPR029057(Phosphoribosyltransferase-like)	2.70E-20
PQK09160.1	IPR029058(Alpha/Beta hydrolase fold)	7.30E-40
PQK16769.1	IPR029058(Alpha/Beta hydrolase fold)	1.28E-13
PQK15681.1	IPR029062(Class I glutamine amidotransferase-like)	8.30E-63
PQK09806.1	IPR029063(S-adenosyl-L-methionine-dependent methyltransferase)	9.12E-54
PQK08004.1	IPR029069(HotDog domain superfamily)	3.82E-10
PQK11044.1	IPR032675(Leucine-rich repeat domain superfamily)	1.10E-06
PQK15628.1	IPR032675(Leucine-rich repeat domain superfamily)	1.50E-22
PQK08244.1	IPR034187(PoSI-like peptidase domain)	6.80E-109
PQK13581.1	IPR034187(PoSI-like peptidase domain)	8.46E-116
PQK07955.1	IPR036188(FAD/NAD(P)-binding domain superfamily)	6.04E-43
PQK09614.1	IPR036188(FAD/NAD(P)-binding domain superfamily)	9.00E-94
PQK10142.1	IPR036188(FAD/NAD(P)-binding domain superfamily)	1.40E-60
PQK16773.1	IPR036188(FAD/NAD(P)-binding domain superfamily)	3.90E-18
PQK11355.1	IPR036259(MFS transporter superfamily)	1.06E-65
PQK15679.1	IPR036259(MFS transporter superfamily)	1.57E-27
PQK11501.1	IPR036291(NAD(P)-binding domain superfamily)	2.50E-21
PQK13109.1	IPR036291(NAD(P)-binding domain superfamily)	4.07E-26
PQK11562.1	IPR036396(Cytochrome P450 superfamily)	2.23E-86
PQK14904.1	IPR036396(Cytochrome P450 superfamily)	1.00E-102
PQK07920.1	IPR036514(SGNH hydrolase superfamily)	3.20E-37
PQK09677.1	IPR036525(Tubulin/FtsZ, GTPase domain superfamily)	5.30E-97
PQK11372.1	IPR036525(Tubulin/FtsZ, GTPase domain superfamily)	1.00E-118
PQK14877.1	IPR036526(Carbon-nitrogen hydrolase superfamily)	2.40E-43
PQK14878.1	IPR036526(Carbon-nitrogen hydrolase superfamily)	2.00E-43
PQK14879.1	IPR036526(Carbon-nitrogen hydrolase superfamily)	1.10E-11
PQK10519.1	IPR036640(ABC transporter type 1, transmembrane domain superfamily)	5.36E-33
PQK14268.1	IPR036716(Pesticidal crystal protein, N-terminal domain superfamily)	7.60E-38
PQK09985.1	IPR036770(Ankyrin repeat-containing domain superfamily)	1.64E-74
PQK10145.1	IPR036770(Ankyrin repeat-containing domain superfamily)	3.40E-25
PQK10239.1	IPR036770(Ankyrin repeat-containing domain superfamily)	9.85E-36
PQK11915.1	IPR036770(Ankyrin repeat-containing domain superfamily)	3.74E-63
PQK16861.1	IPR036770(Ankyrin repeat-containing domain superfamily)	1.14E-30
PQK10805.1	IPR036801(Diphtheria toxin, translocation domain superfamily)	9.20E-08
PQK16835.1	IPR036962(Glycoside hydrolase, family 3, N-terminal domain superfamily)	1.60E-122
PQK10246.1	IPR039261(Ferredoxin-NADP reductase (FNR), nucleotide-binding domain)	4.20E-12
PQK18001.1	IPR039261(Ferredoxin-NADP reductase (FNR), nucleotide-binding domain)	1.30E-10
PQK07967.1	IPR039430(Thymidylate kinase-like domain)	6.40E-34
PQK08000.1	IPR040976(Fungal-type protein kinase)	1.40E-129
PQK10247.1	IPR040976(Fungal-type protein kinase)	3.60E-127
PQK10144.1	IPR041472(BL00235/CARNS1, N-terminal)	2.80E-10
PQK07962.1	IPR042099(AMP-dependent synthetase-like superfamily)	1.30E-107

**2007 population**		
**Gene ID**	**Annotation**	**Probability***
PQK09171.1	Putative effector	0.778
PQK09679.1	Putative effector	0.595
PQK10125.1	Putative effector	0.848
PQK10249.1	Putative effector	0.56
PQK11371.1	Putative effector	0.621
PQK13230.1	Putative effector	0.723
PQK13756.1	Putative effector	0.638
PQK14301.1	Putative effector	0.71
PQK14447.1	Putative effector	0.781
PQK16844.1	Putative effector	0.732

PQK16862.1	Putative effector	0.728
PQK17161.1	Putative effector	0.598
PQK17162.1	Putative effector	0.604
PQK18089.1	Putative effector	0.581
PQK18125.1	Putative effector	0.827
PQK09172.1	Hypothetical protein	
PQK09585.1	Hypothetical protein	
PQK09691.1	Hypothetical protein	
PQK09694.1	Hypothetical protein	
PQK10241.1	Hypothetical protein	
PQK10242.1	Hypothetical protein	
PQK10804.1	Hypothetical protein	
PQK10806.1	Hypothetical protein	
PQK11425.1	Hypothetical protein	
PQK11512.1	Hypothetical protein	
PQK11671.1	Hypothetical protein	
PQK12989.1	Hypothetical protein	
PQK13168.1	Hypothetical protein	
PQK13232.1	Hypothetical protein	
PQK13668.1	Hypothetical protein	
PQK13673.1	Hypothetical protein	
PQK13809.1	Hypothetical protein	
PQK14125.1	Hypothetical protein	
PQK14416.1	Hypothetical protein	
PQK14519.1	Hypothetical protein	
PQK14876.1	Hypothetical protein	
PQK15396.1	Hypothetical protein	
PQK15471.1	Hypothetical protein	
PQK15630.1	Hypothetical protein	
PQK15631.1	Hypothetical protein	
PQK15632.1	Hypothetical protein	
PQK15633.1	Hypothetical protein	
PQK15637.1	Hypothetical protein	
PQK15804.1	Hypothetical protein	
PQK16329.1	Hypothetical protein	
PQK16774.1	Hypothetical protein	
PQK16775.1	Hypothetical protein	
PQK16776.1	Hypothetical protein	
PQK16800.1	Hypothetical protein	
PQK16841.1	Hypothetical protein	
PQK16849.1	Hypothetical protein	
PQK16850.1	Hypothetical protein	
PQK16856.1	Hypothetical protein	
PQK16860.1	Hypothetical protein	
PQK16879.1	Hypothetical protein	
PQK17032.1	Hypothetical protein	
PQK17439.1	Hypothetical protein	
PQK17610.1	Hypothetical protein	
PQK17985.1	Hypothetical protein	
PQK17993.1	Hypothetical protein	
PQK17994.1	Hypothetical protein	
PQK17998.1	Hypothetical protein	
PQK17999.1	Hypothetical protein	
PQK18002.1	Hypothetical protein	
PQK18089.1	Hypothetical protein	
PQK18122.1	Hypothetical protein	
PQK18004.1	IPR000477(Reverse transcriptase domain)	8.20E-29

PQK18006.1	IPR000477(Reverse transcriptase domain)	3.70E-40
PQK13607.1	IPR000555(JAB1/MPN/MOV34 metalloenzyme domain)	3.80E-14
PQK13675.1	IPR000719(Protein kinase domain)	1.90E-87
PQK13066.1	IPR001086(Prephenate dehydratase)	2.40E-49
PQK13670.1	IPR001138(Zn(2)-C6 fungal-type DNA-binding domain)	1.63E-11
PQK16687.1	IPR001144(Heat-labile enterotoxin, A chain)	1.30E-15
PQK17160.1	IPR001144(Heat-labile enterotoxin, A chain)	1.90E-46
PQK12982.1	IPR001401(Dynamin, GTPase domain)	5.62E-91
PQK13749.1	IPR001401(Dynamin, GTPase domain)	5.61E-80
PQK16790.1	IPR001401(Dynamin, GTPase domain)	1.12E-95
PQK16855.1	IPR001401(Dynamin, GTPase domain)	6.14E-90
PQK10146.1	IPR003615(HNH nuclease)	7.90E-06
PQK09197.1	IPR003898(Bordetella pertussis toxin A)	1.00E-04
PQK13169.1	IPR004006(DhaK domain)	8.40E-97
PQK11966.1	IPR004148(BAR domain)	3.10E-68
PQK16233.1	IPR004314(Neprosin)	4.50E-51
PQK14725.1	IPR004418(Homoaconitase, mitochondrial)	0
PQK13672.1	IPR005828(Major facilitator, sugar transporter-like)	2.70E-89
PQK10148.1	IPR006274(Carbamoyl-phosphate synthase, small subunit)	4.50E-134
PQK12956.1	IPR006414(P-type ATPase, subfamily IID)	2.50E-302
PQK11628.1	IPR006539(P-type ATPase, subfamily IV)	1.00E-153
PQK11387.1	IPR006598(Glycosyl transferase CAP10 domain)	1.20E-11
PQK17944.1	IPR006620(Prolyl 4-hydroxylase, alpha subunit)	5.90E-23
PQK09202.1	IPR007197(Radical SAM)	0
PQK09116.1	IPR007219(Transcription factor domain, fungi)	3.80E-10
PQK09690.1	IPR007219(Transcription factor domain, fungi)	1.80E-36
PQK10020.1	IPR007219(Transcription factor domain, fungi)	1.70E-19
PQK11566.1	IPR007219(Transcription factor domain, fungi)	1.60E-12
PQK13846.1	IPR007219(Transcription factor domain, fungi)	9.90E-14
PQK15625.1	IPR007219(Transcription factor domain, fungi)	2.00E-10
PQK14956.1	IPR007306(tRNA A64-2'-O-ribosylphosphate transferase)	3.20E-125
PQK14875.1	IPR007599(Derlin)	4.20E-21
PQK12361.1	IPR008422(Homeobox KN domain)	4.70E-17
PQK15129.1	IPR008427(Extracellular membrane protein, CFEM domain)	1.00E-05
PQK14724.1	IPR008949(Isoprenoid synthase domain superfamily)	4.10E-73
PQK15619.1	IPR008949(Isoprenoid synthase domain superfamily)	2.20E-89
PQK09192.1	IPR009078(Ferritin-like superfamily)	2.61E-14
PQK11967.1	IPR010240(Cysteine desulfurase IscS)	6.50E-199
PQK11895.1	IPR010255(Haem peroxidase superfamily)	1.47E-49
PQK16833.1	IPR010730(Heterokaryon incompatibility)	9.20E-38
PQK08975.1	IPR011009(Protein kinase-like domain superfamily)	2.36E-48
PQK08977.1	IPR011009(Protein kinase-like domain superfamily)	1.30E-23
PQK09127.1	IPR011009(Protein kinase-like domain superfamily)	2.42E-07
PQK10048.1	IPR011009(Protein kinase-like domain superfamily)	1.78E-06
PQK14373.1	IPR011009(Protein kinase-like domain superfamily)	8.93E-06
PQK15805.1	IPR011009(Protein kinase-like domain superfamily)	4.27E-22
PQK15975.1	IPR011009(Protein kinase-like domain superfamily)	2.04E-11
PQK16770.1	IPR011009(Protein kinase-like domain superfamily)	1.80E-05
PQK11965.1	IPR011037(Pyruvate kinase-like, insert domain superfamily)	8.89E-62
PQK08527.1	IPR011141(Polyketide synthase, type III)	6.20E-72
PQK11565.1	IPR011989(Armadillo-like helical)	0
PQK16803.1	IPR011990(Tetratricopeptide-like helical domain superfamily)	1.10E-22
PQK11669.1	IPR012334(Pectin lyase fold)	8.00E-122
PQK15635.1	IPR012337(Ribonuclease H-like superfamily)	3.41E-26
PQK09610.1	IPR012719(T-complex protein 1, gamma subunit)	0
PQK17991.1	IPR013087(Zinc finger C2H2-type)	1.3
PQK15636.1	IPR013530(Protein-arginine deiminase, C-terminal)	2.00E-112

PQK17724.1	IPR013876(TFIIH p62 subunit, N-terminal)	1.10E-24
PQK16266.1	IPR014808(DNA replication factor Dna2, N-terminal)	9.80E-71
PQK11567.1	IPR015421(Pyridoxal phosphate-dependent transferase, major domain)	2.50E-114
PQK14063.1	IPR015421(Pyridoxal phosphate-dependent transferase, major domain)	4.00E-96
PQK13065.1	IPR015943(WD40/YVTN repeat-like-containing domain superfamily)	6.20E-79
PQK09025.1	IPR017853(Glycoside hydrolase superfamily)	1.10E-61
PQK10788.1	IPR019325(NEDD4/Bsd2)	3.20E-89
PQK16878.1	IPR019410(Lysine methyltransferase)	1.80E-28
PQK09212.1	IPR019626(Stress-induced protein, KGG, repeat)	9.60E-12
PQK08526.1	IPR020841(Polyketide synthase, beta-ketoacyl synthase domain)	9.70E-165
PQK13624.1	IPR021054(Cell wall mannoprotein 1)	8.60E-22
PQK10154.1	IPR021109(Aspartic peptidase domain superfamily)	4.98E-24
PQK16784.1	IPR021514(Protein of unknown function DUF3176)	1.00E-28
PQK17992.1	IPR021711(Protein of unknown function DUF3295)	2.20E-39
PQK09693.1	IPR021765(Mycotoxin biosynthesis protein UstYa-like)	2.60E-48
PQK10238.1	IPR021842(Protein of unknown function DUF3435)	3.70E-52
PQK12981.1	IPR021842(Protein of unknown function DUF3435)	1.60E-15
PQK15629.1	IPR021842(Protein of unknown function DUF3435)	2.10E-60
PQK16853.1	IPR021842(Protein of unknown function DUF3435)	2.10E-54
PQK08765.1	IPR022099(Protein of unknown function DUF3638)	4.20E-94
PQK16015.1	IPR022099(Protein of unknown function DUF3638)	1.40E-72
PQK16859.1	IPR022099(Protein of unknown function DUF3638)	1.50E-46
PQK16854.1	IPR022190(Protein of unknown function DUF3716)	4.10E-07
PQK10244.1	IPR022198(Protein of unknown function DUF3723)	1.90E-163
PQK18003.1	IPR022198(Protein of unknown function DUF3723)	9.40E-172
PQK14957.1	IPR023198(Phosphoglycolate phosphatase-like, domain 2)	1.40E-45
PQK16365.1	IPR023214(HAD superfamily)	6.10E-71
PQK15978.1	IPR023465(Riboflavin kinase domain superfamily)	5.80E-20
PQK16842.1	IPR023606(CoA-transferase family III domain superfamily)	1.83E-51
PQK11219.1	IPR023621(Ribosomal protein L31e domain superfamily)	2.90E-50
PQK11964.1	IPR024326(Ribosomal RNA-processing protein 7, C-terminal domain)	1.10E-27
PQK16777.1	IPR025213(Kinetochore Sim4 complex subunit Fta2)	6.00E-10
PQK09866.1	IPR027417(P-loop containing nucleoside triphosphate hydrolase)	3.15E-34
PQK10250.1	IPR027417(P-loop containing nucleoside triphosphate hydrolase)	2.50E-32
PQK14607.1	IPR027417(P-loop containing nucleoside triphosphate hydrolase)	3.29E-63
PQK14994.1	IPR027417(P-loop containing nucleoside triphosphate hydrolase)	5.67E-42
PQK16243.1	IPR027417(P-loop containing nucleoside triphosphate hydrolase)	1.14E-30
PQK16766.1	IPR027417(P-loop containing nucleoside triphosphate hydrolase)	6.48E-26
PQK09160.1	IPR029058(Alpha/Beta hydrolase fold)	7.30E-40
PQK09547.1	IPR029058(Alpha/Beta hydrolase fold)	2.10E-61
PQK15977.1	IPR029058(Alpha/Beta hydrolase fold)	1.00E-21
PQK16852.1	IPR029058(Alpha/Beta hydrolase fold)	1.37E-13
PQK09117.1	IPR029063(S-adenosyl-L-methionine-dependent methyltransferase)	2.96E-26
PQK09696.1	IPR029063(S-adenosyl-L-methionine-dependent methyltransferase)	2.76E-15
PQK18088.1	IPR029068(Glyoxalase/Bleomycin resistance protein/Dihydroxybiphenyl dioxygenase)	2.00E-36
PQK09159.1	IPR030185(Malic acid transport protein)	1.08E-112
PQK15534.1	IPR031359(NWD NACHT-NTPase, N-terminal)	1.40E-41
PQK15624.1	IPR031359(NWD NACHT-NTPase, N-terminal)	1.60E-25
PQK12417.1	IPR032430(Proteasome activator Blm10, mid region)	5.20E-194
PQK15628.1	IPR032675(Leucine-rich repeat domain superfamily)	1.50E-22
PQK15131.1	IPR033876(Secreted aspartic endopeptidase)	9.51E-74
PQK14316.1	IPR034003(ATP-binding cassette transporter, PDR-like subfamily G, domain 2)	4.79E-111
PQK08244.1	IPR034187(PoSI-like peptidase domain)	6.80E-109
PQK16356.1	IPR035445(GYF-like domain superfamily)	1.10E-20
PQK08708.1	IPR035994(Nucleoside phosphorylase superfamily)	1.11E-30
PQK16765.1	IPR035994(Nucleoside phosphorylase superfamily)	8.81E-38

PQK09158.1	IPR036188(FAD/NAD(P)-binding domain superfamily)	4.00E-72
PQK13845.1	IPR036188(FAD/NAD(P)-binding domain superfamily)	1.10E-55
PQK16773.1	IPR036188(FAD/NAD(P)-binding domain superfamily)	3.90E-18
PQK09543.1	IPR036259(MFS transporter superfamily)	7.85E-57
PQK09669.1	IPR036259(MFS transporter superfamily)	6.80E-25
PQK09695.1	IPR036259(MFS transporter superfamily)	2.22E-34
PQK10137.1	IPR036259(MFS transporter superfamily)	1.23E-19
PQK13669.1	IPR036259(MFS transporter superfamily)	8.89E-49
PQK15679.1	IPR036259(MFS transporter superfamily)	1.57E-27
PQK15667.1	IPR036318(FAD-binding, type PCMH-like superfamily)	1.26E-30
PQK16349.1	IPR036322(WD40-repeat-containing domain superfamily)	3.98E-29
PQK11562.1	IPR036396(Cytochrome P450 superfamily)	2.23E-86
PQK14723.1	IPR036396(Cytochrome P450 superfamily)	8.38E-65
PQK16847.1	IPR036397(Ribonuclease H superfamily)	2.00E-20
PQK12179.1	IPR036465(von Willebrand factor A-like domain superfamily)	1.40E-21
PQK14877.1	IPR036526(Carbon-nitrogen hydrolase superfamily)	2.40E-43
PQK10519.1	IPR036640(ABC transporter type 1, transmembrane domain superfamily)	5.36E-33
PQK10145.1	IPR036770(Ankyrin repeat-containing domain superfamily)	3.40E-25
PQK10239.1	IPR036770(Ankyrin repeat-containing domain superfamily)	9.85E-36
PQK12962.1	IPR036770(Ankyrin repeat-containing domain superfamily)	1.06E-52
PQK14249.1	IPR036770(Ankyrin repeat-containing domain superfamily)	7.82E-42
PQK16861.1	IPR036770(Ankyrin repeat-containing domain superfamily)	1.14E-30
PQK16213.1	IPR036864(Zn(2)-C6 fungal-type DNA-binding domain superfamily)	5.60E-10
PQK16835.1	IPR036962(Glycoside hydrolase, family 3, N-terminal domain superfamily)	1.60E-122
PQK09670.1	IPR037217(Tryptophan/Indoleamine 2,3-dioxygenase-like)	3.34E-86
PQK09692.1	IPR037671(GPI ethanolamine phosphate transferase 1, N-terminal)	5.85E-167
PQK09602.1	IPR037986(Dilute domain-containing protein, fungi, CBD domain)	0
PQK10337.1	IPR038633(Proteasomal ubiquitin receptor Rpn13/ADRM1 superfamily)	2.80E-29
PQK13233.1	IPR038656(Peptidase G1 superfamily)	1.50E-76
PQK14065.1	IPR038694(Superfamily of unknown function DUF427)	7.30E-28
PQK10246.1	IPR039261(Ferredoxin-NADP reductase (FNR), nucleotide-binding domain)	4.20E-12
PQK11572.1	IPR039261(Ferredoxin-NADP reductase (FNR), nucleotide-binding domain)	1.20E-22
PQK18001.1	IPR039261(Ferredoxin-NADP reductase (FNR), nucleotide-binding domain)	1.30E-10
PQK10247.1	IPR040976(Fungal-type protein kinase)	3.60E-127
PQK16354.1	IPR041728(GPAT/DHAPAT, acyltransferase domain)	2.05E-92
PQK10038.1	IPR042099(AMP-dependent synthetase-like superfamily)	4.70E-77
PQK15382.1	IPR042099(AMP-dependent synthetase-like superfamily)	3.30E-108

**2017 population**		
**Gene ID**	**Annotation**	**Probability***
PQK09384.1	Putative effector	0.763
PQK09679.1	Putative effector	0.595
PQK10134.1	Putative effector	0.748
PQK10711.1	Putative effector	0.673
PQK10779.1	Putative effector	0.559
PQK11371.1	Putative effector	0.621
PQK11499.1	Putative effector	0.594
PQK11670.1	Putative effector	0.642
PQK13230.1	Putative effector	0.723
PQK13756.1	Putative effector	0.638
PQK14374.1	Putative effector	0.551
PQK16000.1	Putative effector	0.584
PQK16844.1	Putative effector	0.732
PQK16862.1	Putative effector	0.728
PQK17161.1	Putative effector	0.598
PQK17162.1	Putative effector	0.604

PQK18051.1	Putative effector	0.593
PQK08792.1	Hypothetical protein	
PQK09101.1	Hypothetical protein	
PQK09161.1	Hypothetical protein	
PQK09168.1	Hypothetical protein	
PQK09550.1	Hypothetical protein	
PQK09585.1	Hypothetical protein	
PQK09940.1	Hypothetical protein	
PQK10241.1	Hypothetical protein	
PQK10242.1	Hypothetical protein	
PQK10252.1	Hypothetical protein	
PQK10686.1	Hypothetical protein	
PQK11342.1	Hypothetical protein	
PQK11425.1	Hypothetical protein	
PQK11671.1	Hypothetical protein	
PQK12989.1	Hypothetical protein	
PQK13148.1	Hypothetical protein	
PQK13158.1	Hypothetical protein	
PQK13168.1	Hypothetical protein	
PQK13232.1	Hypothetical protein	
PQK13809.1	Hypothetical protein	
PQK14317.1	Hypothetical protein	
PQK14416.1	Hypothetical protein	
PQK14701.1	Hypothetical protein	
PQK14876.1	Hypothetical protein	
PQK15471.1	Hypothetical protein	
PQK15630.1	Hypothetical protein	
PQK15631.1	Hypothetical protein	
PQK15632.1	Hypothetical protein	
PQK15633.1	Hypothetical protein	
PQK15637.1	Hypothetical protein	
PQK15639.1	Hypothetical protein	
PQK15804.1	Hypothetical protein	
PQK16774.1	Hypothetical protein	
PQK16775.1	Hypothetical protein	
PQK16776.1	Hypothetical protein	
PQK16800.1	Hypothetical protein	
PQK16841.1	Hypothetical protein	
PQK16843.1	Hypothetical protein	
PQK16849.1	Hypothetical protein	
PQK16850.1	Hypothetical protein	
PQK16851.1	Hypothetical protein	
PQK16856.1	Hypothetical protein	
PQK16860.1	Hypothetical protein	
PQK17167.1	Hypothetical protein	
PQK17538.1	Hypothetical protein	
PQK17985.1	Hypothetical protein	
PQK17993.1	Hypothetical protein	
PQK17994.1	Hypothetical protein	
PQK18002.1	Hypothetical protein	
PQK18052.1	Hypothetical protein	
PQK18055.1	Hypothetical protein	
PQK16112.1	IPR000115(Phosphoribosylglycinamide synthetase)	9.60E-161
PQK10141.1	IPR000277(Cys/Met metabolism, pyridoxal phosphate-dependent enzyme)	7.50E-115
PQK18004.1	IPR000477(Reverse transcriptase domain)	8.20E-29
PQK18006.1	IPR000477(Reverse transcriptase domain)	3.70E-40
PQK10710.1	IPR000558(Histone H2B)	1.10E-70

PQK17754.1	IPR000719(Protein kinase domain)	1.80E-101
PQK15088.1	IPR001002(Chitin-binding, type 1)	2.20E-08
PQK16687.1	IPR001144(Heat-labile enterotoxin, A chain)	1.30E-15
PQK09986.1	IPR001214(SET domain)	5.60E-10
PQK16831.1	IPR001214(SET domain)	5.80E-09
PQK09941.1	IPR001254(Serine proteases, trypsin domain)	5.64E-57
PQK15772.1	IPR001254(Serine proteases, trypsin domain)	1.88E-62
PQK16790.1	IPR001401(Dynamin, GTPase domain)	1.12E-95
PQK16855.1	IPR001401(Dynamin, GTPase domain)	6.14E-90
PQK15118.1	IPR002293(Amino acid/polyamine transporter I)	5.30E-54
PQK16330.1	IPR002889(Carbohydrate-binding WSC)	1.40E-13
PQK17452.1	IPR002889(Carbohydrate-binding WSC)	2.00E-19
PQK09398.1	IPR003084(Histone deacetylase)	3.50E-170
PQK17374.1	IPR003719(Phenazine biosynthesis PhzF protein)	2.40E-49
PQK10143.1	IPR004298(Nicotianamine synthase)	3.50E-25
PQK10706.1	IPR004331(SPX domain)	2.20E-62
PQK15094.1	IPR004648(Tetrapeptide transporter, OPT1/isp4)	1.20E-197
PQK14058.1	IPR005828(Major facilitator, sugar transporter-like)	5.90E-70
PQK11628.1	IPR006539(P-type ATPase, subfamily IV)	1.00E-153
PQK10910.1	IPR006845(Pex, N-terminal)	1.70E-37
PQK17375.1	IPR007196(CCR4-Not complex component, Not1, C-terminal)	2.90E-122
PQK13846.1	IPR007219(Transcription factor domain, fungi)	9.90E-14
PQK15625.1	IPR007219(Transcription factor domain, fungi)	2.00E-10
PQK16319.1	IPR007219(Transcription factor domain, fungi)	1.40E-10
PQK08978.1	IPR007577(Glycosyltransferase, DXD sugar-binding motif)	2.40E-17
PQK15129.1	IPR008427(Extracellular membrane protein, CFEM domain)	1.00E-05
PQK16814.1	IPR008630(Glycosyltransferase 34)	9.80E-11
PQK14057.1	IPR008794(Proline racemase family)	0
PQK09192.1	IPR009078(Ferritin-like superfamily)	2.61E-14
PQK09681.1	IPR009992(Trichothecene 15-O-acetyltransferase Tri3/Sat12/Sat16)	2.30E-218
PQK10701.1	IPR010490(Conserved oligomeric Golgi complex subunit 6)	3.50E-181
PQK16833.1	IPR010730(Heterokaryon incompatibility)	9.20E-38
PQK10687.1	IPR010770(Ecd family)	2.10E-75
PQK09127.1	IPR011009(Protein kinase-like domain superfamily)	2.42E-07
PQK09298.1	IPR011009(Protein kinase-like domain superfamily)	4.42E-05
PQK09605.1	IPR011009(Protein kinase-like domain superfamily)	1.21E-47
PQK09955.1	IPR011009(Protein kinase-like domain superfamily)	1.57E-36
PQK14373.1	IPR011009(Protein kinase-like domain superfamily)	8.93E-06
PQK15638.1	IPR011009(Protein kinase-like domain superfamily)	3.06E-07
PQK15805.1	IPR011009(Protein kinase-like domain superfamily)	4.27E-22
PQK16770.1	IPR011009(Protein kinase-like domain superfamily)	1.80E-05
PQK17988.1	IPR011009(Protein kinase-like domain superfamily)	3.01E-10
PQK08527.1	IPR011141(Polyketide synthase, type III)	6.20E-72
PQK17293.1	IPR011650(Peptidase M20, dimerisation domain)	4.60E-19
PQK10684.1	IPR012110(Thiamine pyrophosphate (TPP)-dependent enzyme)	2.50E-215
PQK11669.1	IPR012334(Pectin lyase fold)	8.00E-122
PQK15635.1	IPR012337(Ribonuclease H-like superfamily)	3.41E-26
PQK10702.1	IPR012340(Nucleic acid-binding, OB-fold)	2.83E-27
PQK17991.1	IPR013087(Zinc finger C2H2-type)	1.3
PQK15636.1	IPR013530(Protein-arginine deiminase, C-terminal)	2.00E-112
PQK14422.1	IPR013694(VIT domain)	2.80E-33
PQK10382.1	IPR013863(Vacuolar import/degradation Vid27, C-terminal)	1.30E-191
PQK17756.1	IPR013878(Mo25-like)	7.70E-129
PQK18087.1	IPR014710(RmlC-like jelly roll fold)	4.00E-45
PQK08310.1	IPR014848(Reduced growth phenotype protein 1)	2.00E-156
PQK16772.1	IPR015421(Pyridoxal phosphate-dependent transferase, major domain)	9.40E-70
PQK10705.1	IPR015424(Pyridoxal phosphate-dependent transferase)	2.11E-51

PQK10136.1	IPR015943(WD40/YVTN repeat-like-containing domain superfamily)	1.40E-44
PQK15117.1	IPR015943(WD40/YVTN repeat-like-containing domain superfamily)	1.30E-87
PQK15605.1	IPR016024(Armadillo-type fold)	4.76E-40
PQK17623.1	IPR016039(Thiolase-like)	1.10E-111
PQK09386.1	IPR017261(DNA mismatch repair protein MutS/MSH)	1.60E-56
PQK09025.1	IPR017853(Glycoside hydrolase superfamily)	1.10E-61
PQK15677.1	IPR018871(GLEYA adhesin domain)	1.00E-24
PQK08526.1	IPR020841(Polyketide synthase, beta-ketoacyl synthase domain)	9.70E-165
PQK11627.1	IPR021110(DNA replication/checkpoint protein)	1.50E-82
PQK16784.1	IPR021514(Protein of unknown function DUF3176)	1.00E-28
PQK17992.1	IPR021711(Protein of unknown function DUF3295)	2.20E-39
PQK09693.1	IPR021765(Mycotoxin biosynthesis protein UstYa-like)	2.60E-48
PQK10238.1	IPR021842(Protein of unknown function DUF3435)	3.70E-52
PQK11369.1	IPR021842(Protein of unknown function DUF3435)	3.70E-57
PQK15629.1	IPR021842(Protein of unknown function DUF3435)	2.10E-60
PQK17359.1	IPR021858(Fungal transcription factor)	7.60E-19
PQK16015.1	IPR022099(Protein of unknown function DUF3638)	1.40E-72
PQK16859.1	IPR022099(Protein of unknown function DUF3638)	1.50E-46
PQK16854.1	IPR022190(Protein of unknown function DUF3716)	4.10E-07
PQK10244.1	IPR022198(Protein of unknown function DUF3723)	1.90E-163
PQK18003.1	IPR022198(Protein of unknown function DUF3723)	9.40E-172
PQK16842.1	IPR023606(CoA-transferase family III domain superfamily)	1.83E-51
PQK14537.1	IPR024077(Neurolysin/Thimet oligopeptidase, domain 2)	2.10E-26
PQK16777.1	IPR025213(Kinetochore Sim4 complex subunit Fta2)	6.00E-10
PQK11576.1	IPR027281(Saccharopine dehydrogenase [NAD(+), L-lysine-forming])	5.79E-180
PQK10140.1	IPR027417(P-loop containing nucleoside triphosphate hydrolase)	4.17E-61
PQK12032.1	IPR027417(P-loop containing nucleoside triphosphate hydrolase)	5.54E-17
PQK13955.1	IPR027417(P-loop containing nucleoside triphosphate hydrolase)	2.56E-39
PQK14994.1	IPR027417(P-loop containing nucleoside triphosphate hydrolase)	5.67E-42
PQK15641.1	IPR027417(P-loop containing nucleoside triphosphate hydrolase)	5.40E-32
PQK15634.1	IPR027796(OTT_1508-like deaminase)	3.20E-06
PQK18049.1	IPR027796(OTT_1508-like deaminase)	2.10E-18
PQK10703.1	IPR029026(tRNA (guanine-N1-)-methyltransferase, N-terminal)	2.60E-99
PQK09160.1	IPR029058(Alpha/Beta hydrolase fold)	7.30E-40
PQK16769.1	IPR029058(Alpha/Beta hydrolase fold)	1.28E-13
PQK11626.1	IPR029063(S-adenosyl-L-methionine-dependent methyltransferase)	1.52E-56
PQK10704.1	IPR029068(Glyoxalase/Bleomycin resistance protein/Dihydroxybiphenyl dioxygenase)	6.90E-09
PQK09159.1	IPR030185(Malic acid transport protein)	1.08E-112
PQK15624.1	IPR031359(NWD NACHT-NTPase, N-terminal)	1.60E-25
PQK15628.1	IPR032675(Leucine-rich repeat domain superfamily)	1.50E-22
PQK14316.1	IPR034003(ATP-binding cassette transporter, PDR-like subfamily G, domain 2)	4.79E-111
PQK14315.1	IPR034016(Aminopeptidase N-type)	0
PQK10709.1	IPR034082(Protein SQS1, R3H domain)	5.71E-19
PQK08244.1	IPR034187(PoSI-like peptidase domain)	6.80E-109
PQK13231.1	IPR034187(PoSI-like peptidase domain)	2.04E-104
PQK10690.1	IPR035979(RNA-binding domain superfamily)	1.76E-25
PQK09158.1	IPR036188(FAD/NAD(P)-binding domain superfamily)	4.00E-72
PQK10142.1	IPR036188(FAD/NAD(P)-binding domain superfamily)	1.40E-60
PQK13845.1	IPR036188(FAD/NAD(P)-binding domain superfamily)	1.10E-55
PQK16773.1	IPR036188(FAD/NAD(P)-binding domain superfamily)	3.90E-18
PQK18035.1	IPR036236(Zinc finger C2H2 superfamily)	3.79E-07
PQK07960.1	IPR036259(MFS transporter superfamily)	1.19E-18
PQK07961.1	IPR036259(MFS transporter superfamily)	5.62E-52
PQK10137.1	IPR036259(MFS transporter superfamily)	1.23E-19
PQK10882.1	IPR036259(MFS transporter superfamily)	2.35E-63

PQK11485.1	IPR036259(MFS transporter superfamily)	3.27E-18
PQK11673.1	IPR036259(MFS transporter superfamily)	3.92E-51
PQK11917.1	IPR036259(MFS transporter superfamily)	5.75E-72
PQK10728.1	IPR036291(NAD(P)-binding domain superfamily)	3.12E-44
PQK14428.1	IPR036291(NAD(P)-binding domain superfamily)	8.39E-34
PQK08552.1	IPR036322(WD40-repeat-containing domain superfamily)	7.32E-63
PQK09193.1	IPR036322(WD40-repeat-containing domain superfamily)	4.66E-69
PQK09540.1	IPR036322(WD40-repeat-containing domain superfamily)	7.87E-25
PQK09714.1	IPR036396(Cytochrome P450 superfamily)	1.10E-147
PQK14061.1	IPR036396(Cytochrome P450 superfamily)	8.00E-98
PQK16847.1	IPR036397(Ribonuclease H superfamily)	2.00E-20
PQK09677.1	IPR036525(Tubulin/FtsZ, GTPase domain superfamily)	5.30E-97
PQK14877.1	IPR036526(Carbon-nitrogen hydrolase superfamily)	2.40E-43
PQK14879.1	IPR036526(Carbon-nitrogen hydrolase superfamily)	1.10E-11
PQK09195.1	IPR036691(Endonuclease/exonuclease/phosphatase superfamily)	4.70E-110
PQK09588.1	IPR036770(Ankyrin repeat-containing domain superfamily)	1.50E-29
PQK09985.1	IPR036770(Ankyrin repeat-containing domain superfamily)	1.64E-74
PQK10239.1	IPR036770(Ankyrin repeat-containing domain superfamily)	9.85E-36
PQK16861.1	IPR036770(Ankyrin repeat-containing domain superfamily)	1.14E-30
PQK10707.1	IPR036869(Chaperone J-domain superfamily)	5.00E-24
PQK17755.1	IPR036885(SWIB/MDM2 domain superfamily)	3.60E-06
PQK14318.1	IPR036928(Amidase signature (AS) superfamily)	7.80E-152
PQK16835.1	IPR036962(Glycoside hydrolase, family 3, N-terminal domain superfamily)	1.60E-122
PQK10708.1	IPR038765(Papain-like cysteine peptidase superfamily)	2.75E-100
PQK10729.1	IPR038765(Papain-like cysteine peptidase superfamily)	1.71E-77
PQK18001.1	IPR039261(Ferredoxin-NADP reductase (FNR), nucleotide-binding domain)	1.30E-10
PQK10685.1	IPR040183(THUMP domain-containing protein 1-like)	3.59E-45
PQK15640.1	IPR040976(Fungal-type protein kinase)	1.40E-126
PQK10144.1	IPR041472(BL00235/CARNS1, N-terminal)	2.80E-10

*, EffectorP analysis for putative effectors or InterproScan analysis for conserved domain.
