# Supplementary material for: Population genomics and evolution of a fungal pathogen after releasing exotic strains to control insect pests for 20 years
Source: ISME J. 2020 Feb 28;14(6):1422–34. doi: 10.1038/s41396-020-0620-8 (PMC7242398; doi:10.1038/s41396-020-0620-8)
Supplement: Supplementary file 19 — Table S10 [file 41396_2020_620_MOESM19_ESM.pdf]

Table S10. Different batches of mating test between the opposite mating-type isolates.

MAT1-1	MAT1-2	MAT1-1	MAT1-2	MAT1-1	MAT1-2	MAT1-1	MAT1-2
**Bb175**	Bb13	**Bb165**	**Bb175**	Bb174	**Bb13**	Bb205	**Bb17**
	Bb17	Bb168		Bb211		Bb165	
	Bb205	**Bb175**		Bb124		Bb267	
	**Bb175**	Bb205		Bb122		Bb168	
	Bb144	Bb219		Bb242		Bb269	
	Bb243	Bb252		Bb2007		Bb252	
	Bb1983	**Bb269**		Bb2056		Bb2071	
	Bb2006	Bb2006		**Bb2136**		Bb2050	
	Bb2049	Bb2052		Bb2138		Bb2060	
	Bb2055	**Bb2060**		Bb2063		Bb2147	
	ARSEF 8028	Bb2137		Bb2001		Bb3232	
	Bb2015	Bb2147		Bb1982		Bb3235	
	Bb2073	Bb3229		Bb2000		**Bb3245**	
	Bb2112	Bb3232				Bb3246	
		Bb3235				**Bb3249**	
		Bb3236				Bb3264	
		Bb3245				Bb3268	
		**Bb3246**				Bb3273	
		Bb3246					
		Bb3249					
		Bb3250					
		Bb3254					
		Bb3260					
		Bb3264					
		Bb3267					
		Bb3268					
		Bb3270					
		Bb3273					
		Bb3274					

Note: The isolates labelled in bold in each batch (highlighted in the same background colour) could form sexual fruiting bodies.
